# Historical records in solar physics

**DOI:** 10.1007/s41116-026-00044-9

**Published:** 2026-09-08

**Authors:** Theodosios Chatzistergos

**Affiliations:** Max Planck Institute for Solar System Research, Justus-von-Liebig-Weg, 337077 Göttingen, Germany

**Keywords:** Solar activity, Long-term reconstructions, Solar observations, Solar irradiance

## Abstract

The Sun has been observed for millennia, but systematic and quantitative records of its activity started being collected with the advent of telescopic observations in the early 17th century. These early observations revealed key signatures of solar magnetic activity such as sunspots and faculae. Subsequent advances in instrumentation and observing techniques enabled the study of different layers of the solar atmosphere and led to the establishment of numerous long-term observational programs. These records underpin our current understanding of solar variability across timescales from days to centuries. In this review, I survey the principal long-term historical archives of solar observations and their analyses, where “historical” refers to datasets spanning roughly 50 years or more. Because many of these observations were obtained when both instrumentation and the physical understanding of the Sun were evolving, their interpretation presents significant challenges. Modern analysis methods and improved calibration techniques, however, now allow these legacy datasets to be re-examined and homogenised, enabling the extraction of information that was not fully exploited at the time of their acquisition. I also discuss the main solar activity and variability indices derived from historical observations, as well as reconstructions of other solar parameters based on these records.

## Introduction

Today, it is well established that the Sun, our nearest star, is the primary external energy source to Earth’s system (e.g., Kren et al. 2017). Multiple pathways have been identified through which solar variability may impact Earth’s climate (Haigh 2007; Gray et al. 2010; Solanki and Unruh 2013). On timescales relevant to climate change, from decades to millennia, the most significant mechanism is the variability in the Sun’s radiative output, solar irradiance (Haigh 2007; Gray et al. 2010; Solanki and Unruh 2013; Intergovernmental Panel on Climate Change 2021). On shorter timescales, transient solar phenomena, such as flares and coronal mass ejections (CMEs), generate bursts of highly energetic particles (see review by Desai and Giacalone 2016). These transient and often highly variable phenomena are key drivers of space weather (see review by Temmer 2021). Collectively, these space weather effects can disrupt technological systems, posing serious risks to electrical power grids, satellite functionality, aviation routes, and global communication and navigation networks. These can also alter the ionisation levels and drive chemical processes in Earth’s upper and middle atmosphere, including ozone formation (e.g. Rozanov et al. 2005; Sinnhuber et al. 2012; Sinnhuber and Funke 2020; Mironova et al. 2015). All these mechanisms ultimately stem from the Sun’s dynamic magnetic field, which also shapes the structure of the interplanetary magnetic field (see review by Owens and Forsyth 2013). Having long-term, high-quality records of metrics of solar activity and variability is thus crucial for assessing the Sun’s role in climate variability and change and for improving our predictive capabilities and mitigating the societal and economic risks posed by space weather. Moreover, the Sun’s proximity makes it uniquely accessible: it is the only star whose surface and atmosphere we can observe with fine spatial resolution. As such, the Sun serves as an exceptional natural laboratory for astrophysics. Detailed solar observations not only deepen our understanding of solar processes but also inform the interpretation of more distant stars (e.g., Shapiro et al. 2014; Işik et al. 2018; Reinhold et al. 2020; Sowmya et al. 2021, 2023, 2026; Charbonneau and Sokoloff 2023; Vasilyev et al. 2025, 2026b), which are typically observed as unresolved points of light.

The Sun has indeed been a subject of human curiosity and observation for millennia (e.g. Vaquero and Vázquez 2009). However, systematic and sufficiently accurate records of solar characteristics only became possible with the invention of the telescope in the early 17th century. These early telescopic observations revealed the recurrent appearance of dark sunspots and bright faculae on the solar surface, manifestations of magnetic activity that define the approximately 11-year solar cycle (see review by Hathaway 2015). For more than 200 years since the first telescopic solar observations, the other solar atmospheric layers still remained hidden outside of some fleeting glimpses during solar eclipses. This changed with the total solar eclipse of August 18, 1868. Through independent spectroscopic observations during and following the totality of the eclipse, Joseph Norman Lockyer and Pierre Janssen established that the spectral lines associated with solar prominences are real solar features, which can be investigated spectroscopically at any time and not only during eclipses. This set the basis for performing monochromatic observations of the Sun over different wavelength intervals and thus sampling different altitudes of the solar atmosphere (e.g. Lockyer 1869; Lorenzoni 1872). Since then there have been significant advances in instrumentation and observational strategies. Solar observations are currently supported by a broad suite of sophisticated tools, both ground-based and space-based. Numerous ground-based observatories contribute to long-term synoptic monitoring (which will be discussed in the following sections), while over the last decades space missions such as the Solar and Heliospheric Observatory (SoHO; Domingo et al. 1995), Solar TErrestrial RElations Observatory (STEREO; Kaiser 2005), Solar Dynamics Observatory (SDO; Pesnell et al. 2012), Parker Solar Probe (Fox et al. 2016), Solar Orbiter (SO; Müller et al. 2020), Aditya-L1 (Tripathi et al. 2022), and Polarimeter to Unify the Corona and Heliosphere (PUNCH; DeForest et al. 2026) have delivered continuous, high-resolution data across a wide spectral range.

Nevertheless, not all observational records span sufficiently long timescales. For instance, space-based magnetograms have only been available since 1996 with the Michelson Doppler Imager (MDI; Scherrer et al. 1995) onboard SoHO, and reasonably high-quality ground-based magnetograms date back only to 1974. This limited temporal coverage poses a challenge for studies that seek to identify multidecadal to centennial trends in solar behaviour. However, a rich archive of historical observations of different aspects of solar activity exists, covering, in different extent, the last four centuries. Interestingly many of these records were collected before solar physics matured as a discipline, thus carrying valuable information about solar activity and variability that was not realised at the time of observations. However, many of the early observations were also performed at a time that the observing process was developing, thus they carry significant difficulties in analysing them meaningfully. With the aid of modern analysis techniques, these issues affecting legacy datasets can be overcome and the archives revisited and repurposed to improve our understanding of solar activity and variability well beyond the era of contemporary instrumentation. Thus, historical solar observations are crucial for addressing key questions about the origin and variability of the solar cycle, as well as potential long-term changes in solar irradiance that may influence Earth’s climate. Furthermore, preservation is a critical issue with such historical archives, since not all archives have been made available in digital format yet, while the whereabouts of many historical archives is currently unknown. Unfortunately, the ageing of historical data and potentially not proper storing conditions increase the chance of deterioration and thus the loss of important data.

Actually, the use of historical solar observations to investigate long-term solar variability has a long and well-established tradition, with Wolf (1850) among the earliest studies that systematically employed archival sunspot records in the production of the sunspot number series. Over the years, several reviews have documented and synthesised the progress in recovering and analysing historical sources of sunspot observations at different stages of the field’s development (e.g. Vaquero 2007; Arlt and Vaquero 2020). These works helped stimulate further advances, including major revisions of the sunspot number series and extensive efforts to recover, digitise, and evaluate additional historical archives. However, over the past decades, increasing attention has been given to a broader range of historical solar observations. In particular, spectroscopic records such as Ca ii K and Hα observations, among others, have been recognised as valuable complementary proxies of solar magnetic activity.

**Fig. 1:**
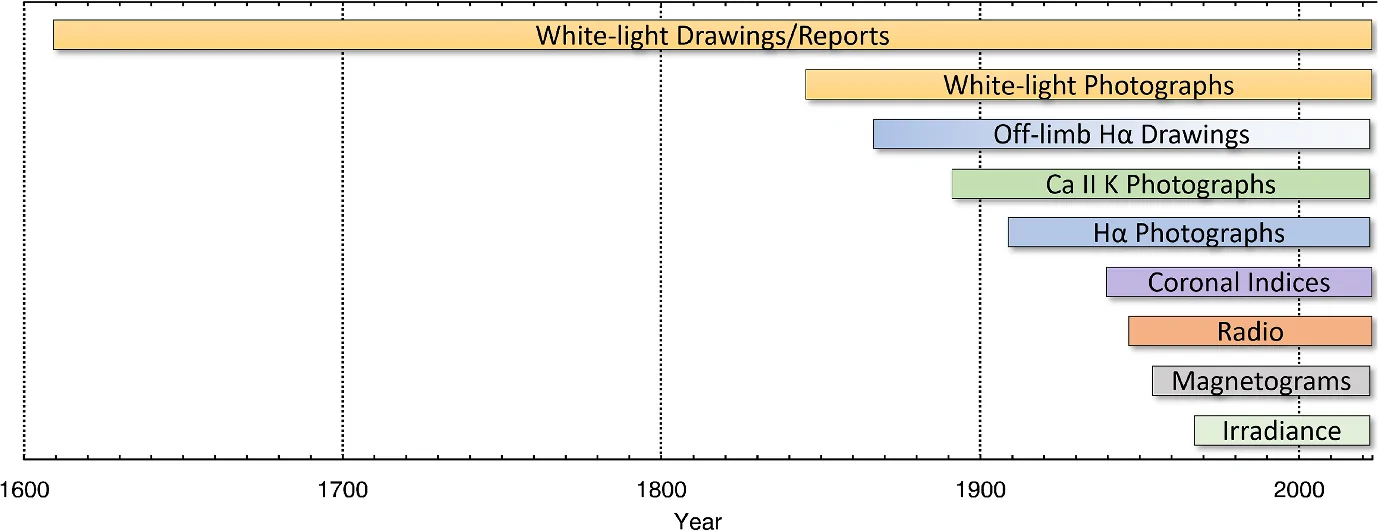
Timeline of the most prominent types of existing long-term archives of solar observations

Here, I will review some of the key historical records, not limited to sunspots, that have contributed to our understanding of solar activity and variability. For the context of this paper, historical will be considered data that cover about 50 years and longer. An emphasis will be given to long-term archives that carry information about the whole solar disc, but off-limb observations as well as some long-term disc-integrated indices will also be discussed. Although sunspot observations will also be discussed, they will not be treated in the same level of detail as in the dedicated review by Arlt and Vaquero (2020); instead, the discussion here is intended to be complementary. Fig. 1 gives a timeline of the types of existing long-term archives of solar observations which will be discussed here. Compiling an overview of these records is essential, not only because they offer valuable insights about solar activity and variability, but also for understanding the associated uncertainties in analyses with these data. Furthermore, it is crucial to have as complete records of the available archives so that hopefully will be a driver for locating and preserving the archives by digitising them and making them available to the community.

The paper is structured as follows. Section 2 gives an overview of the available historical solar observations. Section 3 includes various solar activity metrics that have been directly derived from historical solar observations, while Sect. 4 includes reconstructions, whether of activity metrics or image-based data, with such historical data. The summary and conclusions are presented in Sect. 5. Appendix A gives a comprehensive list of the abbreviations used in this paper.

## Spatially resolved observations

In this section, I provide a brief overview of historical solar observations, organised by the atmospheric layers they probe: the photosphere (Sect. 2.2), chromosphere (Sect. 2.3), and corona (Sect. 2.4). A special emphasis is placed on compilations of multi-wavelength solar observations (Sect. 2.5). To place these datasets in context, I also briefly review the basic principles of the instrumentation underlying such historical solar observations (Sect. 2.1) and the main processing steps used to analyse full-disc observations (Sect. 2.6).

### Basic instruments

The instrumentation used for solar observations has evolved continually, enabling different types of measurements to be made over time (e.g. Stix 2004). Keeping track of all available instruments and improvements made to them over the years is beyond the scope of this paper. In this section, I will very briefly outline the basic principles of the main instruments employed in historical solar observations that will be discussed later. The focus is on instruments that determine the wavelength of observation and on the methods by which the observations were recorded. This overview is provided to clarify important differences among datasets that arise from variations in instrumentation. It should be noted that the vast majority of solar observations covered in the following sections were made with a telescope, which is not discussed here.

The use of a prism to disperse sunlight led to the identification of numerous lines in the solar spectrum in the early 1800 s, marking the beginning of solar spectroscopy. However, isolating specific wavelengths, essential for probing different layers of the solar atmosphere, required further refinement of this basic principle. More specialized instruments enabling such observations began to appear from the late 1860 s onward. Notable developments include the prominence spectroscope (late 1860 s), the spectroheliograph (SHG; 1890 s), and narrow-band optical filters (1940 s). A brief description of these instruments is provided below.

#### Spectroscope (SHS)

**Fig. 2:**
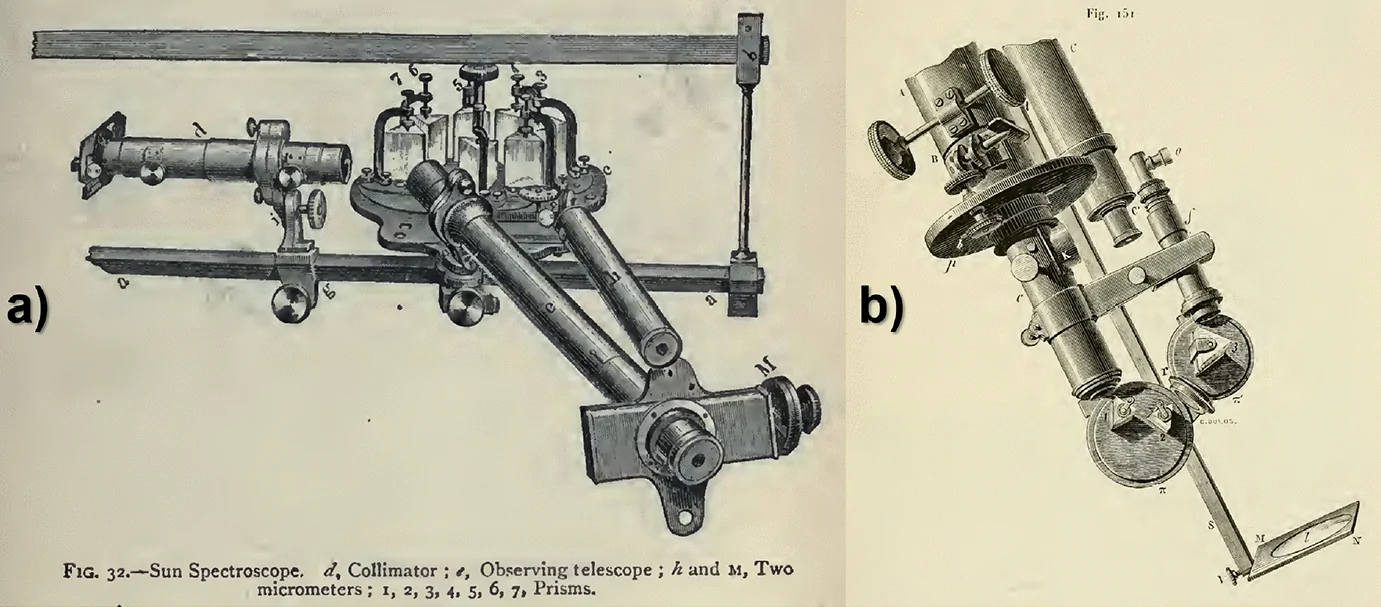
Spectroscopes used by Norman Lockyer (**a**; Lockyer 1873) and Angelo Secchi (**b**; Secchi 1875)

Prominence spectroscope (sometimes also referred to as telespectroscope, because it is attached to a telescope; Chinnici and Consolmagno 2021) is a device that combines a prism (or a series of prisms) with a narrow slit to isolate a thin strip of the solar image (Chinnici and Consolmagno 2021; Pigatto and Zanini 2001). Figure 2 shows two schematics of the spectroscopes used by Lockyer (1873) and Secchi (1875). The prominence spectroscope was used for direct visual observations, typically with the use of some tinted glass to reduce the brightness (Secchi 1875). It featured an adjustable eyepiece, allowing the observer to control its angle and thereby effectively to select the wavelength for observation (Pigatto and Zanini 2001). However, because the spectroscope could not effectively filter out unwanted wavelengths, the intense brightness of the solar photosphere overwhelmed the much fainter emissions from the other atmospheric layers. As a result, spectroscopes could effectively reveal chromospheric features only outside of the solar disc, where the intense brightness of the photosphere was absent. Consequently, early observations with a spectroscope were limited to studying chromospheric prominences at the solar limb (Chinnici and Consolmagno 2021), hence their name as prominence spectroscopes. Leading solar physicists of the era, such as Angelo Secchi, Pietro Tacchini, and Lorenzo Respighi, quickly adopted this technique and performed some of the earliest surviving monochromatic observations of the Sun. The early observations with a prominence spectroscope appear to have been performed in the He i D$_3$ line at 5876 Å and Hα line (Chinnici and Consolmagno 2021; Foukal 2008). To my knowledge, no such photographic data exist, only drawings. Historical solar observations performed with a prominence spectroscope are discussed in Sect. 2.3.2, while solar activity metrics derived from them are discussed in Sect. 3.6.

Hale (1929) mentions that Janssen had introduced a second slit in his spectroscope, effectively applying the same principle as of the SHG, but this was not used for many years. Adjustments by Hale (1924, 1929) made observations of the solar disc with a spectrohelioscope (SHS) possible. Thus, the SHS allowed for fast visual observations of the solar disc in different wavelengths, important for detecting flares. Some known historical solar observations performed with SHS are discussed in Sect. 2.3.2, while solar activity metrics derived from them are discussed in Sect. 3.6 and 3.7.

#### Spectroheliograph (SHG)

**Fig. 3:**
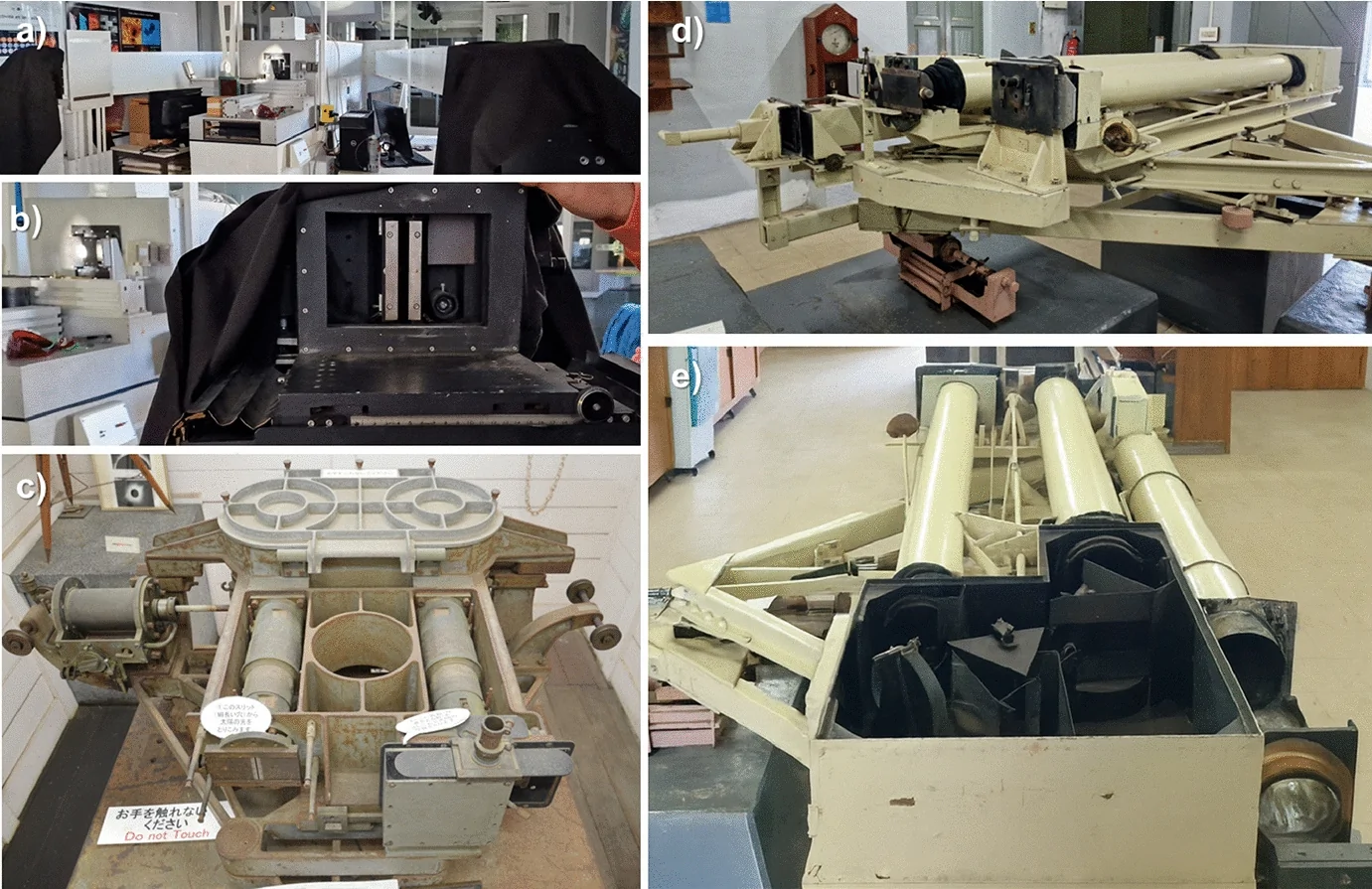
Photographs of three prominent historical spectroheliographs: Meudon (panels **a** and **b**; with panel b highlighting the exit slit), Kyoto (panel **c**), and Kodaikanal (panels **d** and **e**)

A transformative development came in the early 1890 s with George Ellery Hale’s invention of the SHG (Hale 1891, 1904). This made it possible to photograph the full solar disc in narrow spectral bands and thus to acquire on-disc photographs of different layers of the Sun’s atmosphere. Figure 3 shows photographs of three of the most prominent SHGs.

Hale and Ellerman (1903) writes “The principle of the spectroheliograph is exceedingly simple. Imagine a direct-vision spectroscope in which the eyepiece ordinarily employed is replaced by a (second) slit”. The basic principle of the SHG is a narrow slit at the entrance, allowing only a narrow strip of the solar image to go through the instrument, followed with a dispersion system, whether using prisms or a grating system, and at the end there is another narrow slit (see Fig. 3b), which now controls the wavelength range that will be kept. Thus, in contrast to the spectroscope, the SHG is able to filter out unwanted wavelengths. The SHG is not used for direct visual observation; instead, a photographic camera or plate is positioned at the end of the system, allowing the whole solar disc to be photographed in successive narrow strips. The duration needed to take a full image of the Sun depends on the details of the system, but typically requires a few minutes.

Overall, the SHG was a complicated system that required even motion of the different parts of the instrument during the observation. Unfortunately, this was not always the case, which led to various images where the disc appear elongated or subsequent strips extended over others leading to repeated and overlapping exposures. Characteristic artefacts of these are brightness variations across linear strips from different exposures. Issues with the motion of the instrument also resulted in increased ellipticity in the recorded solar disc, which has been reported to typically range between 0.1 and 0.2, with isolated extreme values reaching 0.8 (Chatzistergos et al. 2020b, 2023a). The way the SHG is built it allows to change the width of the slits and thus change the passband. There are also a few cases where the passband or central wavelength of the observation changed during the scanning (See an example in Fig. 4 in Chatzistergos et al. 2018b), it is unclear if that was intentional or due to issues of the instrument. Initial SHG observations focused on the Ca ii K line (Sect. 2.3.1), the strongest absorption line in the visible solar spectrum (Hale 1892b), and Hα (Sect. 2.3.2), but observations in other lines also exist (Sect. 2.2.2, 2.3.3, 2.4.2, and 3.9). Solar activity metrics derived with spectroheliograms are discussed in Sect. 3.3–3.6.

#### Filters

Optical filters enable real-time imaging of the full solar disc by discarding unwanted wavelengths and isolating specific portions of the spectrum (e.g. Stix 2004). Achieving broad-band transmission, of roughly 1000 Å, typically involves the use of coloured glass plates. Narrow-band filtering was made possible after the invention of the monochromatic filter by Lyot (1944). Narrow-band historical observations were performed primarily through two types of devices: interference filters and birefringent Lyot filters (Lyot 1944). Interference filters consist of multiple thin dielectric layers with alternating high and low refractive indices, producing transmission windows typically wider than 1 Å. In contrast, Lyot filters achieve narrower passbands and are constructed from alternating layers of polarisers and uniaxial birefringent crystals.

Because filters allow the entire solar disc to be photographed instantly, they enabled high-cadence imaging, something that was not possible earlier with the SHG (due to the long time needed for the observation). Filters are placed directly in the optical path of the telescope, enabling a significantly simpler instrument setup compared to SHGs. Consequently, filtergrams generally avoid most of the image distortions and artefacts seen in spectroheliograms, such brightness variations along linear image segments. Similarly, filtergrams typically produce solar disc images with lower ellipticity than spectroheliograms, with typical ellipticity values of around 0.05 (Chatzistergos et al. 2020b). However, factors such as filter tilt can still affect the effective bandpass (e.g., Löfdahl et al. 2011). Furthermore, the filter transmission is affected by observation conditions (e.g. temperature), while filter degradation over time can alter transmission characteristics. Historical solar observations performed with a filter are discussed in Sect. 2.2 and 2.3, while solar activity metrics derived from them are discussed in Sect. 3.1–3.7.

#### Recording methods

**Fig. 4:**
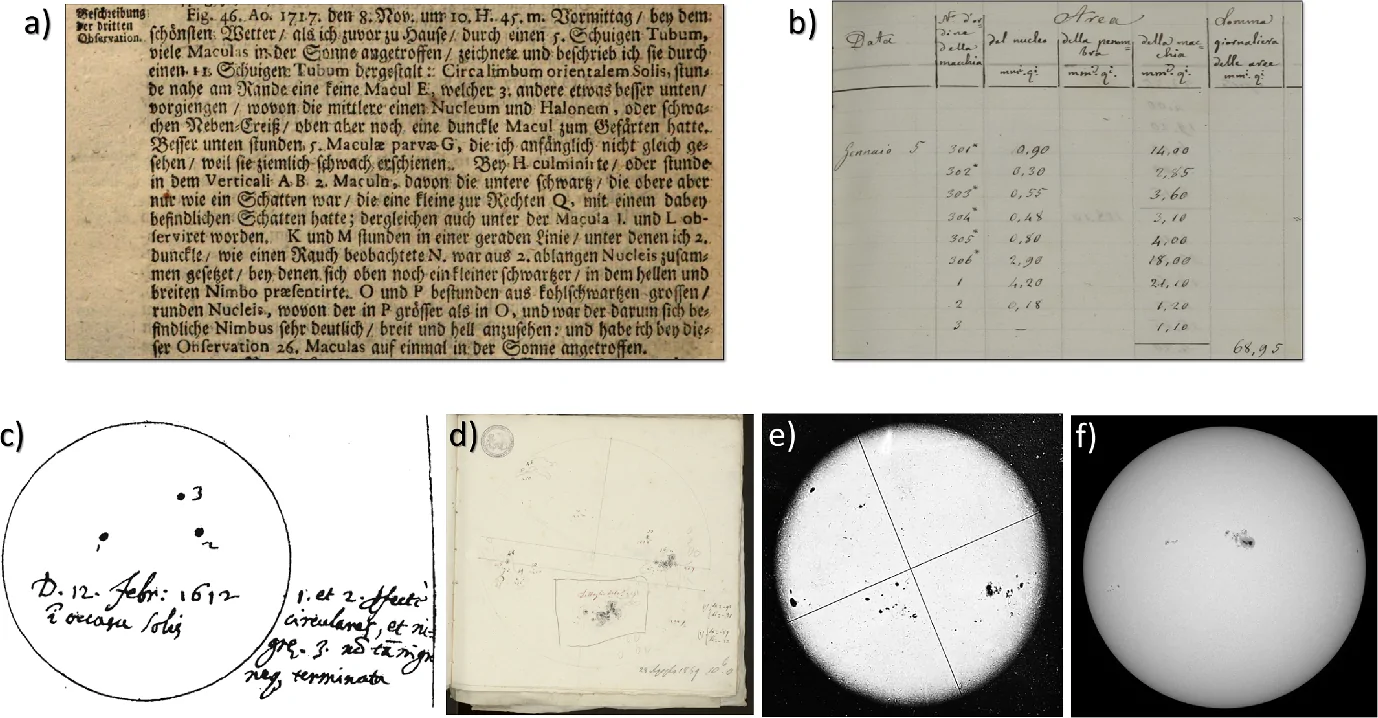
Examples of the different types of extant white-light and continuum solar observations. The cases shown are: **a** Textual description of observation by Johann Leonard Rost from 8 November 1717 (Rost 1718), **b** Table of individual group areas by Angelo Secchi from 5 January 1871 (Ermolli et al. 2023), **c** Full-disc drawing by Galileo from 12 February 1612 (Galilei 1895), **d** Full-disc drawing by Secchi from 28 August 1859, **e** Full-disc analogue photograph from Meudon taken on 21 September 1870, and **f** Full-disc digital photograph by SDO/HMI taken on 5 May 2025

The available spatially resolved historical data were either photographic or visual. Visual observations survive in the form of drawings, tabulations of some solar feature characteristics, or simply as textual descriptions. All observations prior to 1845 are in this form, but such observations continue to be made to this day. Historical records in the form of textual descriptions or tabulations (Fig. 4a and b), although valuable, are among the most limited sources of information, as they capture only what was explicitly noted by the observers. Given the less advanced instrumentation available prior to the 1800 s and the limited understanding of solar physics at the time, this represents a noteworthy limitation that requires consideration when using such data. Drawings (Fig. 4c and d) provide more information, while they also allow for potential reanalyses. The information that is included in the drawings, however, follows the advance in instrumentation (Sect. 2.1) and understanding of solar physics (see Sect. 2.2–2.4). Many drawings were made by projecting the solar image onto paper via the telescope, helping to preserve the relative positions and sizes of solar features, but many drawings are rough sketches by direct visual observation, resulting in more qualitative and less spatially accurate depictions of features. Even drawings that are performed by projecting the solar disc on a piece of paper are affected by small positional shifts which occur over the few minutes needed to complete the drawing. Drawings that were performed with overlays or projections of actual photographic observations do not have this issue. Drawings have further limitations due to the detail and accuracy of the drawings which depend on the observer’s skill, intent, and available instrumentation (acuity level, the smallest feature they can resolve). For instance, panels c and d in Fig. 4 give an example of the different levels of detail included in such drawings, with the first drawing showing only big spots as rough circles while the second image includes details of the spots, even an enlargement of one group, as well as coarse depictions of faculae. Another limitation of drawings is that they provide information on the locations and shapes of features, but not on their brightness. However, an important advantage of drawings is that observers could compensate for poor seeing by integrating impressions over time.

Photographs provide a more objective way to record the observations, while also carrying more information than the drawings, for instance including information about the features’ brightness. Since they are momentary snapshots of the Sun, they can provide more accurate information about the position and size of the features of interest. Ground-based photographic observations are, however, more vulnerable to atmospheric conditions than drawings. Historical analogue photographs have additional complications due to the photographic process. That is foremost due to the nonlinearity in the photographic material’s response to incident light (see Sect. 2.6.2). Furthermore, the ageing of the photographs (and introduction and accumulation of artefacts over the years) as well as changing quality due to the evolving practices in photography are added complications. Analogue photographs exist since 1845 while to my knowledge the last such analogue photograph for solar observation was performed in 2010. Digital photographs have been acquired since at least 1974. Various devices have been used for this, mostly a charge-coupled device (CCD; Boyle and Smith 1970), complementary metal oxide semiconductor (CMOS; Meynants et al. 1998) cameras, but earlier data were also acquired with linear diode arrays (e.g. Choisser et al. 1974) or digital video recorders (e.g. Eto et al. 1987). The resolution and quality of digital images has improved significantly over the years.

**Table 1:** List of photographic white-light datasets. Columns are: name of the observatory, approximate period of observations listed separately for analogue and digital data, whether the analogue data have been digitised (Y for digitised, N for not digitised or unknown, P for only partly digitised, and D if the data are originally only in digital format), and the bibliography entry

Observatory	Period		Dig	Reference
	Analogue	Digital		
Abastumani	1950–1990	–	N	Khetsuriani (1967)
Athens	1967–1978	–	N	Macris (1967)
Belgrade	1941–1959	–	P	Wöhl (2005)
Big Bear	–	1987–2005	D	Denker et al. (1999)
Boulder	1973–1974	–	Y	^1^
Brussels	–	2006–	D	^2^
Cape of Good Hope	1876–1976	–	N	Warner (1979)
Catania	1963–1998	–	N	Zuccarello et al. (2011)
Debrecen	1958–2016	–	Y	Baranyi et al. (2016)
Dehra Dun	1878–1925	–	N	^3^
Ebro	1905–2000	2000–	N	Curto et al. (2016)
Göttingen	1940–1944	–	P	Wöhl (2005)
Guyla	1972–2016	-	Y	Baranyi et al. (2016)
Harvard College	1870–1876	–	N	Harvard College Observatory (1876)
Helios	–	2012–	D	^4^
Helwan	1957–1996	–	N	^5^
Herstmonceux	1949–1976	–	Y	Jones (1950)
Kanzelhöhe	1989–2007	2007–	Y	Pötzi et al. (2016)
Kew	1858–1872	–	N	de La Rue et al. (1869)
Kiev	1916–1989	–	N	Yakovkin (1972)
Kislovodsk	1948–2010	2010–	Y	Tlatov et al. (2019)
Kodaikanal	1904–2017	–	Y	Jha et al. (2022)
Kodaikanal Twin	–	2008–2013	D	Singh and Ravindra (2012)
Kodaikanal WARM	–	2015–	D	Pruthvi (2015)
Locarno	1957–1983	–	Y	Waldmeier (1968)
Mauritius	1878–1908	–	N	Willis et al. (2013)
Melbourne	1874–1914	–	N	Clark and Orchiston (2004)
Meudon	–	2003–	D	^6^
Mitaka	1918–1998	1998–	Y	Hanaoka et al. (2016)
Mount Stromlo	1910–1960	–	N	^7^
Mount Wilson	1906–2010	–	Y	Howard et al. (1984)
Ondřejov	1944–2006	2006–	N	Švestka (1970)
Potsdam	1943–1991	–	Y	Pal et al. (2020)
RGO	1874–1949	–	P	Willis et al. (2013)
Schauinsland	1947–1953	–	P	Wöhl (2005)
Shamakhy	1957–	–	N	Musayev and Seyidov (2010)
South Kensington	1879–1913	–	N	Lockyer (1913)
Syracuse	1942–1943	–	P	Wöhl (2005); Seiler (2007)
THEMIS	–	2019–	D	^8^
Ulugh Beg	1952–1996	-	N	Scheglov and Slonim (1970)
Upice		1998–	N	Klimeš et al. (1999)
US Naval	1923–1951	–	P	Peters and Wagman (1930)
Ussuriysk	1954–2005	2005–	N	^9^
Valašské Meziříčí	1957–2012	–	Y	Lenza et al. (2014)
Vilnius	1865–1876	–	Y	Vázquez et al. (2026)
Wendelstein	1944–1977	–	P	Wöhl (2005)
Yerkes	1902–1938	–	N	Hale (1902)
Yunnan	1939–2011	2011–	N	Zhang et al. (1993)
Zürich	1957–1980	–	N	Waldmeier (1968)

^1^ = https://www.ngdc.noaa.gov/stp/space-weather/solar-data/solar-imagery/photosphere/white-light/boulder/

^2^ = https://www.sidc.be/uset/telescopewl.php

^3^ = https://archive.fo/uMqeH

^4^ = https://cesar.esa.int/index.php?Section=Observatories_ESAC_Sun

^5^ = https://web.archive.org/web/20250803020849/https://whc.unesco.org/en/tentativelists/5574/

^6^ = https://bass2000.obspm.fr/instru_guide.php#9

^7^ = https://web.archive.org/web/20241203013324/https://www.eoas.info/biogs/A002233b.htm

^8^ = https://www.themis.iac.es/doku.php?id=observation:data

^9^ = https://astrogalaxy.ru/514.html

**Table 2:** List of photographic narrow-band photospheric datasets. Columns are: name of the observatory, type of instrument, period of observations, central wavelength, passband (SW, for spectral width), and the bibliography entry

Observatory	Instr	Period	Wavelength [Å]		SW [Å]	Reference
Aditya-L1 SUIT	Filter	2023–	UV	2767	4	Tripathi et al. (2025)
Aditya-L1 SUIT	Filter	2023–	UV	2832	4	Tripathi et al. (2025)
Aditya-L1 SUIT	Filter	2023–	UV	3000	10	Tripathi et al. (2025)
CHASE	Filter	2021–	Fe i	6569	0.14	Li et al. (2019)
GONG Big Bear	Filter	1995–	Ni i	6768	1	Harvey et al. (1996)
GONG Cerro Tololo	Filter	1995–	Ni i	6768	1	Harvey et al. (1996)
GONG El Teide	Filter	1995–	Ni i	6768	1	Harvey et al. (1996)
GONG Learmonth	Filter	1995–	Ni i	6768	1	Harvey et al. (1996)
GONG Mauna Loa	Filter	1995–	Ni i	6768	1	Harvey et al. (1996)
GONG Udaipur	Filter	1995–	Ni i	6768	1	Harvey et al. (1996)
Hida SMART	Filter	2003–	Fe i	6303	0.1	Ueno et al. (2004)
Huairu SMAT	Filter	2005–	Fe i	5324	0.1	Zhang et al. (2007)
ISOON	Filter	2003–2012	Fe i	6303	0.1	Balasubramaniam and Henry (2016)
KP/512	Filter	1974–1993	Fe i	8688	–	^1^
KP/SPM	Filter	1992	Fe i	5507	–	^1^
KP/SPM	Filter	1992–2003	Fe i	8688	–	^1^
Kodaikanal WARM	Filter	2015–	G-Band	4305	8.4	Pruthvi (2015)
Meudon	SHG	1909, 1927	Fe i	4384	–	D’azambuja (1930)
Meudon	SHG	1927	Sr ii	4078	–	D’azambuja (1930)
Meudon	SHG	1927	Ca i	4227	–	D’azambuja (1930)
Meudon	Filter	2008–2009	Green	5302	5	^2^
Meudon	Filter	2009–2014	G-Band	4300	8	^2^
Mitaka	Filter	2012–2022	G-Band	4305	10	Hanaoka et al. (2016)
MLSO/PSPT	Filter	1998–2015	Red	6071	4.6	Rast et al. (2008)
MLSO/PSPT	Filter	1998–2015	Blue	4094	2.7	Rast et al. (2008)
Picard/SODISM	Filter	2010–2014	Green	5357	5	Meftah et al. (2014)
Picard/SODISM	Filter	2010–2014	Red	6071	7	Meftah et al. (2014)
Picard/SODISM	Filter	2010–2014	near IR	7822	16	Meftah et al. (2014)
Rome/PSPT	Filter	1997–	Red	6072	5	Ermolli et al. (2022a)
Rome/PSPT	Filter	1996–	Blue	4094	2.5	Ermolli et al. (2022a)
Rome/PSPT	Filter	2007–2008	Green	5357	5	Ermolli et al. (2022a)
Rome/PSPT	Filter	2007–	G-Band	4306	12	Ermolli et al. (2022a)
SDO/HMI	Filter	2010–	Fe i	6173	0.08	Schou et al. (2012)
SO/PHI	Filter	2021–	Fe i	6173	0.1	Solanki et al. (2020)
SoHO/MDI	Filter	1996–2011	Ni i	6768	0.09	Scherrer et al. (1995)
Yerkes	SHG	1903	Fe i	4384	–	Hale and Ellerman (1903)
Yerkes	SHG	1903	Ca i	4227	–	Hale and Ellerman (1903)

^1^ = https://nso.edu/data/historical-archive/

^2^ = https://bass2000.obspm.fr/instru_guide.php#6

### Photospheric observations

In this section I will give an overview of the various historical archives of photospheric observations. These include white-light (Sect. 2.2.1) and narrow-band observations (Sect. 2.2.2) as well as solar surface magnetic field data (Sect. 2.2.3).

#### White-light observations

The term white-light typically refers to solar observations made over a broad wavelength range that covers most or all of the visible spectrum. This includes both observations without any specific spectral filtering and those using broad-bandpass filters. For example, the Kanzelhöhe, Kodaikanal WARM (White-light Active Region Monitor; Pruthvi 2015) and Twin, and San Fernando (with two Cartesian Full Disk Telescopes, CFDT) observatories acquire white-light images with a filter with a nominal bandpass of 100 Å (Pötzi et al. 2021; Chapman et al. 2013), Mitaka of 500 Å (Hanaoka et al. 2016), while Brussels observatory uses a filter with an even broader nominal bandpass of 1580 Å.^1^

Because observing the Sun in white-light does not necessarily require specialized equipment, it can even be done with the naked eye. However, direct observation of the Sun is strongly discouraged, as it can cause serious and permanent eye damage. In fact, people have observed the Sun with the naked eye since the antiquity, sometimes using aids such as tinted glass or by viewing it through fog or thin clouds. Reports of dark features on the solar disc, likely sunspots, exist at least as early as the 4th century BCE (e.g. Hardy 1991; Yau and Stephenson 1988; Vaquero et al. 2002a). These accounts are often vague, comparing the sunspots to familiar objects like fruits, eggs, or birds to roughly indicate their apparent size. A few historical drawings from naked-eye observations also survive (Arlt and Vaquero 2020). Nevertheless, such records are sporadic, only capture the largest sunspot groups, and are influenced by non-astronomical factors such as political, social, or cultural conditions. These limit the usefulness of naked-eye solar observations for systematic studies of solar activity.

There are some sporadic observations performed with a camera obscura from at least the 1500 s until the mid 1700 s (Arlt and Vaquero 2020). Possibly the earliest surviving drawing of a solar observation with a camera obscura was done by Frisius et al. (1545) during the 1544 eclipse. This drawing however does not show any sunspot, which is likely due to the low activity during the Spörer minimum, but it cannot be ruled out that solar features were seen but not drawn. An early surviving solar observation with a camera obscura was performed by Kepler in 1607 (Vaquero 2007). Another important source of camera obscura solar observations comes from the cathedral of San Petronio in Bologna, where records were maintained for the period 1655–1736 (Manfredi 1736). Observations with camera obscura eventually ceased due to the preference of telescope.

The invention of the telescope in the early 1600 s marked the beginning of systematic white-light observations of the Sun. These observations were initially made visually, surviving to us either in the form of tabulations (Fig. 4b), textual descriptions (Fig. 4a), or drawings (Fig. 4c–d). Since the mid 1800 s, analogue photographs (Fig. 4e) have also complemented these records, and more recently digital images (Fig. 4f). Figure, 4 provides some examples of the various types of white-light observations that exist. Overall, there have been more than 700 professional and amateur observers performing white-light solar observations since the 1600 s (Vaquero et al. 2016; Arlt and Vaquero 2020).

The very first photograph of the solar surface in white-light was taken by Louis Fizeau and Léon Foucault in 1845.^2^ However, it would appear that there were no systematic photographic white-light observations before those from Kew observatory started in 1858. The Kew photoheliograph was to my knowledge the first instrument built specifically for performing systematic solar photography. Observations at Kew continued until 1872, while the photoheliograph was moved to Royal Greenwich Observatory (RGO) for operation between 1873 and 1875 (Willis et al. 2016). Since then, there are continuous white-light observations of the Sun from various sites around the globe. Table 1 gives some information about some of the white-light photographic archives. I note that the dates of observations for Mount Stromlo in Table 1 are very approximate, while most of their plates unfortunately did not survive the 2003 Canberra bushfires. Although Table 1 is likely incomplete and may contain inadvertent errors, it nevertheless highlights the wealth of available photographic white-light data.

RGO is a particularly important archive of white-light photographic observations which covers the period 17 April 1874 to 31 December 1976. Although only the observations until 2 May 1949 were performed in Greenwich, while the later ones were performed from Herstmonceux. Various instrumental changes took place over the years. Unfortunately, RGO photographic plates before 1918 are thought to have been destroyed in the First World War, with only some photographic prints from that period existing (Dezsö 1987).

Many efforts have led to the digitisation of many of the historical archives (see Table 1), such as the ones from Mitaka, Potsdam, and Kodaikanal. Wöhl (2005) in particular, digitised small samples of white-light observations from observations sent to Kiepenheuer-Institut für Sonnenphysik from Wendelstein, Schauinsland, Göttingen, Belgrade, and Syracure sites. Kew observatory and RGO photographs to my knowledge are not currently available in digital format. Kodaikanal is another important white-light archive, with observations having started in 1904 (Bappu 1967) continuing to this day, while digital images started being acquired since 2008. All surviving Kodaikanal plates have been recently digitised (Ravindra et al. 2013). Most historical observatories have stopped performing white-light observations; to my knowledge, only those at Ebro, Kanzelhöhe, Kislovodsk, Mitaka, and Ussuriysk continue to this date, albeit with instrumental updates.

#### Narrow-band Photospheric observations

Solar narrow-band photospheric observations have been made intermittently using various spectral lines or continuum wavelength intervals over the past century. To my knowledge, only sporadic such observations were performed with a SHG in the beginning of the 1900 s. In particular, SHG observations in the Fe i 4384 Å line were carried out at Yerkes in 1903 (Hale and Ellerman 1903) and at Meudon in 1909 (D’azambuja 1930), while sporadic observations in the Sr ii 4078 Å and Ca i 4227 Å lines were obtained at Meudon in 1927 (Hale and Ellerman 1903; D’azambuja 1930). It is unclear if more such narrow-band photospheric SHG observations were performed and whether they survive. Systematic digital narrow-band photospheric observations using filters started in the 1970 s. Table 2 lists some of these narrow-band photospheric observations along with some basic information about them. To my knowledge, the earliest such data were performed at Kitt Peak, where regular observations in the Fe i 8688 Å line were carried out from 1974 to 2003. The Rome Precision Solar Photometric Telescopes (PSPT), Mauna Loa solar observatory (MLSO) PSPT, Mitaka, Kodaikanal WARM (Pruthvi 2015), and the Improved Solar Observing Optical Network (ISOON; Neidig et al. 1998) have also contributed valuable observations in different continuum intervals. The Global Oscillation Network Group (GONG) of observatories (Harvey et al. 1996; Jain et al. 2026), including Learmonth, Udaipur, El Teide, Cerro Tololo, Big Bear, Mauna Loa, and the engineering site in Boulder, perform continuum observations at the Ni i 6768 Å line since 1995.

More recently, space-based instruments such as SoHO/MDI (1996–2011), the Helioseismic and Magnetic Imager (HMI; Scherrer et al. 2012) onboard SDO (since 2010), Solar Diameter Imager and Surface Mapper (SODISM) onboard Picard (Meftah et al. 2014, covering 2010–2014), and the Chinese Hα Solar Explorer (CHASE; Li et al. 2022 since2021) have provided data in the Ni i 6768 Å line, Fe i 6173 Å line, three different continuum intervals, and Fe i 6569 Å respectively. Finally, the Polarimetric and Helioseismic Imager (PHI; Solanki et al. 2020) on board SO has been acquiring Fe i 6173 Å line filtergrams since 2021 from locations outside the Sun–Earth line and at different inclination angles, which is planned to reach up to approximately 33$^\circ$, thereby allowing improved observations of the solar poles.

#### Solar surface magnetic field records

**Fig. 5:**
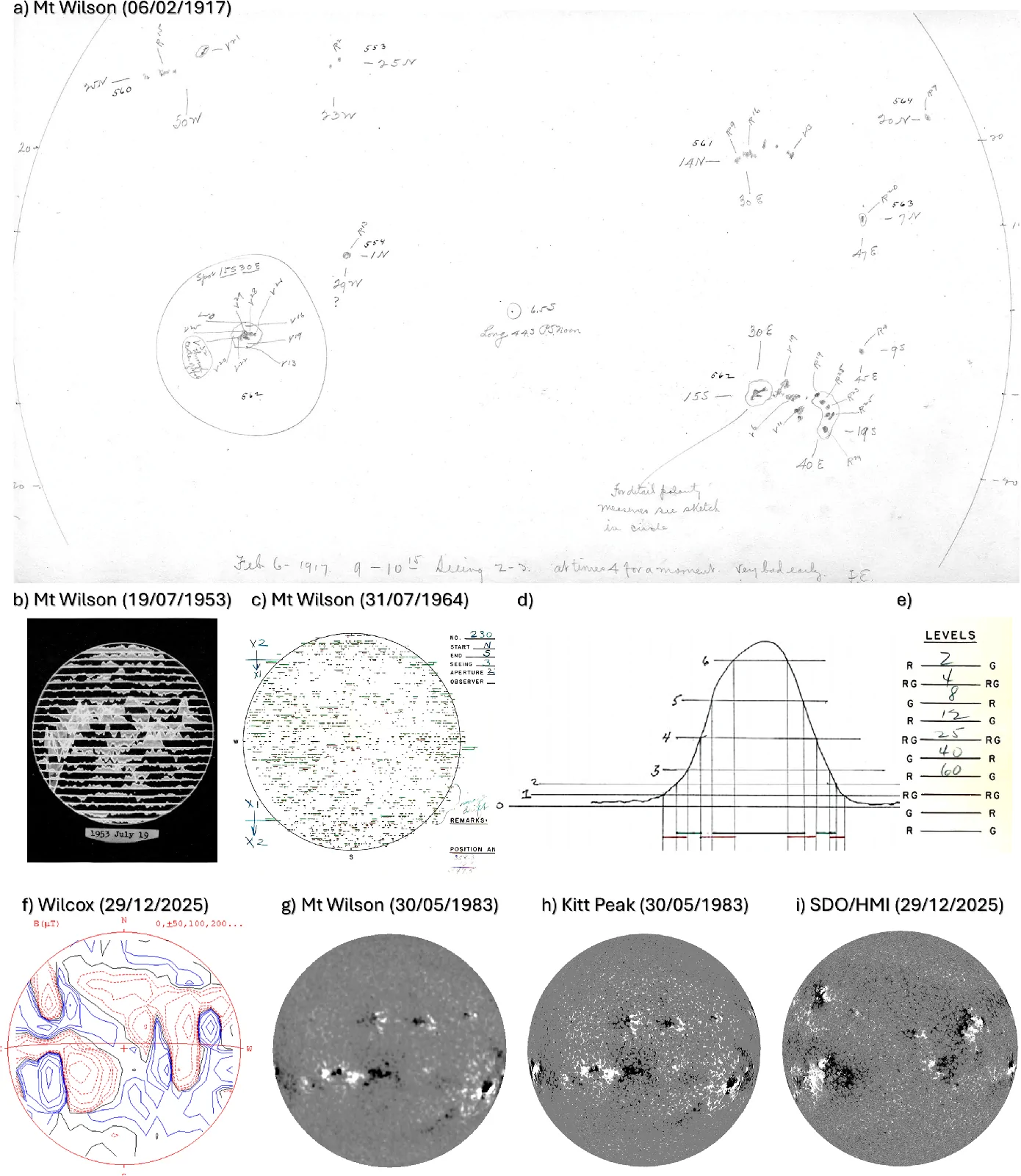
Examples of solar surface magnetograms. Panel (**a**) shows a sunspot drawing including their magnetic field strengths (in units of 100 G) and their polarities (“R”, red, for positive and “V”, violet, for negative) (Courtesy of R. Ulrich). Panel (**b**) shows the first generation of Mount Wilson magnetograms (taken from Babcock 1953), while the second generation, “pen plots”, is shown in panel (**c**) along with the level information allowing interpretation of the magnetic fields (Panels **d**–**e**) (taken from Pevtsov et al. 2021). Panel (**f**) shows a Wilcox solar observatory magnetogram with red and blue marking negative and positive polarities (courtesy of J.T. Hoeksema). Panels (**g**–**i**) show line-of-sight magnetograms saturated at ±50 G where positive and negative polarities are shown in white and black, respectively

The spectroscope allowed already in the 1870 s to observe line splitting for various lines when observing sunspots (see e.g. Pevtsov et al. 2021 forareview). However, the first measurement of magnetic field in a sunspot was made by Hale (1908) following the discovery of the Zeeman effect (Zeeman 1897).

Since 1917 Mount Wilson observatory systematically measured the magnetic field and polarity of sunspots (Pevtsov et al. 2019). An example drawing is shown in Fig. 5a). Although measurements were carried out for all observed sunspots, they were relatively coarse, being expressed as single values characterizing each sunspot in its entirety. Potsdam observatory applied a photographic technique for measuring the magnetic field over several locations of a sunspot and thus mapping the magnetic field around a spot for the first time in 1942 (Von Klüber 1948). Unfortunately, these observations did not necessarily include all sunspots present on the solar surface. Such observations continued until the 1970s, when Potsdam started using a photoelectric stokes polarimeter (Staude 1991). Magnetic field measurements in sunspots, similar to those conducted at Mount Wilson, were also carried out since the late 1950s at various sites across the former Soviet Union (Pevtsov et al. 2011, 2016a). In particular, the Crimea (since 1957), Pulkovo (1957–1997), Institute of Terrestrial Magnetism, Ionosphere and Radiowave Propagation (1957–1966), Sayan Solar Observatory of Institute of Solar-Terrestrial Physics (1964–1995), Shamakhy Astrophysical Observatory (1966–1979), Ussuriysk Astrophysical Observatory (1966–1989), and Astronomical Observatory of Ural State University (1967–1979) (Pevtsov et al. 2011). There are, however, some concerns about the consistency of these measurements. In particular, Pevtsov et al. (2021) reported that Mount Wilson measurements of sunspot magnetic fields reached values as low as 100 G until 1978, after which there are only a few measurements below 1 kG. Similar gaps in magnetic field measurements were noted for at least the Crimea, Pulkovo, and Potsdam records.

The first successful measurements of magnetic fields outside sunspots were reported by Thiessen (1946) and Kiepenheuer (1953). Shortly thereafter, the first full-disc maps of the solar surface magnetic field were produced at Mount Wilson in 1953 (Babcock and Babcock 1952; Babcock 1953). These early maps were rather coarse, recording the magnetic field across about 22 linear segments taken at different parts of the disc. This is effectively plotting the magnetic field strength, with values above and below the line for positive and negative polarities. One of the earliest such magnetograms is shown in Fig. 5b. This type of magnetograms were recorded until 1962. Since 1963 a second generation magnetograph was used, which has also been called as “pen-plot” magnetograph (see Fig. 5c–e). That is because the solar image was now scanned in rasters and two separately actuated colour pens wrote the information about the magnetic field strength and polarity as a coded sequence of lines. Figure 5e) shows an example of the corresponding magnetic field strengths (in G) for the different levels, while Fig. 5d illustrates how the colour pens are mapped onto these levels for a representative region. Mount Wilson upgraded to a digital data recording system producing better resolved maps of magnetic field between 1966 and 2013 (Fig. 5g. Over this period a Babcock solar magnetograph was used, recording the Zeeman polarization in the wings of the Fe i 5250 Å line (Ulrich 1992). Various instrumental updates were performed over the years (see e.g.Howard et al. 1983; Ulrich et al. 1991, 2002; Ulrich and Boyden 2013; Ulrich et al. 2024), which unfortunately affect the consistency of the records. A notable update is over 1981 when the magnetograph was rebuilt improving the stability and calibration of the data.

In the following years, several sites began routine acquisition of solar surface magnetograms. Daily line-of-sight (LOS) magnetograms were obtained at Kitt Peak Observatory from spectropolarimetric observations of the Fe i 8688 Å line (Sect. 2.2.2) using a 512-channel Babcock-type magnetograph (KP/512) over the period from 1 February 1974 to 10 April 1993 (Fig. 5h). Kitt Peak observatory also used a CCD spectromagnetograph (KP/SPM) to produce LOS magnetograms over the period from 19 November 1992 to 21 September 2003. Routine low-resolution longitudinal magnetic field measurements have been available from the Wilcox Solar Observatory since 1976 (Scherrer et al. 1977) (Fig. 5f), based on observations of the Fe i 5250 Å line with a Babcock magnetograph (Hoeksema 1984). Further ground-based full-disc longitudinal magnetograms have been obtained since 1995 by the GONG network of observatories (see Sect. 2.2.2) sampling the Ni I 6768 Å line (Leibacher 1999; Harvey et al. 1996), ISOON over 2003–2012 sampling the Fe i 6303 Å line (Keller 2000; Balasubramaniam and Henry 2016), and Huairu over the Fe i 5324 Å line (Deng et al. 1997). While vector magnetograms have been collected since 2003 at Hida observatory with the Solar Magnetic Activity Research Telescope (SMART) sampling the Fe i 6303 Å  line (Ueno et al. 2004), since 2005 at Huairu observatory with the Solar Magnetism and Activity Telescope (SMAT) sampling the Fe i 5324 Å line (Zhang et al. 2007; Li et al. 2025), and between 2003–2017 with the Synoptic Optical Long-term Investigations of the Sun (SOLIS) Vector-Spectromagnetograph (VSM;Keller et al. 2003) sampling the Fe i 6320 Å line. There are also magnetograms of individual active regions. In 1962, Meudon began acquiring magnetograms of individual active regions in the Fe i 6302 and 5250 Å lines (Michard and Rayrole 1965). Three different observational systems were used over the course of the programme in Meudon, the last of which remained in operation until 1985 (Malherbe 2026). The Okayama Observatory acquired maps of the vector magnetic field in active regions over 1982–1995 with the vector magnetograph (Hanaoka et al. 2016). The active regions were scanned in steps of 6–10 arcseconds, with a complete scan of an active region requiring approximately 60–90 min. During 1992–2017, the Solar Flare Telescope at Mitaka Observatory produced vector magnetograms of active regions using a narrow-band filter and a CCD camera, with acquisition times of about one minute per map. High-resolution photospheric magnetic field measurements over a small field of view can also be derived from observations acquired with the CRisp Imaging SPectropolarimeter (CRISP; Scharmer et al. 2008 since2008), Interferometric BIdimensional Spectrometer (IBIS; Cavallini 2006; Ermolli et al. 2022b covering2012–2019), and Telescope Héliographique pour l’ Etude du Magnétisme et des Instabilités Solaires (THEMIS; Schmieder et al. 2025 since1998).

Over the recent decades continuous space-based observations were also made possible. Space-based measurements were provided by SoHO/MDI, which produced full-disc longitudinal magnetograms from filtergrams sampled at four wavelength positions across the Ni i 6768 Å line. SoHO/MDI operated from 19 March 1996 to 11 April 2011. Its successor, SDO/HMI, has provided full-disc longitudinal and vector magnetograms (Fig. 5i) since 30 April 2010, with cadences of 45 s and 720 s, respectively, sampling six wavelength positions across the Fe i 6173 Å line, and continues to operate to the present day. Finally, since 2021 SO/PHI (Solanki et al. 2020) produces full-disc longitudinal magnetograms by sampling the Fe i 6173 Å line.

### Chromospheric observations

In this section I will give an overview of the various historical archives of chromospheric observations. These include Ca ii K (Sect. 2.3.1), Hα (Sect. 2.3.2) as well as Ca ii H , Ca ii infrared, Hβ, Hγ, and Mg ii h and k (Sect. 2.3.3) observations.

**Table 3:** List of photographic Ca ii K datasets. Columns are: name of the observatory, type of instrument, approximate period of observations, passband, cadence (H for high and L for low), whether the data have been digitised (Y for yes, N for no or unknown, and P for partly digitised), and bibliography entry

Observatory	Instrument	Period	SW [Å]	Cad	Dig	Ref
Abastumani	SHG	1957–1986	–	L	N	Khetsuriani (1967)
Anacapri	Filter	1968–1973	2	L	N	Öhman (1968)
Arcetri	SHG	1931–1974	0.3	L	Y	Ermolli et al. (2009a)
Baikal	Filter	1995–2002	0.6	H	N	^1^
Big Bear	Filter	1981–1995	1.5, 3.2	L	P	Marquette (1992)
Cambridge	SHG	1913–1942	–	L	N	Newall (1914)
Catania	SHG	1963–1977	–	L	P	Zuccarello et al. (2011)
Coimbra	SHG	1925–2007	0.16	L	Y	Garcia et al. (2011)
Crimea	SHG	1955–1979	–	–	N	^2^
Ebro	SHG	1905–1937	–	L	N	Curto et al. (2016)
Hamburg	SHG	1943–1945	–	L	N	Wöhl (2005)
Kandilli	Filter	1968–1979	0.3	H	P	Dizer (1968)
Kenwood	SHG	1892–1895	–	L	P	Hale (1893b)
Kharkiv	SHG	1951–1993	3	L	P	Belkina et al. (1996)
Kislovodsk	SHG	1958–2002	–	L	Y	Tlatov et al. (2015)
Kodaikanal	SHG	1904–2007	0.5	L	Y	Priyal et al. (2014b)
Kyoto	SHG	1928–1969	0.74	L	Y	Kitai et al. (2013)
Locarno	SHG	1958–1980	–	L	N	Schröter and Wiehr (1968)
Madrid	SHG	1912–1917	–	L	N	Vaquero et al. (1912)
Manila	SHG	1965–1984	0.5	L	P	Miller (1965)
McMath-Hulbert	SHG	1948–1979	0.1	L	Y	Mohler and Dodson (1968)
Meudon	SHG	1893–2002	0.15	L	Y	Malherbe (2023)
Mitaka	SHG	1917–1974	0.5	L	Y	Hanaoka (2013)
Mount Wilson	SHG	1915–1985	0.2	L	Y	Lefebvre et al. (2005)
Rome Monte Mario	Filter	1964–1979	0.3	H	P	Chatzistergos et al. (2019a)
Sacramento Peak	SHG	1960–2002	0.5	L	Y	Tlatov et al. (2009)
Schauinsland	SHG	1944–1966	–	L	P	Wöhl (2005)
South Kensington	SHG	1903–1913	–	L	N	Lockyer (1909)
Wendelstein	SHG	1946–1977	–	L	P	Wöhl (2005)
Yerkes	SHG	1899–1930	–	L	P	Hale and Ellerman (1903)
Zürich	Filter	1958–1980	–	L	N	Waldmeier (1968)

^1^ = https://ru.iszf.irk.ru/Телескоп_полного_диска_Солнца_в_линии_KcaII

^2^ = http://craocrimea.ru/en/

**Table 4:** List of digital Ca ii K datasets. Columns are: name of the observatory, type of instrument, approximate period of observations, passband, cadence (H for high and L for low), and bibliography entry

Observatory	Instrument	Period	SW [Å]	Cad	Ref
Baikal	Filter	2003–	0.6	H	^1^
Big Bear	Filter	1996–2006	1.5	L	Naqvi et al. (2010)
Brussels	Filter	2012–	2.7	H	Vanden Broeck et al. (2024)
Calern	Filter	2011–2021	7	H	Meftah et al. (2012)
Calern/Meteospace	Filter	2023–	1.5	H	Malherbe et al. (2019)
Coimbra	SHG	2007–	0.16	L	Garcia et al. (2011)
Haleakalā	Filter	1988–1998	1.2	L	Chatzistergos et al. (2020c)
Helios	Filter	2023–	–	L	^2^
Huairu	Filter	1991–2003	2	L	Deng et al. (1997)
Jindřich$\mathring{\textrm{u}}$v Hradec	Filter	2020–	–	L	^3^
Kanzelhöhe	Filter	2010–	3	H	Pötzi et al. (2021)
Kharkiv	SHG	1994–2021	3	L	Belkina et al. (1996)
Kislovodsk	SHG	2002–	–	L	Tlatov et al. (2015)
Kislovodsk patrol	SHG	2011–	–	H	Tlatov et al. (2015)
Kodaikanal	Filter	1995–2007	2.5	L	Singh and Ravindra (2012)
Kodaikanal Twin	Filter	2008–2013	1.2	L	Singh and Ravindra (2012)
Kodaikanal WARM	Filter	2017–	1	L	Pruthvi (2015)
MLSO/PSPT	Filter	1998–2015	2.7	L	Rast et al. (2008)
Meudon	SHG	2002–	0.15	L	Malherbe and Dalmasse (2019)
Meudon solar tower	Filter	2007–2014	1.5	L	^4^
Mitaka	Filter	2015–	4.5	L	Hanaoka et al. (2020)
Návsí	Filter	2019–	–	L	^3^
Pic du Midi	Filter	2007–	2.5	H	Koechlin et al. (2019)
Picard/SODISM	Filter	2010–2014	7	H	Meftah et al. (2014)
Rome/PSPT	Filter	1996–	2.5	L	Ermolli et al. (2022a)
Rome/PSPT	Filter	2008–	1	L	Ermolli et al. (2022a)
San Fernando CFDT1	Filter	1988–	9	L	Chapman et al. (1997)
San Fernando CFDT2	Filter	1992–	9	L	Chapman et al. (1997)
South Pole	Filter	1981–1994	6–10	H	Jefferies et al. (1988)
Teide ChroTel	Filter	2009–2020	0.3	H	Bethge et al. (2011)
Upice	Filter	1998–	1.6	L	Klimeš et al. (1999)
Valašské Meziříčí	Filter	2013–2018	2.4	L	Lenza et al. (2014)

^1^ = https://ru.iszf.irk.ru/Телескоп_полного_диска_Солнца_в_линии_KcaII

^2^ = https://cesar.esa.int/index.php?Section=Observatories_ESAC_Sun

^3^ = https://space.asu.cas.cz/~sunwatch/

^4^ = https://bass2000.obspm.fr/instru_guide.php#9

#### Ca ii K observations

**Fig. 6:**
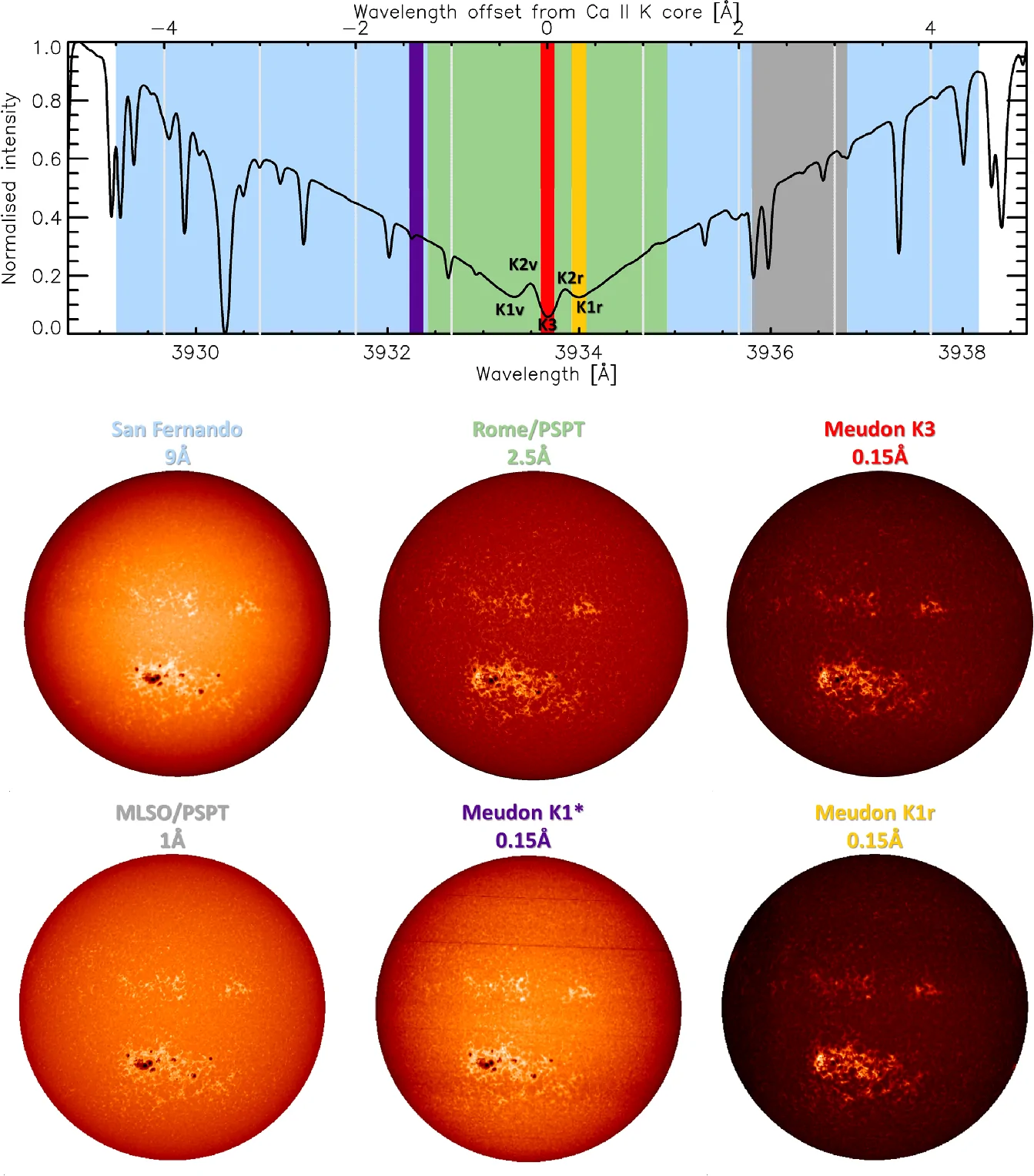
The normalised high-resolution disc-integrated quiet-Sun spectrum over a 10 Å interval centred at the core of the Ca ii K line from the atlas of the Hamburg Observatory (Doerr et al. 2016). Also shown (coloured bands) are three typical nominal bandwidths of Ca ii K archives along with three cases not centred at the core of the line. The passbands centred at the line core shown are 9, 2.5, and 0.15 Å which include the two extreme cases from San Fernando (broadest, light blue) and Meudon (narrowest, red). The vertical light grey lines mark wavelength intervals of 1 Å from the core of the Ca ii K line. The bottom part of the figure presents **six** exemplary observations taken on the same day (11 July 2012) at San Fernando, Rome/PSPT, MLSO/PSPT, and Meudon sites. The archive names above the images are coloured to match their corresponding passbands in the upper panel. The images have been compensated for ephemeris to show the North pole at the top, but no other processing has been applied. The images are shown over their entire respective ranges of values. The values below the names of the observatories are their nominal bandpass. Adapted from Chatzistergos et al. (2024)

Ca ii K line, 3933.67 Å, is the strongest absorption line in the visible spectrum and samples the lower chromosphere. From wing to core it samples, almost continuously, the photosphere to the high chromosphere (Vernazza et al. 1981). It was the first line to be consistently surveyed with a SHG since 1892 (Hale 1893b).

In particular, the first SHG was installed by George Ellery Hale at Kenwood observatory, in Chicago, Ilinois, U.S.A. (Hale 1891) and carried out regular Ca ii K observations between January 1892 and May 1895. This SHG was eventually moved to Yerkes observatory (American Astronomical Society 1931), but due to various upgrades that took place during this time its renewed operation in Yerkes was delayed until the end of 1899 (Hale and Ellerman 1903). In 1904 this SHG was moved to Mount Wilson observatory. In the meantime, the second SHG was installed by Henri-Alexandre Deslandres in Meudon and became functional already in 1892 (Malherbe 2023). Meudon has produced the longest archive of Ca ii K observations spanning more than 130 years, although only sporadic observations were performed between 1893 and 1908. Kodaikanal has currently the second longest archive of Ca ii K observations, covering the period 1904–2007, followed by Coimbra which has exceeded 100 years of observations and continues to this day. Since 1900 numerous other sites used a SHG to perform Ca ii K observations, while filters started being used for Ca ii K observations since 1938. Tables 3 and 4 list some of the available Ca ii K archives. There are more than 50 archives, among which are the prominent long-running ones that we just mentioned, such as Meudon, Kodaikanal, Mount Wilson, and Coimbra, along with numerous relatively short-lived archives, such as the ones in Kandili, Rome, and Haleakalā. These archives provide an almost complete temporal coverage since 1900, but unfortunately there is a gap between 1895 and 1899 when it would appear that only a few sporadic observations at Meudon exist. Over the recent years there are also various amateur observers^3^ that have performed Ca ii K observations (See e.g. review byChatzistergos et al. 2022b). While since 2023 the PRO-AM (Professional-Amateur) collaboration aims at connecting amateur and professional observers by performing Ca ii K observations with a compact SHG that has professional capabilities (Malherbe et al. 2023).

Most extant Ca ii K data are photographic and cover the whole solar disc. There are very few drawings of Ca ii K plage regions available, primarily from the Rome, Kodaikanal, Crimea, and Kenwood observatories, which were, however, likely made by copying existing Ca ii K photographs. Observations up to 2007 were performed in an analogue way, that is mainly stored on glass plates or celluloid films of different sizes. Since the 1980 s, many observatories (all since 2008) have transitioned to using digital detectors. Tables 3 and 4 list most archives that have performed analogue and digital Ca ii K observations, respectively. I note that for archives for which the data have not been recovered and digitised, the dates are either the longest periods I could deduce by published material or estimated based on personal communications.

Over the last decades there has been a huge effort to digitise the available historical Ca ii K archives and allow them to be used for quantitative analyses. Table 3 also lists which of the photographic archives have been digitised by now. In particular, by now 10 archives have been completely digitised (or at least including most of the data), 10 only partially, while 11 archives are completely missing. The data from Anacapri were digitised by Antonucci et al. (1977), however they unfortunately appear to have been lost, which is why they are listed as not digitised in Table 3. By partially digitised I mean cases where only a small fraction of observations have been digitised. That includes the data from Wendelstein, Schauinsland, Kharkiv, Catania, Yerkes, Big Bear, Manila, Kandili, Rome Monte Mario, and Kenwood. Kandili and Rome Monte Mario performed high cadence observations (roughly 100 per day for Rome), but only one and two, respectively, observations per day were digitised from these sites (Chatzistergos et al. 2019a). Other partially digitised archives are because the available digital files were produced from copies of the observations that were sent to other sites. For instance, all Manila Ca ii K observations surviving in digital format are those that were included in the Photographic Journal of the Sun (see Sect. 2.5.8 and Chatzistergos et al. 2020c). The only five available Ca ii K images from Kenwood that are available in digital format were scanned from lantern slides (Chatzistergos et al. 2020c), while the original plates are still to be digitised. Furthermore, some archives underwent multiple digitisations. This was typically motivated by aiming at achieving the best possible quality of the digitised data as well as maintaining consistency in digitising additional data that were missed with earlier digitisations. That affected the Mount Wilson (Foukal 1996; Lefebvre et al. 2005), Kodaikanal (Makarov et al. 2004; Priyal et al. 2014b), Mitaka (Hanaoka 2013), and Meudon (Malherbe 2023) data. Both digitisations of Mount Wilson data included the whole period of observations, but it was done once with an 8-bit camera (Foukal 1996) and then with a 12-bit camera (Lefebvre et al. 2005). The first digitisation of Meudon data covered only those between 1980 and 2002, while the more recent digitisation includes all available observations back to 1893. Mitaka data were digitised three times, with only the first one covering all data with 8-bit depth (Hanaoka 2013). The second and third digitisations were performed with 16-bit depth but with a different setup and covered different periods of data. In particular, the second one covered only the data before 02 March 1960, while the rest of the data along with the first 10 observations of each preceding year were included in third digitisation. The first digitisation of Kodaikanal data included only part of the observations over 1907–1999 with 8bit depth (Makarov et al. 2004), while the second digitisation included more observations covering 1904–2007 with 16bit depth (see Priyal et al. 2014b). It was later realised that many Kodaikanal photographs over 1957–1962 were missed during the second digitisation, thus the entire 1957–1962 period was digitised again with exactly the same way and equipment (Jha et al. 2024). Effects of different digitisations on Kodaikanal data were discussed by Chatzistergos et al. (2019c), supporting the more recent one.

Tables 3 and 4 show that the existing Ca ii K data are quite diverse. The majority of historical archives were performed with a SHG, while most modern data use a filter. Some filtergram archives have high-cadence observations (here referring to observations with at least 1-minute cadence), but all SHG ones are low cadence with only up to a handful of images per day. The pixel scale of the recorded images exhibits huge differences, as well as temporal variations due to changes in instrumentation, with values ranging between 0.9 to 5.5 arcsec per pixel (Chatzistergos et al. 2020c). However, the most important difference among the various archives is their used bandpass. That is because the bandpass controls the height in the solar atmosphere that gets sampled, with narrower bandpasses sampling higher altitudes than broader ones. Figure 6 shows the normalised quiet Sun spectrum around the Ca ii K line marking the nominal bandpasses of some archives. Figure 6 also shows images taken on the same day with different bandpasses illustrating the effect of bandpass on the observations (see also Murabito et al. 2023; Yadav et al. 2026). The existing archives have bandpasses that range between 0.15 Å and 10 Å. As noted by Chatzistergos et al. (2020c), the average nominal bandpass is significantly narrower for most historical archives than modern ones. In particular, Chatzistergos et al. (2020c) noted that the average nominal bandpass remained at about 0.3 Å up to the late 1980 s, while it increased after 1987 to having a mean of about 2–3 Å. It is important to note that the tables list nominal passbands, while the actual ones might differ. Furthermore, changes in instrumentation have also led to changes in the passband of the observations. For instance, over the first years of operation the SHG in Meudon used a single prism, which was upgraded to a 3-prism system in 1908 (D’azambuja 1930) leading to a better dispersed solar spectrum and thus narrower bandwidth observations. Similarly, Mount Wilson upgraded the grating system in the SHG over 21 August 1923, which also affected the passband.

There are various other factors that can affect the quality and stability of observations. For instance, this can be due to changes in the material used to store the observations. An example is given with Kodaikanal which used glass plates to store the photographs up to February 1989, while films were used after that. Relocations of telescopes can also affect the observations, since it changes the seeing conditions. There have been assertions of worsening quality of Kodaikanal Ca ii K data over time (Ermolli et al. 2009b; Chatzistergos et al. 2019b), which was suggested to have arisen due to worsening observing conditions at the Kodaikanal site. It has been suggested that the passband of the Mount Wilson Ca ii K observations during solar cycle 19 was narrower than in other periods (Tlatov et al. 2009; Chatzistergos et al. 2019b), although whether this was a deliberate choice or the result of an instrumental issue remains unclear. Such inconsistencies in the archives must be carefully understood and accounted for in order to mitigate spurious trends and artefacts in subsequent analyses.

All the above refer to Ca ii K observations that were centred at the core of the line. Besides those, there are extensive records of Ca ii K observations that are centred at the K2 maximum, K1 minimum, or the wings of the Ca ii K line. For instance, Meudon (since 1909) and Coimbra (since 1925) kept archives of observations centred at 3932.3 Å (∼ 1.36 Å towards the violet wing of the Ca ii K line). Furthermore, Meudon between 2002 and 2017 performed full-disc observations centred at four different parts of the Ca ii K line (±0.15 Å and ±0.3 Å from the core of the line) along with the ones centred at the core of the line, while since 2017 that increased to 40–100 spectral points with an offset of 0.09 Å (Malherbe and Dalmasse 2019). Sacramento Peak had also observations centred at +0.38 Å from the core of the Ca ii K line, which is roughly the place of the K1 minimum towards the red wing. Finally, MLSO/PSPT also had observations at +2.6 Å from the core of the Ca ii K line. Some examples of off-band Ca ii K observations are shown in Fig. 6 along with their passbands. The above-mentioned archives clearly separated data taken with a different central wavelength, however this was not done with all archives. For example, although observations at Mount Wilson were performed both at the Ca ii K line core and in the wing and the information on the central wavelength and slit width was recorded in logbooks, this information appears to not be available for all years. Furthermore, the digitisation process of Mount Wilson data combined all Ca ii K observations, regardless of whether they were centred on the line core or the wing. I also note that there are a few cases of observations among the digitised archives where the bandpass or the central wavelength changed during the scanning of the solar disc, resulting in parts of the disc sampling different altitude in the solar atmosphere. Combining archives using different bandpasses or central wavelengths as well as with temporal variations in these quantities can introduce significant uncertainties in analyses with such data (Chatzistergos et al. 2020c; Murabito et al. 2023).

**Fig. 7:**
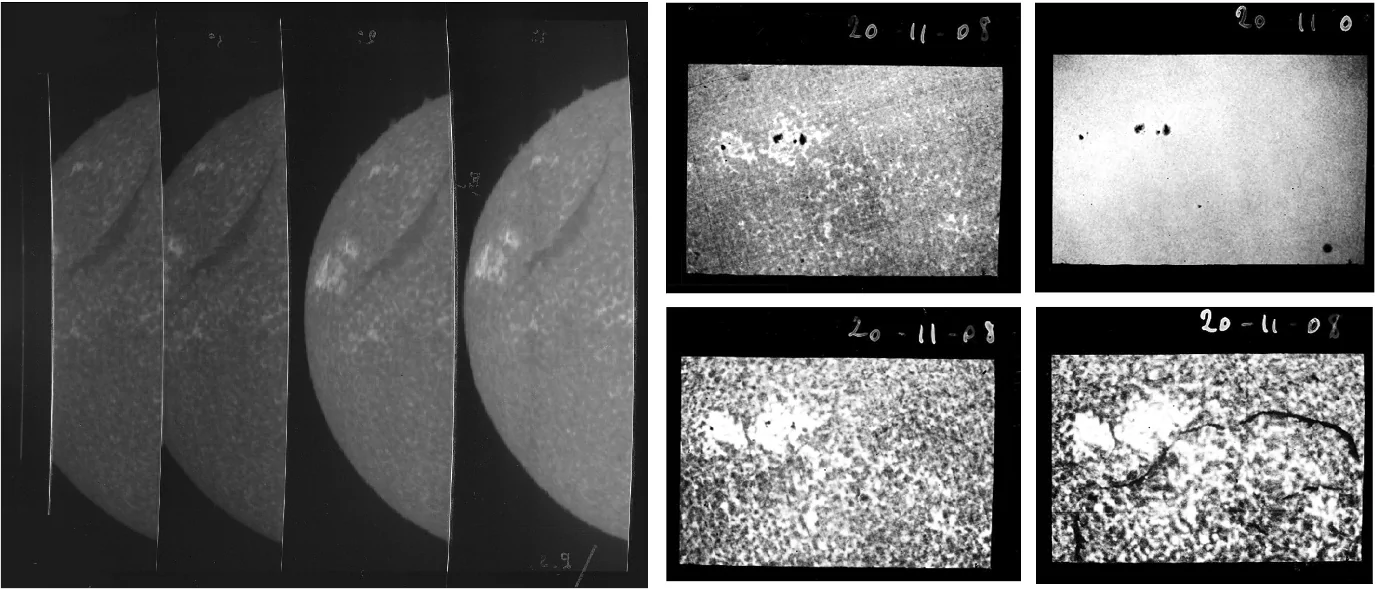
Examples of Ca ii K observations of specific regions of interest. Shown are Meudon observations taken on 21 March 1910 (left) and 20 November 1908 (right): the former includes four exposures obtained with the same settings, while the latter consists of four exposures of the same region centred at different wavelengths across the Ca ii K  line

Besides full-disc Ca ii K  observations, there also exist sporadic historical observations targeting individual regions of interest or partial disc areas. Such data exist, for example, from the Meudon and Kenwood observatories, with two Meudon examples shown in Fig. 7. More recently, high-resolution Ca ii K  observations of regions of interest have been obtained with the Swedish 1-m Solar Telescope (SST; Scharmer 2006) using the CHROMospheric Imaging Spectrometer (CHROMIS; Scharmer 2017). Even higher spatial and spectral resolution over small fields of view has been achieved with observations from the third flight of the balloon-borne Sunrise mission (10–16 July 2024; spectral sampling of 10 mÅ pixel$^{-1}$; Korpi-Lagg et al. 2025; Solanki et al. 2026) as well as with the Daniel K. Inouye Solar Telescope (DKIST; operational since 2020 Rimmele et al. 2020).

**Fig. 8:**
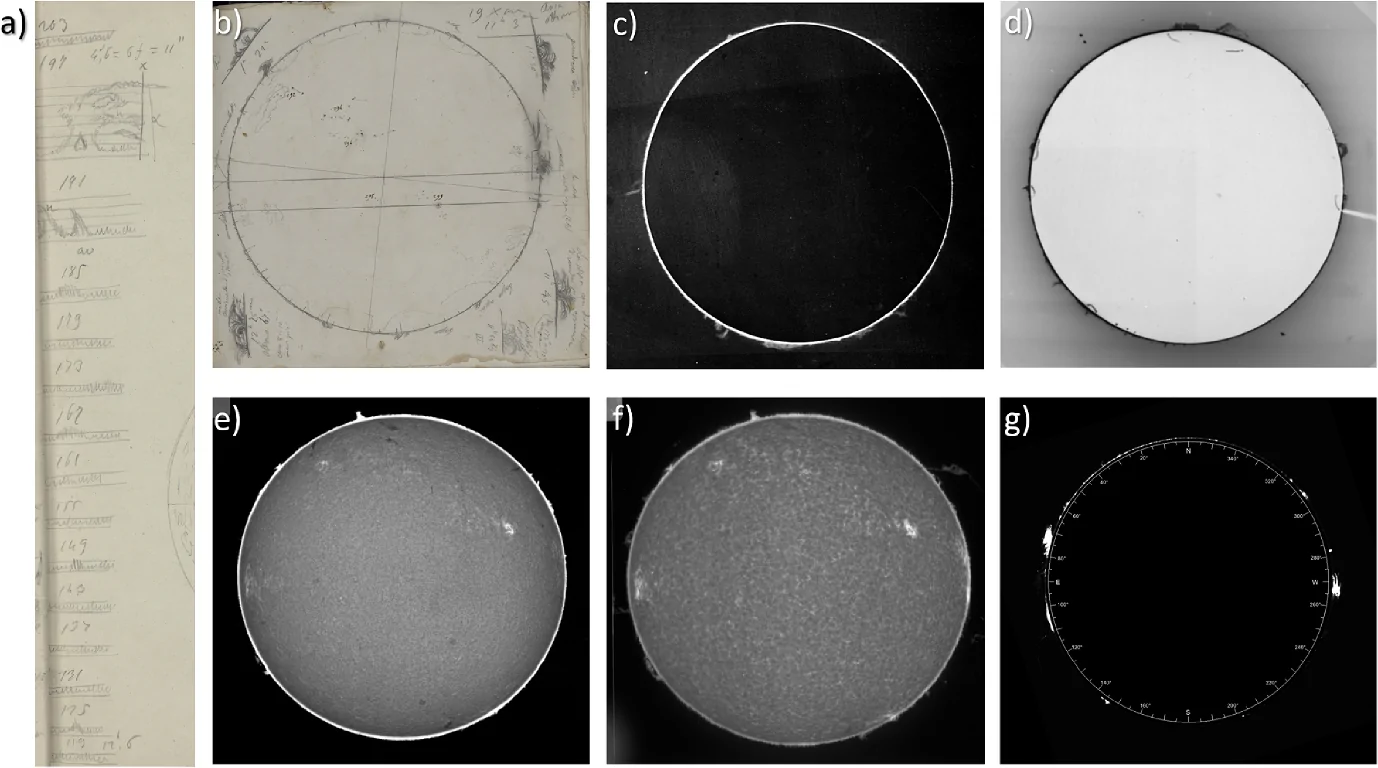
Examples of prominence observations. Drawings by Tacchini from 22 December 1871 (**a**) and Secchi from 19 December 1871 (**b**), Disc-blocked analogue Ca ii K photographs from Meudon (27 June 1894; **c**) and Kodaikanal (2 January 1923; **d**), analogue photographs from Meudon superimposed on full-disc Hα (**e**) and Ca ii K (**f**) observations from 4 February 1998, and disc-blocked digital Hα photograph from Pic du midi from 25 May 2025 (**g**)

There is another type of Ca ii K observations, which was performed by occluding the solar disc so to record prominences and spicules at the solar limb. The earliest such photograph of prominences in disc-blocked Ca ii K observations was performed with a SHG in 21 May 1892 at Kenwood observatory (Hale 1893a). To my knowledge only a few sites performed such observations. In particular, Kenwood, Yerkes, Meudon, Kodaikanal, Cambridge, and South Kensington. For instance, such observations have been performed at Meudon since 1894, however not as extensively as their observations showing the solar disc. Kodaikanal maintained a more extensive dataset of disc-blocked Ca ii K observations over 1906–2002 (Chatterjee et al. 2020). Such disc-blocked Ca ii K data were also maintained at South Kensington and Cambridge over 1904–1942, as well as Yerkes observatory (Rudnick 1934). Two examples of such a disc-blocked Ca ii K observations from Meudon and Kodaikanal are shown in Fig. 8c and d. In some cases, an image with the solar disc blocked is superimposed onto an observation that includes the visible disc. This has been the case for Meudon data since 14 May 1992 (as well as a single observation on 30 May 1991), when a transparent attenuator was introduced, a practice that continues to this day (Malherbe et al. 2022). An example from Meudon is shown in Fig. 8f.

Overall, there is a wealth of Ca ii K archives extending back to 1892. Most of the longest-running archives are currently available in digital format, but there are at least 11 archives whose whereabouts are not known and are still to be digitised. As discussed in the following sections, these observations are invaluable for studying prominences, deriving plage areas, reconstructing past solar magnetism, and reconstructing solar irradiance variations. However, their use requires addressing several challenges, including correcting large-scale image artefacts, accounting for the non-linear response of photographic plates to recover accurate brightness information, and developing robust feature-segmentation techniques. These issues have been addressed using a variety of approaches, which are reviewed in Sect. 2.6. Two major sources of uncertainty nevertheless remain: (1) temporal inconsistencies within individual archives and (2) inconsistencies between different archives. The former mainly arise from instrumental changes that alter the effective passband of the observations, while the latter reflect the lack of a coordinated observing programme, with different archives employing different passbands and therefore sampling different heights in the solar atmosphere.

**Table 5:** List of photographic Hα datasets. Columns are: name of the observatory, type of instrument, approximate period of observations, passband, cadence (H for high and L for low), whether the data have been digitised (Y for yes, N for no or unknown, and P for partly digitised), and bibliography entry

Observatory	Instrument	Period	SW [Å]	Cad	Dig	Ref
Abastumani	SHG	1957–1993	0.5	L	N	Khetsuriani (1967)
Anacapri	Filter	1968–1973^1^	0.7	–	N	Antonucci et al. (1977)
Arcetri	SHG	1931–1968	0.3	L	Y	Ermolli et al. (2009a)
Athens	Filter	1967–1978^1^	0.5	–	N	Macris (1967)
Baikal	Filter	1981–2000	0.5	H	N	Golovko et al. (2002)
Boulder	Filter	1967–1994	0.5	H	Y	Wu et al. (1975)
Cape of Good Hope	Filter	1958–1974	–	–	N	Warner (1979)
Catania	Filter	1967–1998	0.5	P	N	Zuccarello et al. (2011)
Coimbra	SHG	1977–2007	0.26	L	Y	Lourenço et al. (2019)
Crimea	SHG	1955–1979	–	–	N	^2^
Culgoora	Filter	1974–1991	–	H	N	^3^
Ebro	Filter	1957–1980	0.75	H	N	Gaya-Pique et al. (2000)
Ewhurst	SHG	1925–1953	–	L	N	Evershed (1932)
Gran Canaria	Filter	1972–1972	–	L	Y	^4^
Haleakalā	Filter	1968–1970^1^	–	–	N	Kiepenheuer (1969)
Hamburg	SHG	1943–1958	–	L	P	Wöhl (2005)
Haute Province	Filter	1958–1995	0.75	H	P	Malherbe (1958)
Herstmonceux	Filter	1949–1976	–	–	N	Woolley and Harding (1971)
Huancayo	Filter	1960–1992	–	H	N	Ishitsuka et al. (2007)
Kandilli	Filter	1965–1982	0.25, 0.5	H	P	Dizer (1968)
Kanzelhöhe	Filter	1973–2000	0.7	H	Y	Pötzi (2008)
Kharkiv	SHG	1938–1993	0.5	L	P	Belkina et al. (1996)
Kislovodsk	Filter	1959–2010	–	H	N	Tlatov et al. (2015)
Kodaikanal	SHG	1912–2007	0.5	L	Y	Chatterjee et al. (2017a)
Larissa	Filter	1991–1993	0.5	H	N	Chatzistergos et al. (2023a)
Locarno	Filter	1959–1991	–	L	N	Waldmeier (1968)
Lockheed (Burbank)	Filter	1968–1970^1^	0.5	–	N	Smith (1963)
Makapu‘u Point	Filter	1957–1958	0.5	H	N	^5^
Manila	SHG	1965–1984	0.5	L	N	Miller (1965)
Mauna Loa	Filter	1980–1994	–	H	N	^6^
McMath-Hulbert	SHG	1948–1966	0.5	L	Y	Mohler and Dodson (1968)
Meudon	SHG	1909–2002	0.25	L	Y	Malherbe and Dalmasse (2019)
Meudon	Filter	1956–1997	0.75, 0.5	H	P	Malherbe et al. (2019)
Mitaka	Filter	1957–1992	0.75, 0.5	H	Y	Hanaoka et al. (2020)
Mount Wilson	SHG	1915–1985	0.2	L	N	Lefebvre et al. (2005)
Nizamiah		1959–1962	–	H	N	Abhyankar (1961)
Ondřejov	Filter	1960–2001	1	H	N	Švestka (1970)
Sacramento Peak	SHG	1960–2002	0.5	L	Y	Tlatov et al. (2009)
Schauinsland	SHG	1944–1966	–	L	P	Wöhl (2005)
Shamakhy	Filter	1957–	–	–	N	Musayev and Seyidov (2010)
SOON	Filter	1975–1989	0.5	H	N	Neidig et al. (1998)
Ulugh Beg	Filter	1958–2000	0.5	H	N	Scheglov and Slonim (1970)
Tehran	Filter	1968–2005^1^	–	–	N	^7^
Tonantzintla	Filter	1968–1970^1^	–	–	N	^8^
Upice	Filter	1966–1978	–	H	N	Klimeš et al. (1999)
Ussuriysk	Filter	1958–1990	–	H	N	^9^
Wendelstein	SHG	1943–1977	–	L	P	Wöhl (2005)
Yunnan	SHG	1981–1997	0.5	H	N	^10^
Zürich	Filter	1958–1980	–	H	N	Waldmeier (1968)

^1^ = The dates are estimated from Kiepenheuer (1969)–Schröter (1978)

^2^ = http://craocrimea.ru/ru/^3^ = https://www.sws.bom.gov.au/World_Data_Centre/2/7

^4^ = https://www.ncei.noaa.gov/products/space-weather/legacy-data/solar-chromosphere

^5^ = https://web.archive.org/web/20230925033043/https://home.ifa.hawaii.edu/users/steiger/igy_period.htm

^6^ = https://www2.hao.ucar.edu/mlso/instruments/mlso-pics-h-alpha

^7^ = https://web.archive.org/web/20250430131358/https://geophysics.ut.ac.ir/en/w/astronomy-and-solar-physics-research-division?p_l_back_url=%2Fen%2Fsearch%3Fq%3Dsun

^8^ = https://astro.inaoep.mx/en/observatories/oanton/solar-telescope/sequence-of-images

^9^ = https://astrogalaxy.ru/514.html

^10^ = https://english.ynao.cas.cn/au/histroy/

**Table 6:** List of digital Hα datasets. Columns are: name of the observatory, type of instrument, approximate period of observations, spectral width of the spectrograph, and the bibliography entry

Observatory	Instrument	Period	SW [Å]	Cad	Ref
Athens	Filter	2015–	0.5	L	Kontogiannis and Tsiropoula (2016)
Baikal	Filter	2000–	0.5	H	Golovko et al. (2002)
Big Bear	Filter	1988–	0.5	H	Denker et al. (1999)
Big Bear GONG	Filter	2010–	0.4	L	Harvey et al. (2011)
Brussels	Filter	2002–	0.5	H	Vanden Broeck et al. (2024)
Calern/Meteospace	Filter	2023–	0.34	H	Malherbe et al. (2019)
Catania	Filter	1998–	0.5	H	Romano et al. (2022)
Cerro Tololo GONG	Filter	2010–	0.4	L	Harvey et al. (2011)
CHASE	Filter	2021–	0.14	H	Li et al. (2019)
Coimbra	SHG	2008–	0.26	L	Lourenço et al. (2019)
Culgoora	Filter	2003–2014	–	H	^1^
El Leoncito	Filter	1998–	0.3	H	Francile et al. (2008)
Helios	Filter	2012–	0.5	L	^2^
Hida FMT	Filter	1992–2010	–	H	Ueno et al. (2007)
Hida SMART	Filter	2005–	2	L	Ueno et al. (2004)
Hiraiso	Filter	1994–2006	0.25	H	Akioka (1996)
Huairu	Filter	1991–2004	0.5	H	Deng et al. (1997)
Huairu SMAT	Filter	2005–	0.25	H	Zhang et al. (2007)
Hurbanovo	Filter	2020–	0.7	L	^3^
Ica FMT	Filter	2010–	–	H	Ueno et al. (2014)
ISOON	Filter	2003–2012	–	H	Neidig et al. (1998)
Kanzelhöhe	Filter	1998–	0.7	H	Pötzi et al. (2021)
Kharkiv	SHG	1994–2005	0.5	L	Belkina et al. (1996)
Kislovodsk	SHG	2011–	–	L	Tlatov et al. (2015)
Kislovodsk patrol	SHG	2011–	–	H	Tlatov et al. (2015)
Kodaikanal	Filter	2014–	0.4	H	Ravindra et al. (2016)
Larissa	Filter	2008–2015	0.5	L	Chatzistergos et al. (2023a)
Learmonth GONG	Filter	2010–	0.4	L	Harvey et al. (2011)
Mauna Loa	Filter	1994–2011	–	H	^4^
Mauna Loa GONG	Filter	2010–	0.4	L	Harvey et al. (2011)
Meudon	SHG	2002–	0.15	L	Malherbe and Dalmasse (2019)
Meudon	Filter	1999–2004	0.5	H	Malherbe et al. (2019)
Meudon solar tower	Filter	2007–2014	0.5	L	^5^
Mitaka AFPT	Filter	1991–2019	0.5	H	Hanaoka et al. (2020)
Mitaka	Filter	2011–	0.25	H	Hanaoka et al. (2020)
NSO/FDP	Filter	2011– 2017	–	H	^6^
Ondřejov	Filter	2002–	0.7	L	^7^
Pardubice	Filter	2018	–	L	^8^
Pic du Midi	Filter	2007–	0.5	H	Koechlin et al. (2019)
Riyadh FMT	Filter	2015–	–	H	Ueno (2015)
SOON	Filter	1989–1997	0.5	H	Neidig et al. (1998)
Teide	Filter	1996–2004	0.5, 1	L	^9^
Teide GONG	Filter	2010–	0.4	L	Harvey et al. (2011)
Teide ChroTel	Filter	2009–2020	0.3	H	Bethge et al. (2011)
Udaipur GONG	Filter	2010–	0.4	L	Harvey et al. (2011)
Upice	Filter	1998–	0.8	L	Klimeš et al. (1999)
Valašské Meziříčí	Filter	2012–2018	0.7	L	Lenza et al. (2014)
Yunnan	Filter	1997–2009	0.5	H	^10^
Yunnan ONSET	Filter	2011–	0.25	H	Fang et al. (2012)

^1^ = https://www.sws.bom.gov.au/World_Data_Centre/2/7

^2^ = https://cesar.esa.int/index.php?Section=Observatories_ESAC_Sun

^3^ = https://www.kozmos-online.sk/obs/aktivita/archochromdisk.htm

^4^ = https://www2.hao.ucar.edu/mlso/instruments/mlso-pics-h-alpha

^5^ = https://bass2000.obspm.fr/instru_guide.php#9

^6^ = https://nso.edu/telescopes/nisp/fdp/

^7^ = https://space.asu.cas.cz/~sunwatch/

^8^ = https://space.asu.cas.cz/~sunwatch/new/www/public/files/archive_foreign/Pardubice/2018/

^9^ = https://web.archive.org/web/20150113160853/http://www3.kis.uni-freiburg.de/halpha_e.html

^10^ = https://www.bbso.njit.edu/Research/Halpha/logs/ynao_log/

#### Hα observations

Hα line, 6562.79 Å, is one of the broadest and deepest lines in the visible solar spectrum. From the wing to the core, it distinctly samples the deep photosphere and chromosphere (Vernazza et al. 1981). It was one of the earliest lines used for sampling the solar chromosphere. In fact, Hα observations predate Ca ii K ones by about two decades. The early Hα observations were done visually with a SHS (see Sect. 2.1.1), which however did not allow at that time to observe any feature within the solar disc (Hale 1929), but allowed only to record prominences outside of the limb. On-disc Hα observations became feasible with the development of the SHG, and later, through the use of monochromatic filters, but also with the improvements in the SHS. Furthermore, early observations in the Hα line faced another significant challenge: traditional photographic plates at that time were not sensitive to wavelengths longer than approximately 5900 Å. This limitation was overcome in 1907 with the invention of red-sensitized emulsions (Wallace 1907), enabling the recording of solar on-disc and off-limb Hα observations on photographic plates beginning in 1908. Thus, all pre-1908 Hα observations are either visual or pictorial.

The early SHS Hα observations represent a valuable historical dataset for studying solar prominences (Lockyer 1903; Bocchino 1933a) since they are not only visual and pictorial representations, but sometimes also include detailed tabulations of prominence characteristics, such as their positions, extents, and areas. Notably, prominent solar physicists such as Janssen, Lockyer, Secchi, Tacchini, and Ricco began performing such observations as early as 1871 (e.g. Carrasco et al. 2021b; Ermolli et al. 2023), while Wolfer also carried out prominence observations between 1888 and 1920 (Illarionov and Arlt 2022). Figure 8a and b shows examples of prominence drawings by Tacchini and Secchi, respectively. Such off-limb Hα observations continued to be performed even after the introduction of the SHG. For instance, there are SHS observations of prominences from Ulugh Beg (previously called Tashkent) observatory since 1932 (Ivanov 1934; Karachik et al. 2025). Drawings of prominences were routinely made at several observatories, including Locarno, where an extensive collection by S. Cortesi is archived and publicly available online.^4^ Additional prominence drawings were recorded in Odessa at least during 1892–1893 (Hale 1896), while Eric H. Strach recorded prominence characteristics in Hα over 1974–2008 (Carrasco et al. 2019).

Photographic observations of solar prominences in the Hα line by blocking the solar disc have been carried out at various observatories since 1908 too. For instance, early photographic observations were conducted at Mount Wilson Observatory between April 1908 and 1909 (Abetti and Smith 1911), in Ulugh Beg over 1958–2000, and Mitaka over 1978–1998. More recent photographic disc-blocked prominence Hα archives include observations from Valašské Meziříčí (1983–2010), Pic du Midi (high-cadence Hα data since 2011), the SMART telescope at Hida Observatory (Ueno et al. 2004), as well as Meudon (since 30 May 1991). Meudon in particular, superimposed disc-blocked Hα observations onto solar disc Hα observations (Malherbe et al. 2022). Examples of a disc-blocked Hα observations from Pic du Midi and Meudon are shown in Fig. 8g and e, respectively. Together, these historical datasets provide a critical foundation for long-term investigations of solar prominences and their temporal evolution (see Sect. 3.6; Tandberg-Hanssen 1974, 1995; Parenti 2014).

On-disc Hα observations have also been performed since 1908 with SHG, as well as with filters and the upgraded SHS since at least the 1930 s. The upgraded SHS and monochromatic filters proved especially valuable for Hα imaging due to the need for high-cadence observations to capture rapidly evolving solar phenomena such as flares (See Sect. 3.7). This led to the establishment of many flare patrol telescopes, for example the Flare Monitoring Telescopes (FMT) at Hida, Ica, and Riyadh (Ueno 2015) or the Monochromatic Heliograph (1957–1992), Automatic Flare Patrol Telescope (AFPT; 1991–2019), and Solar Flare Telescope (since 2011) at Mitaka (Hanaoka et al. 2020), as well as Big Bear, Catania, Kanzelhöhe, and Boulder. In fact, monitoring of flares motivated the International Geophysical Year (IGY) to coordinate high-cadence solar observations in Hα , which were conducted at numerous sites around the globe.

**Fig. 9:**
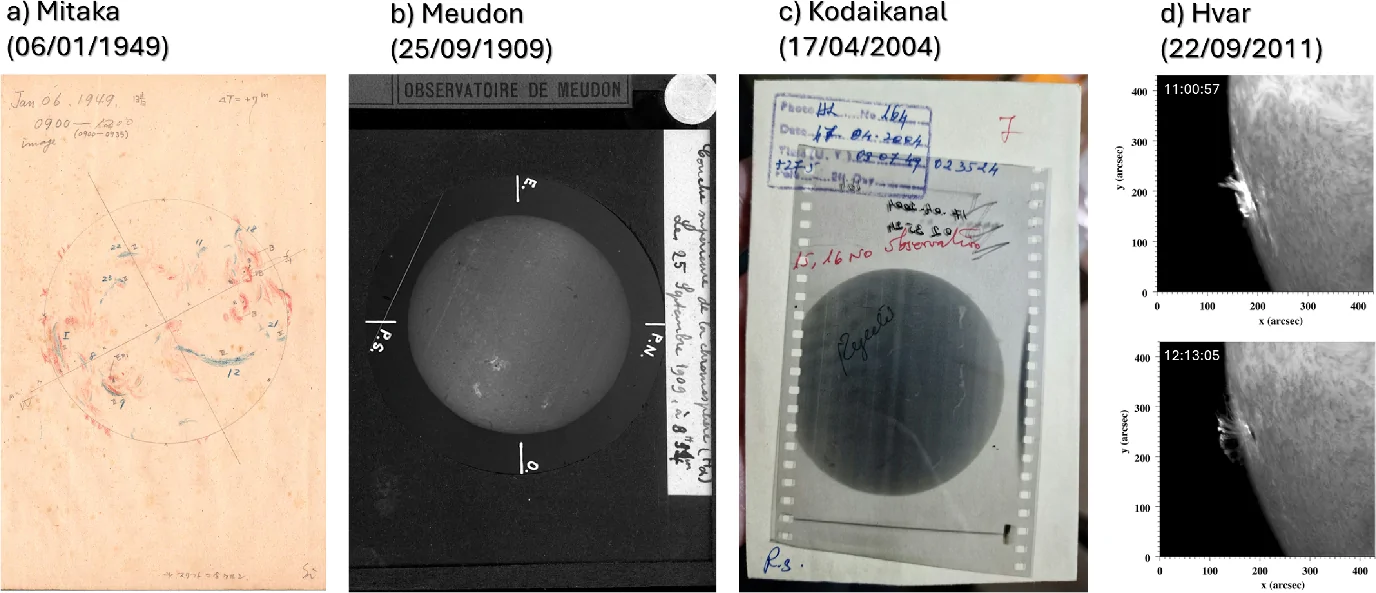
Examples of Hα observations. Shown are an SHS drawing from Mitaka (**a**), one of the earliest SHG photographs from Meudon (**b**), an SHG photograph from Kodaikanal (**c**; note that this is a photograph of the original film rather than an image from the properly digitised Kodaikanal archive), and two photographs of a single active region from the Hvar Observatory (**d**)

The extent to which visual on-disc Hα observations were carried out using SHSs or narrowband filters across different sites remains unclear. Nevertheless, several well-documented examples exist. Hale (1931) gave a list of 25 observatories that were equipped to perform observations with a SHS. At the Ondřejov Observatory, the Sun was visually observed in Hα using a SHS between 1939 and 1970 (Kotrc 2009). Mitaka had visual Hα observations with a SHS over 1949–1964, resulting to drawings (Yamaguchi 1991), which have since been digitized and made publicly accessible.^5^ Eric H. Strach employed Hα filters for visual solar observations and maintained a systematic archive of drawings spanning 1978–2008 (Carrasco et al. 2019). In addition, visual Hα observations were reported from Kanzelhöhe Observatory between 1946 and 1973 (Pötzi et al. 2016) as well as from Northwestern Observatory in Spokane, Washington, during the period 1958–1970. An example SHS Hα drawing is shown in Fig. 9a.

However, most of the available on-disc Hα data are photographic, some of which are listed in Tables 5 and 6. The earliest known photographic Hα observations using a SHG were likely carried at Yerkes in 1908, followed by Meudon in 1909, while Kodaikanal initiated similar observations just three years later. One of the earliest such Hα SHG photographs is shown in Fig. 9b. Since then there have been more than 70 archives with photographic Hα observations. All sites use a digital detector since 2010, while early digital data started being collected already in the late 1980 s. Over the recent years there are also various amateur observers that perform Hα observations (Chatzistergos et al. 2022b).^6^ The longest running Hα archives are from Meudon, Kodaikanal, Mitaka, and Kanzelhöhe, all of which continue observations to this day, although after various instrumental upgrades. It is likely that many archives are missing from Tables 5 and 6. For instance, Fokker et al. (1971) note that Sonnenborgh Observatory in Utrecht conducted Hα observations using a Lyot filter; however, it remains unclear whether these observations were photographic or whether any records have survived, and thus they are not mentioned in the tables.

Great efforts have been invested over the recent years to digitise the available Hα data too. Attesting to the importance of locating and digitising all available historical observations is the case of the Mitaka Monochromatic Heliograph Hα data, the films of which deteriorated and had to be discarded, fortunately only after being digitised. To the best of my knowledge only 11 historical Hα archives have been completely digitised, 6 only partially, and at least 32 Hα archives have not been digitised yet. Like for the Ca ii K data, Meudon Hα data were digitised two times, the first one covered only the data between 1980 and 2002, while the more recent digitisation includes all available observations. Inconsistencies in the digitised archives due to changes in the instrumentation are important to be understood in order to perform meaningful analyses with these data. For instance, Kodaikanal data switched from photographic plates to film in 1978, while observations after 1998 often have missing solar disc sections (Chatzistergos et al. 2023a, see also Fig. 9c, affecting the quality of the data over these periods.

**Fig. 10:**
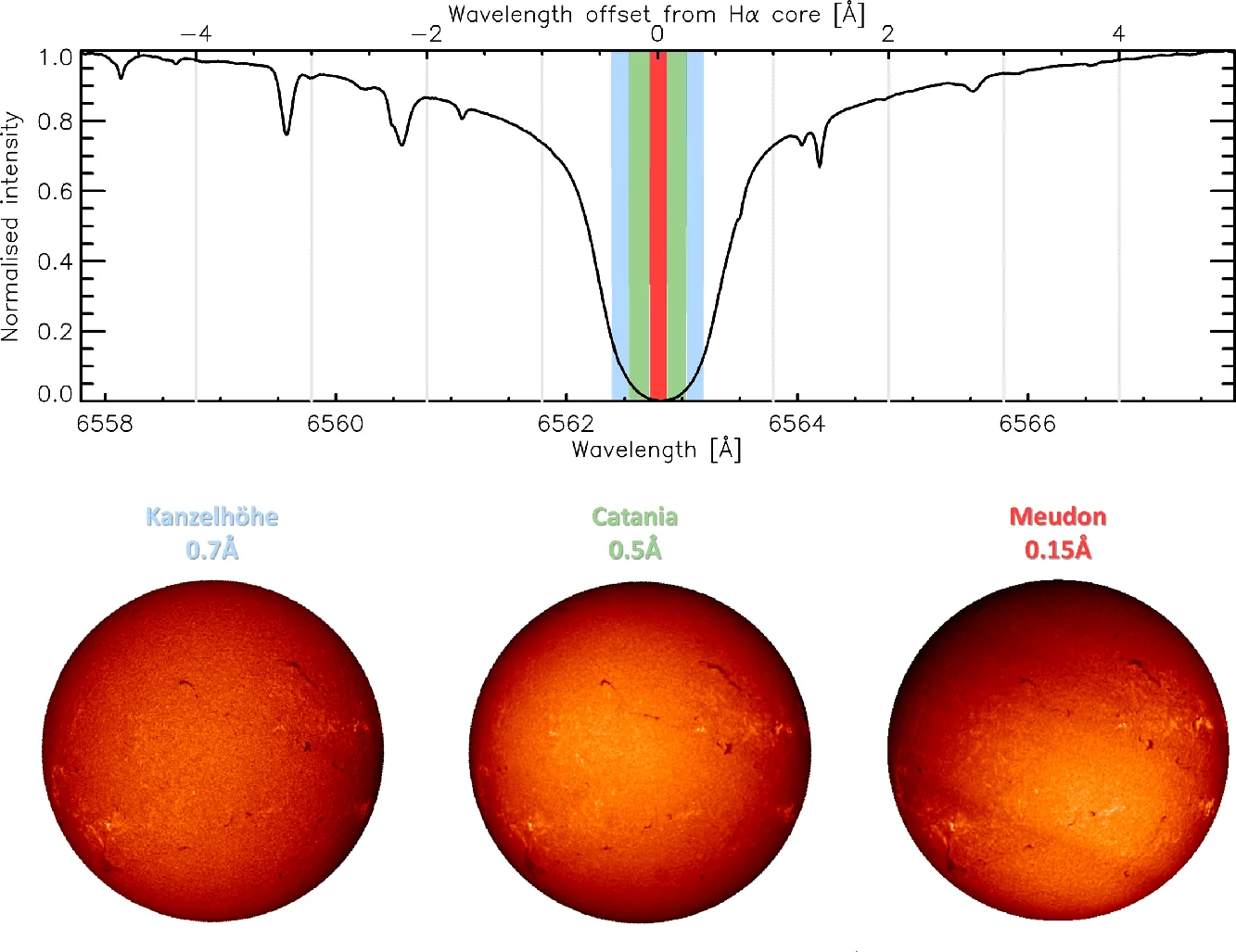
The normalised high-resolution disc-integrated quiet-Sun spectrum over a 10Å interval centred at the core of the Hα line from the atlas of the Hamburg Observatory (Doerr et al. 2016). Also shown (coloured bands) are three cases of nominal bandwidth of Hα archives. Shown are 0.7, 0.5, and 0.15 Å which roughly correspond to the two extreme cases from Kanzelhöhe (broadest, light blue) and Meudon (narrowest, red) as well as a typical bandwidth from Catania (green). The vertical light grey lines mark wavelength intervals of 1 Å from the core of the Hα line. The bottom part of the figure presents three exemplary observations taken on the same day (6 September 2013) at Kanzelhöhe, Catania, and Meudon sites. The images have been compensated for ephemeris to show the North pole at the top, but no other processing has been applied. The images are shown over their entire respective ranges of values

Similarly to the Ca ii K data, differences in bandpass, central wavelength, as well as temporal variations of these parameters and instrumental and observational conditions affect the consistency of the archives. Figure 10 shows the normalised quiet Sun spectrum around the Hα line along with some common nominal bandpasses of existing archives. Overall, the Hα archives are more consistent with each other than the Ca ii K archives in terms of their nominal bandpasses. In particular, the majority of Hα datasets, whether obtained with SHG or narrowband filters, employ bandpasses close to 0.5 Å. In terms of temporal coverage, Chatzistergos et al. (2023a) reported an effectively complete coverage since 1968 (99.7%). However, there are relatively few digitised Hα archives before that, which significantly reduces the temporal coverage of Hα data before 1950. This highlights the importance of locating and digitising the early Hα archives. Most of the archives mentioned here refer to full-disc observations, but there are also Hα observations of individual regions of interest. This is the case for the archives from Hvar observatory (Dumbovic et al. 2024, covering the period 2010–2019; Fig. 9d, IBIS (Cavallini 2006; Ermolli et al. 2022b, covering 2013–2019), and THEMIS (Schmieder et al. 2025, since 1998).

Overall, there is a wealth of Hα archives extending back to 1908. Most of the longest-running archives have been digitised, but there are at least 32 archives whose data have not been digitised or even located. These observations are essential for deriving information about filaments and prominences. Since the brightness of these features is typically of secondary importance for most studies, which mainly require accurate positions and temporal evolution, correcting for the non-linear response of the photographic plates is generally less critical than for Ca ii K observations. The existing Hα archives are also reasonably consistent in terms of their employed passbands, making results from different archives largely comparable, although caution is still warranted when combining observations with significant passband differences. The main sources of uncertainty in studies based on Hα observations are temporal inconsistencies within individual archives and large-scale image artefacts, which are generally more pronounced than in Ca ii K observations.

**Fig. 11:**
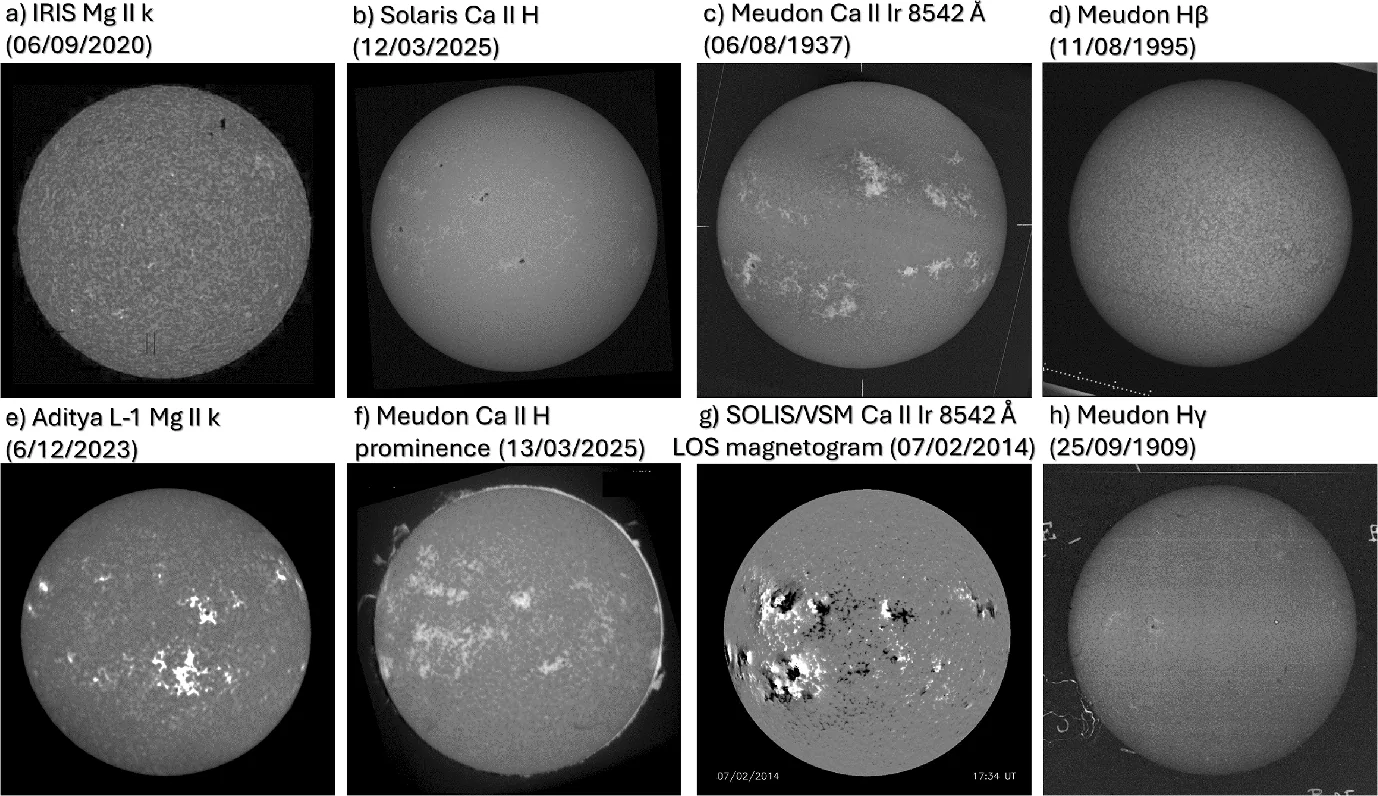
Representative observations in the Mg ii k (panels a and e), Ca ii H (panels b and f), Ca ii infrared 8542 Å (panels c and g), Hβ (panel d), and Hγ (panel h) lines. Panel b: Image credit Solaris Observatory, Space Valley Israel, Israel Space Agency (ISA), Ministry of Innovation, Science and Technology - Israel. Panel e:Aditya-L1 image ©Indian Space Research Organisation (ISRO). Panel g: Image credit NSO/AURA/NSF

#### Other chromospheric observations

The Ca ii H, Ca ii Infrared, Hβ, Hγ, and Mg ii h and k lines have also been used for spatially resolved observations over the last decades, but they have been monitored significantly less than Ca ii K and Hα . To my knowledge there are only a few sporadic historical SHG observations in the Ca ii infrared triplet. In particular, I could locate 11 observations from Meudon over 1928–1947 centred in the Ca ii infrared line at 8542 Å and merely two at 8498 Å (D’azambuja 1930). Ca ii infrared observations with a small field of view were performed also during the 3rd flight of the balloon-borne Sunrise mission (Korpi-Lagg et al. 2025; Solanki et al. 2026) over 2024 as well as with IBIS (Cavallini 2006; Ermolli et al. 2022b) over 2012–2019. I am not aware of any historical observation in the 8662 Å Ca ii infrared line. Over 2003–2013 the SOLIS VSM (Keller et al. 2003) produced chromospheric vector magnetograms in the 8542 Å line. Examples of Ca ii Infrared observations at 8542 Å  are shown in Fig. 11c and g for Meudon and SOLIS VSM, respectively.

The only mention of historical Ca ii H , 3969 Å, observations that I could locate is about Mount Wilson data between June 1906 and April 1908 with the aim of observing prominences (Abetti and Smith 1911). Recently there have been, however, modern archives using filter to observe the Sun in the Ca ii H line. For example, Meudon acquired such Ca ii H filtergrams between 2009 and 2014, while their SHG was updated in 2017 (Malherbe and Dalmasse 2019) to perform routine observations in Ca ii H by including 40–100 spectral points with an offset of 0.09 Å (Malherbe and Dalmasse 2019). Notably, Meudon performs also prominence observations in the Ca ii H line by superimposing them onto solar disc Ca ii H observations (Fig. 11f). Furthermore, the Solaris observatory^7^ at Isfiya started performing Ca ii H observations in 2025, while the space-based Aditya-L1 Solar Ultraviolet Imaging Telescope (SUIT; Tripathi et al. 2025) since 2023. Ca ii H observations with a small field of view were performed during all three flights of the balloon-borne Sunrise mission (Solanki et al. 2010, 2017; Korpi-Lagg et al. 2025) over 2009, 2013, and 2024, respectively. Examples of Ca ii H observations are shown in Fig. 11b and f for Isfiya and Meudon, respectively.

To my knowledge there have been no systematic observations in either Hβ or Hγ lines. There are only some very sporadic SHG observations in the 4341 Å  Hγ line from Meudon during 1909, as well as Hβ 4861 Å from Meudon over 1995 and 2017–2019 as well as from Yerkes observatory since 1903 (Hale and Ellerman 1903; Ellerman 1919). Examples of Meudon Hβ and Hγ observations are shown in Fig. 11d and h, respectively.

There are no historical observations in the Mg ii h and k lines (2802.7 Å and 2795.5 Å, respectively), as these lines are not observable from the ground. Such observations became possible with space-based missions, however there is still significant scarcity with such data. For instance, there are spatially resolved spectra around the Mg ii h and k lines over 1980–1989 with the ultraviolet spectrometer and polarimeter on the Solar Maximum Mission (Woodgate et al. 1980). Mg ii h and k observations with a small field of view were performed during the first two flights of the balloon-borne Sunrise mission (Solanki et al. 2010, 2017) over 2009 and 2013, respectively. Since 2013 the Interface Region Imaging Spectrograph (IRIS; Kerr et al. 2015) performs observations around the Mg ii h and k lines with a spectral sampling of 25 mÅ pixel$^{-1}$ and a small field of view, while almost on a monthly basis they produce a full-disc mosaic. More recently, Aditya-L1 SUIT (Tripathi et al. 2025) started acquiring Mg ii h and k full-disc observations since 2023. Examples of Mg ii k observations are shown in Fig. 11a and e for IRIS and Aditya-L1, respectively.

**Fig. 12:**
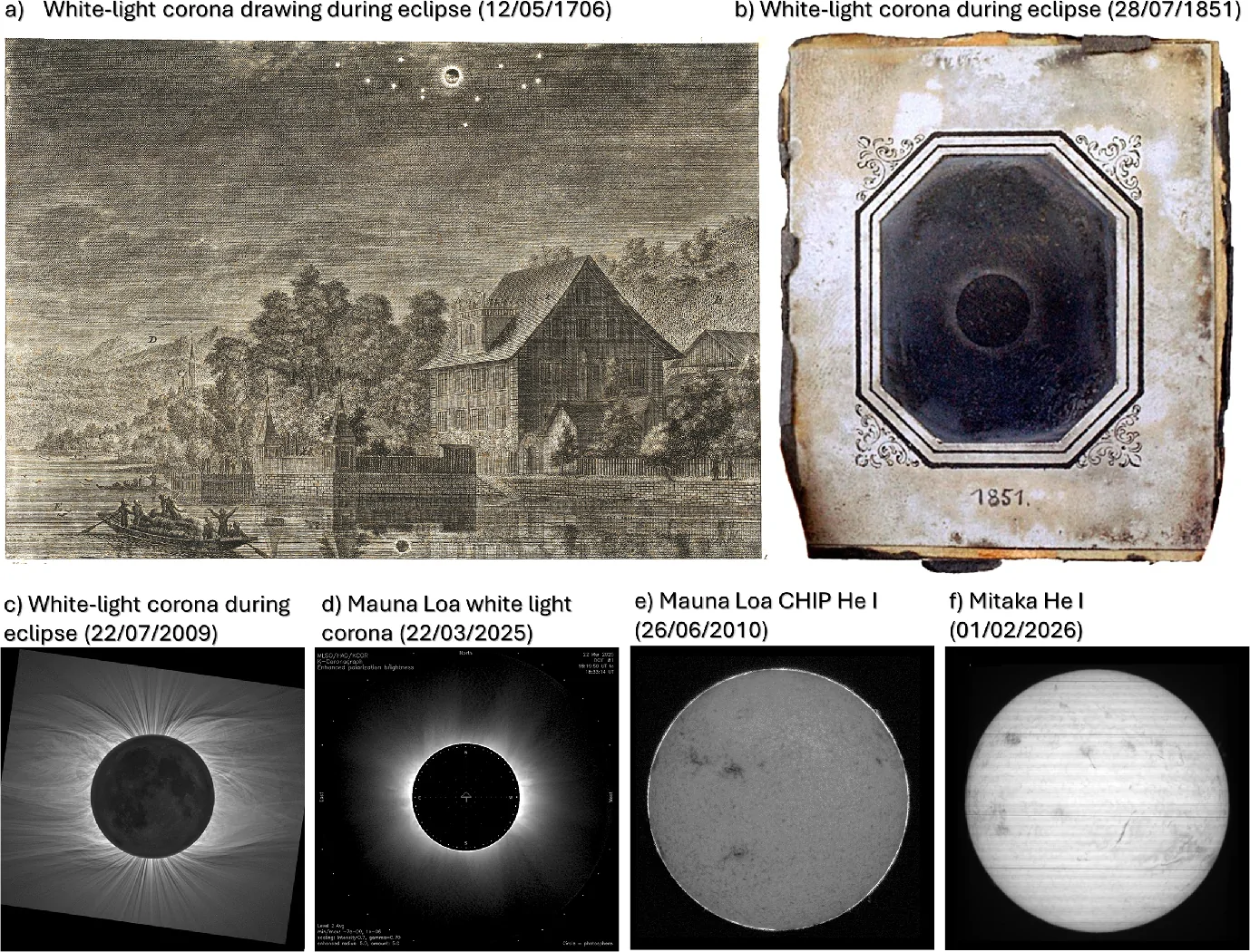
Examples of corona observations. Panels (**a**–**d**) show white-light coronal observations. The first three panels were obtained during solar eclipses, whereas panel (**d**) was acquired using the Mauna Loa coronagraph. In particular, panel (**a**) shows a drawing made during the 12 May 1706 eclipse by Füssli (1706), panel (**b**) presents the first photograph of a solar eclipse from 28 July 1851 by Berkowski (©Friedrich-Schiller-Universität: Historische Sammlungen zur Naturwissenschaft), while panel (**c**) is a digital photograph of the 22 July 2009 Eclipse by Miloslav Druckmüller. Panels (**e** and **f**) show He i 10830 Å line observations from Mauna Loa and Mitaka, respectively

### Coronal observations

Observations of the solar corona, which had been initially limited to rare total eclipses, became routine in the 1930 s following Bernard Lyot’s invention of the coronagraph (Lyot 1930, 1939). These developments allowed for regular monitoring of coronal structures, which became even more detailed with the advent of space-based imaging. There exist various types of observations of the solar corona (see, e.g. Poletto 2015), particularly those enabled by space-based missions in spectral lines inaccessible from the ground. Here, however, we focus only on historical spatially resolved observations in white light (Sect. 2.4.1) and in the He i 10830 Å line (Sect. 2.4.2), which were possible from ground-based sites.

#### White-light observations

Besides the solar photosphere, white light observations can also reveal parts of the solar Corona, what is called the K corona. However, the brightness of the photosphere makes it difficult to observe it, which is why white-light Corona was initially observed only during eclipses (e.g. Lockyer 1931; Bugoslavskaia 1950; Rušin et al. 2020). Various records of solar eclipses exist, which carry information about white-light corona (see e.g. Owens et al. 2017; Hayakawa et al. 2021). Figure 12a shows a drawing of the 12 May 1706 solar eclipse by Füssli (1706) revealing the white-light solar corona too. The first photograph of white-light corona was likely taken by Berkowski during the 28 July 1851 eclipse^8^ in Königsberg (modern-day Kaliningrad) with a daguerreotype (shown in Fig. 12b). Numerous such photographs have been taken over the years. For instance, Fig. 12 panel c shows an image of white-light corona during an eclipse taken by Miloslav Druckmüller.^9^ It is worth noting the citizen-science initiative Citizen Continental-America Telescopic Eclipse (CATE; Penn et al. 2019), which obtained a continuous time series of white-light coronal observations during the total solar eclipse of 21 August 2017. Its successor, CATE 2024 (DeForest et al. 2024; Kovac et al. 2026), deployed 43 identical telescope-camera systems along the eclipse path on 8 April 2024, with 36 trained citizen-science teams capturing a 60-minute polarized white-light movie of the inner and middle solar corona across North America. Eddy and Goff (1971); Eddy and Hansen (1973) collected more than 350 photographic plates of white light corona during eclipses between 1869 and 1971, while this database was later expanded by Judge et al. (2010)^10^ as well as in Miloslav Druckmüller’s website.^11^ Earlier visual records of eclipse observations with information about white light corona were collected by Owens et al. (2017) and Hayakawa et al. (2021). In particular, Owens et al. (2017) found information for 60 eclipses between 1666 and 2013 (10 of which had more than one report), while Hayakawa et al. (2021) identified 52 additional reports from 12 eclipses over 1652–2019. Furthermore, observations of the corona in numerous spectral lines have been performed from the ground during solar eclipses (see, e.g., Landi et al. 2016; Del Zanna and DeLuca 2018). The earliest such observation is likely the detection of the Fe XIV 5303 Å “green coronal line” during the eclipse of 7 August 1869 (Rybanský et al. 2005).

The development of the coronagraph by Bernard Lyot in the 1930’s (Lyot 1930, 1939) allowed observing the K-corona at any instant (e.g. Eddy and Hansen 1973). Observations over various spectral lines have been performed with a coronagraph over the years from ground, while space-based measurements have been available since the Skylab mission in 1973 (Vaiana et al. 1973), enabling the sampling of lines that are not accessible from the ground. Since 1930 observations of the Fe XIV 5303 Å “green coronal line”, as well as the Fe X 6374 Å “red line” and the Ca XV 5694 Å “yellow line” among many other lines have been conducted with the use of a coronagraph (Lyot 1930, 1939; Waldmeier 1941). The colour designation reflects the approximate formation temperature of these ions, with the green, yellow, and red lines corresponding to progressively higher temperatures. These records constitute one of the longest continuous datasets available for studies of the solar corona (Rybanský et al. 2005). Observations of the Ca XV 5694 Å yellow line, however, are limited to periods of strong solar activity and therefore do not extend continuously over time. As a consequence, systematic long-term observations exist primarily for the green coronal line, whereas those for the yellow and red lines are more limited.

Systematic observations in the Fe XIV 5303 Å line have been carried out for many decades at several major observatories using a range of instruments. Among the earliest long-term observing programs were those at Pic du Midi (since 1930; Lyot 1930, 1939), followed by sustained efforts at Arosa (1939–1980; Waldmeier 1941. Subsequent extensive series include the observations at Norikura Observatory from 1951 to 2009 Sakurai 2012, as well as the continuous datasets obtained at Kislovodsk (since 1950; Guseva 2019) and Lomnický štít (since 1964). These key ground-based records are complemented by measurements from Sacramento Peak (since 1951; Evans 1967; Altrock 2014) and Wendelstein observatories, and later by space-based observations from the Solar Maximum Mission coronagraph during 1980–1989 MacQueen et al. 1980. Furthermore, the Ca XV 5694 Å “yellow line” was observed at Climax from at least 1946 to 1961 (Dolder et al. 1954; Trotter and Billings 1962), providing complementary diagnostics of hotter coronal plasma. Yellow and green coronal measurements were also reported from Abastumani for at least the period 1971–1972 (Tetruashvili 1974). The High Altitude Observatory began daily measurements of the inner white-light corona in the mid-1950s at Climax, Colorado, following the introduction of the K-coronameter (Wlérick and Axtell 1957). The program expanded in 1963 when the instrument was relocated to Hawaii, first to the University of Hawaii’s Haleakalā Observatory and, since 1965, to Mauna Loa Observatory^12^ (Garcia et al. 1971). Figure 12d shows an image of white-light corona outside of an eclipse with the Mauna Loa observatory coronagraph.

#### He i 10830 Å observations

Another important diagnostic for coronal studies is through observations in the He i 10830 Å infrared line (Babcock and Babcock 1934). The He i 10830 Å line appears in absorption on the solar disc but can switch to emission during events like solar flares (Zirin and Howard 1966). Its absorption strengthens above active regions and filaments, while weakening in coronal holes due to a complex line-formation process influenced by the upper chromosphere, transition region, and low corona (Avrett et al. 1994; Harvey and Recely 2002; Lagg 2007). The He i 10830 Å line is thus particularly important for studying dynamic solar phenomena such as flares, CMEs, and the evolution of filaments and prominences (Rueedi et al. 1995).

The first observation in the He i 10830 Å line was conducted at the Meudon Observatory on 17 September 1938 (Malherbe et al. 2022), although no regular monitoring was established at that time. Systematic observations began in 1974 at the National Solar Observatory (NSO) at Kitt Peak, where daily full-disc measurements have been used to produce intensity maps of the line’s equivalent width (Livingston et al. 1976). These datasets, archived by National Oceanic and Atmospheric Administration (NOAA) National Geophysical Data Center (NGDC), include He i synoptic charts and coronal hole contours superimposed on Hα synoptic maps, which support the identification of magnetic polarity and the tracking of coronal holes over multiple solar rotations (Harvey and Sheeley 1977). Additional synoptic observations of the He i 10830 Å line were carried out at several observatories, for example at Kharkiv (1992–2000; Belkina et al. 1996), Norikura (1991–1998; Hanaoka et al. 2020), the Crimean Astrophysical Observatory (1999–2018; Andreeva and Malashchuk 2024), SOLIS/VSM (2003–2013; Keller et al. 2003), NSO Full Disk Patrol (FDP; 2011–2017), ISOON (2003–2012), Mauna Loa observatory with the Chromospheric Helium-I Imaging Photometer (CHIP; 1996–2013),^13^ Yunnan Optical and Near-infrared Solar Eruption Tracer (ONSET; 2011–2018), as well as from Mitaka (since 2010). Mitaka, in particular, since 2010 produces also magnetograms in the He i 10830 Å line (Hanaoka et al. 2015). Figure 12 panels e and f show two exemplary images of He i 10830 Å line observations from Mauna Loa CHIP and Mitaka, respectively.

### Multi-wavelength observation compilations

In this section I discuss secondary products replicating and combining observations over different wavelength intervals that were discussed in the previous sections. These are mostly drawings incorporating information from various types of observations, but also compilations of photographic copies. Although the use of primary observations is ideally preferred for most purposes, I discuss these secondary products here as they can still provide important insights: on the one hand, they allow for rapid visualisation of information from different atmospheric layers; on the other hand, they may preserve information for days for which the original observations used to create them are damaged, destroyed, or of unknown whereabouts.

**Fig. 13:**
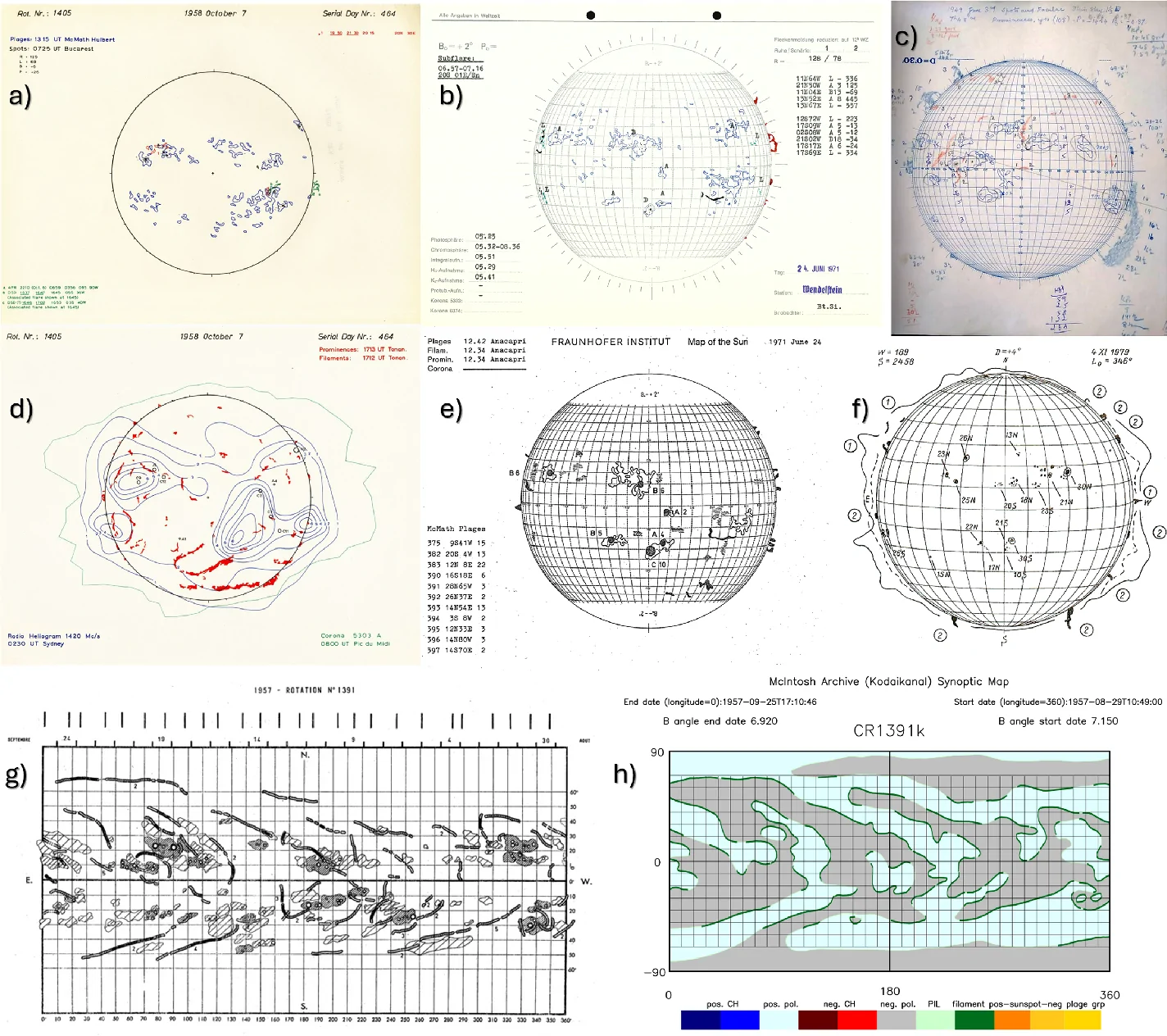
Examples of drawings of multi-wavelength observations. (**a** and **d**) The two types of drawings for IGY (7 October 1958), (**b**) Wendelstein (24 June 1971), (**c**) Kodaikanal (3 June 1949), (**e**) Fraunhofer institute (24 June 1971), (**f**) Kislovodsk (4 November 1979), (**g**–**h**) Meudon and McIntosh synoptic charts (rotation 1391: 29 August to 24 September 1957), respectively

#### Kodaikanal drawings

The Kodaikanal suncharts (Ravindra et al. 2020; Priyadarshi et al. 2023) are coloured drawings of solar features made on sheets with a Stonyhurst latitude-longitude grid (5$^\circ$ spacing) etched into them. An example of Kodaikanal sunchart is shown in Fig. 13c). The drawings were produced from Kodaikanal photographic plates or films over the period 1904–2020. Each chart combines multiple features: sunspots from white-light observations, filaments from Hα observations, and plage and prominences from Ca ii K observations. Distinct colours were employed to indicate features; sunspots in black, filaments in red, and plage and prominences in blue. Additional metadata, including the solar north position angle (P-angle), the heliographic latitude of disc centre ($B_0$ angle), and the observation time, appear at the top of each chart. When Kodaikanal observations were unavailable, data from Meudon or Mount Wilson were used instead; in such cases, the features were drawn in alternate colours (e.g., filaments in green rather than red) to distinguish them from the Kodaikanal records.

#### Meudon synoptic charts

In 1919, d’Azambuja started the production of synoptic charts as Carrington maps combining chromospheric plage and filaments with photospheric sunspots (Mouradian 1998; Malherbe 2023). An example of Meudon synoptic chart is shown in Fig. 13g. The maps were constructed using Ca ii K and Hα spectroheliograms from Meudon to identify plage and filaments, and observations at the Ca ii K wing (termed K1 at Meudon although they were not centred at the K1 minimum; see Fig. 6) for sunspots. Although, according to Mouradian (1998) only the maps starting in 1946 included information from Hα observations. For each synodic solar rotation (mean duration 27.2753 days), the average positions of solar structures were projected onto rectangular maps with longitude on the x-axis and latitude on the y-axis. The collection spans rotation 876 (March–April 1919) through rotation 2008 (October 2003).^14^ Each map was accompanied by tables listing parameters such as coordinates, lifetimes, lengths, heights, and areas of the observed features. When Meudon data were unavailable observations from Kodaikanal, Mount Wilson, and Coimbra were used.

#### Wendelstein drawings

Daily solar feature drawings are available for the years 1947–1987 from the Wendelstein observatory.^15^ These detailed illustrations provide a composite view of various solar phenomena, including coronal intensity, Ca ii K plage, Hα filaments and prominences, as well as sunspots. An example of Wendelstein full-disc drawing is shown in Fig. 13b. A Stonyhurst disc with the corresponding $B_0$ angle is used, and observation times are indicated on the left. On the disc filaments and sunspots are drawn in black, while Ca ii K plage borders in blue. There are also markings in green, which appear to be white-light faculae, but these are more unclear. At the limb, prominences are shown in red, while coronal intensities are annotated numerically every 5$^\circ$ around the solar disc. Solar flare events are also listed, including their start and end times and positions; underlined times denote high confidence, while non-underlined times remain uncertain. To the right of each drawing, the positions and Zürich classes of sunspots are listed.

The data were contributed by many observatories worldwide. For instance, during April–June 1969, Hα images were provided by Anacapri, Athens, Burbank, Catania, Freiburg, Haleakalā, Kodaikanal, Sacramento Peak, Tehran, Tonantzintla, and Wendelstein. Ca ii K images were contributed by Anacapri, Arcetri, Catania, Kodaikanal, Manila, Rome, and Wendelstein, while coronal 5303 Å observations came from Norikura, Pic du Midi, Sacramento Peak, and Wendelstein.

#### Fraunhofer Institute maps

The Fraunhofer Institute produced drawings that compiled and standardised information from multiple types of solar observations obtained at various international observatories, covering the period 1956–1973 (See Footnote 15). They used symbols, hatching, and annotations to represent phenomena like sunspots, plage, and flares. An example of Fraunhofer institute full-disc drawing is shown in Fig. 13e. Sunspots are represented as circles of varying sizes: small for types A, B, J; medium for C, D, G, H; large for E, F (classified per Zürich scale). Circle centres mark the group’s centre of gravity at 12 h UT. Annotations include group type and spot count (e.g., “E 34”). Sunspots were identified with data from Athens, Potsdam, Freiburg, Capri, Helwan, Kandili, Kanzelhöhe, and Wendelstein. Small, one-day-only A-groups, were ignored if they were observed by just one site.

Plage regions are drawn from Ca ii K spectroheliograms in three classes: (1) Shredded and weak: hatched, (2) Continuous: bordered, and (3) Continuous and bright: bordered and hatched. However, it is important to note that this is not a photometric scale. They used data from Schauinsland, Kodaikanal, Mitaka (drawings), Wendelstein, Arcetri, and Crimea, while any further gaps were filled by using Meudon synoptic charts if available.

Filaments, Prominences, and sudden filament disappearances (Dispartitions Brusques; DBs) were extracted from Hα observations. Filaments and prominences drawn at their 12 h UT positions, while in cases that there were significant changes within 24 h they included supplementary drawings with the source observatory and time noted. For DBs they indicated the starting and ending time intervals. For the filament detection they used Hα observations from Crimea, Capri, Schauinsland, Kanzelhöhe, Kodaikanal, Harestua, Sacramento Peak, Sydney, Brussels, Wendelstein, Honolulu, Kandili, and Tonanzintla, while prominences were derived from Kanzellhöhe, Ondrejov, and Potsdam observations.

Coronal brightness at Fe XIV 5303 Å was occasionally provided at the solar limb on a five-step scale (11–30, 31–55, 56–85, 86–120, and >120, in units of $10^{-6}$ of the Sun’s brightness), along with information on flare positions. These compilations drew on coronal observations from the High Altitude Observatory, Climax, Norikura, Pic du Midi, Sacramento Peak, Wendelstein, and Kanzelhöhe sites. Flare information were provided from Burbank, Climax, Huancayo, McMath-Hulbert, Meudon, Mount Wilson, Nizamiah, Ottawa, Russian observatories, Stockholm, Anacapri, Sydney, Utrecht, Zürich, Arosa, and Locarno observatories.

#### International geophysical year (IGY) maps

During the IGY a coordinated effort was made to document solar activity through two complementary daily map series15, referred to as D-1 and D-2, covering the period between July 1957 and December 1958 (Ellison 1961a). Examples of the IGY D-1 and D-2 maps are shown in Fig. 13a and d, respectively.

The D-1 maps combined observations of sunspot groups (drawn in black), Ca ii K plage (drawn in blue), Hα flares (drawn in red), surges and active prominences (drawn in green). Each type of feature was compiled by a different group: sunspots by the World Data Centre in Zürich, Ca ii K plage by Arcetri, Hα flares by Meudon, and prominences by Boulder. It is important to note that in the D-1 drawings, sunspots and Ca ii K plage are shown at their respective observing times, whereas all other features are adjusted so that their positions are correct relative to the sunspots. For these maps a very wide network of observatories supplied material. For sunspot observations data from Zürich and Locarno were predominantly used, while other European stations were used to fill gaps. Ellison (1961a) noted that sunspots were presented in a simplified manner in cases that their structure was very complicated. For Ca ii K plage observations, important contributions came from Arcetri, Crimea, McMath-Hulbert, and Kodaikanal, while gaps were filled with data from Schauinsland, Ikomasan, Meudon, Mount Wilson, Mitaka, and Wendelstein. According to Ellison (1961a) when different observers traced plage regions from the same image, the resulting areas agreed within about 10%, but discrepancies grew when data from different observatories were compared, which led them to restrict the number of contributing sites to 4 main and 6 secondary (as listed above). For detecting flares and prominences they used copies of Hα patrol photographs from at least 26 stations including Abastumani, Arcetri, Capetown, Capri (two sites one operated by Fraunhofer institute and the other by Stockholm observatory), Climax, Crimea, Haute-Provence, Hertsmonceux, Honolulu, Kiev, Kodaikanal, Lockheed, McMath-Hulbert, Meudon, Mitaka, Ottawa, Sacramento Peak, Sydney, Tonantzintla, Uccle, Ussurisk, Utrecht, and Wendelstein.

The D-2 maps were designed to complement the D-1 series by focusing on systematic, multi-wavelength tracings of the Sun (Ellison 1961b). They contained four main elements: 1) sunspot group drawings (in black), 2) prominences, filaments, and sudden disappearances (in red), 3) green coronal intensity plots of the Fe XIV 5303 Å line (in green), and 4) radioheliograms at 21 cm (in blue). Different groups provided information about the different features: sunspots, prominences, filaments and sudden disappearances by Fraunhofer institute, green coronal line intensities by High altitude observatory in Boulder, radioheliograms at 21 cm by Commonwealth Scientific and Industrial Research Organisation (CSIRO) in Sydney. It is important to note that in the D-2 drawings, sunspots are plotted at their positions at 12 UT, while all other features are shown at their respective observing times. Sunspot groups were represented by circles whose size reflected their Zürich classification (the same way as in Fraunhofer institute drawings; Sect. 2.5.4 and Fig. 13e, and the data were compiled from several contributing stations including Anacapri, Freiburg (Schauinsland), Kandili, Kanzelhöhe, Potsdam, and Wendelstein. Prominences and filaments were drawn from daily Hα photographs obtained at an extensive international network: Anacapri, Cape of Good Hope, Climax, Crimea, Freiburg, Herstmonceux, Honolulu, Istanbul, Kanzelhöhe, Kodaikanal, McMath-Hulbert, Meudon, Ondřejov, Potsdam, Sacramento Peak, Sydney, Tonantzintla, Uccle, Washington Naval Observatory, Wendelstein, and Mount Wilson. Particular emphasis was placed on sudden disappearances of long-lived filaments and prominences, whose timings were reconstructed from patrol films and supplementary reports supplied by Meudon, Boulder, Cape of Good Hope, Dunsink, Herstmonceux, Kanzelhöhe, Kodaikanal, McMath-Hulbert, Oslo, Sacramento Peak, Sydney, and Wendelstein. The green coronal line intensities at 5303 Å were measured around the globe at nine stations: Pic du Midi, Arosa, Kanzelhöhe, Wendelstein, Sacramento Peak, Climax, Alma Ata, Kislovodsk, and Norikura. Each used different methods and scales, but they were converted to a common intensity scale, adopting Pic du Midi as the reference. These standardized data provided a daily record of coronal brightness at intervals of 5$^\circ$ around the solar limb. Finally, the Sydney Radiophysics Laboratory contributed radioheliograms of the Sun at 21 cm wavelength, obtained with a Mills Cross radio interferometer (Christiansen et al. 1957) and presented as contour plots of brightness temperature.

#### McIntosh synoptic maps

McIntosh (1964) started the production of synoptic charts which continues to this day. These are Carrington maps combining information about Hα filaments and the associated polarity inversion lines (McIntosh 1972).^16^ Up to 1989, only Hα data from Boulder and Sacramento Peak were used; in later periods they also used data from Meudon, Big Bear, Kanzelhohe, Learmonth, Hida, Hiraiso, Culgoora, MLSO, Yunan, Huairu, Teide, Holloman, Ramey, Coimbra, Pic Du Midi, Cerro Tololo, and Udaipur.^17^ Magnetograms from Kitt Peak and Mount Wilson, and later from SoHO/MDI, the GONG network, SOLIS, and SDO/HMI, were used to extract information on regional magnetic polarity. Since 1978, these maps have included information on coronal holes derived from He i 10830 Å observations from Kitt Peak (Webb et al. 2017). From 1996 onward, He i 10830 Å data from MLSO were also incorporated, followed later by observations from SOLIS. More recently, SoHO Extreme ultraviolet Imaging Telescope (EIT; Delaboudinière et al. 1995), SDO Atmospheric Imaging Assembly (AIA; Lemen et al. 2012), and STEREO He ii 304 Å observations have been used as well. The initial collection by McIntosh (1964) covered the period since 1954, while it was recently updated and extended back to 1954 with Hα data from Kodaikanal and reconstructed magnetograms by Virtanen et al. (2019). Figure 13h shows an example map from the McIntosh archive.

#### Kislovodsk maps

Since 1979, the Kislovodsk Observatory has also produced synoptic drawings by combining information from various types of observations. These were based on data collected at multiple sites across the former Soviet Union and include sunspots (including their magnetic field measurements), filaments, prominences, plage regions, and coronal emission. An example drawing from Kislovodsk Observatory is shown in Fig. 13f.

The Kislovodsk maps were recently updated and became available in a digital platform^18^ (Tlatova et al. 2018). The maps include sunspots identified from digitized photoheliograph observations at the RGO and Kislovodsk observatories (Tlatov et al. 2016b); Ca ii K plage regions observed at the Kodaikanal, Mount Wilson, Sacramento Peak, and Kislovodsk observatories (derived by Tlatov et al. 2009); filaments based on observations from the Kodaikanal and Kislovodsk Observatories (Tlatova et al. 2017); prominences from the “Memorie della Società degli Spettroscopisti Italiani” and Kislovodsk data (Tlatova and Nagnibeda 2017); coronal emission observed in the 5303 Å and 6374 Å lines from Sacramento Peak, Lomnický štít, and Kislovodsk data (Aliev et al. 2017); and coronal hole boundaries from He i 10830 Å line data from Kitt Peak as well as extreme utraviolet (EUV) 195 Å data from SoHO/EIT (Tlatov et al. 2014).

**Fig. 14:**
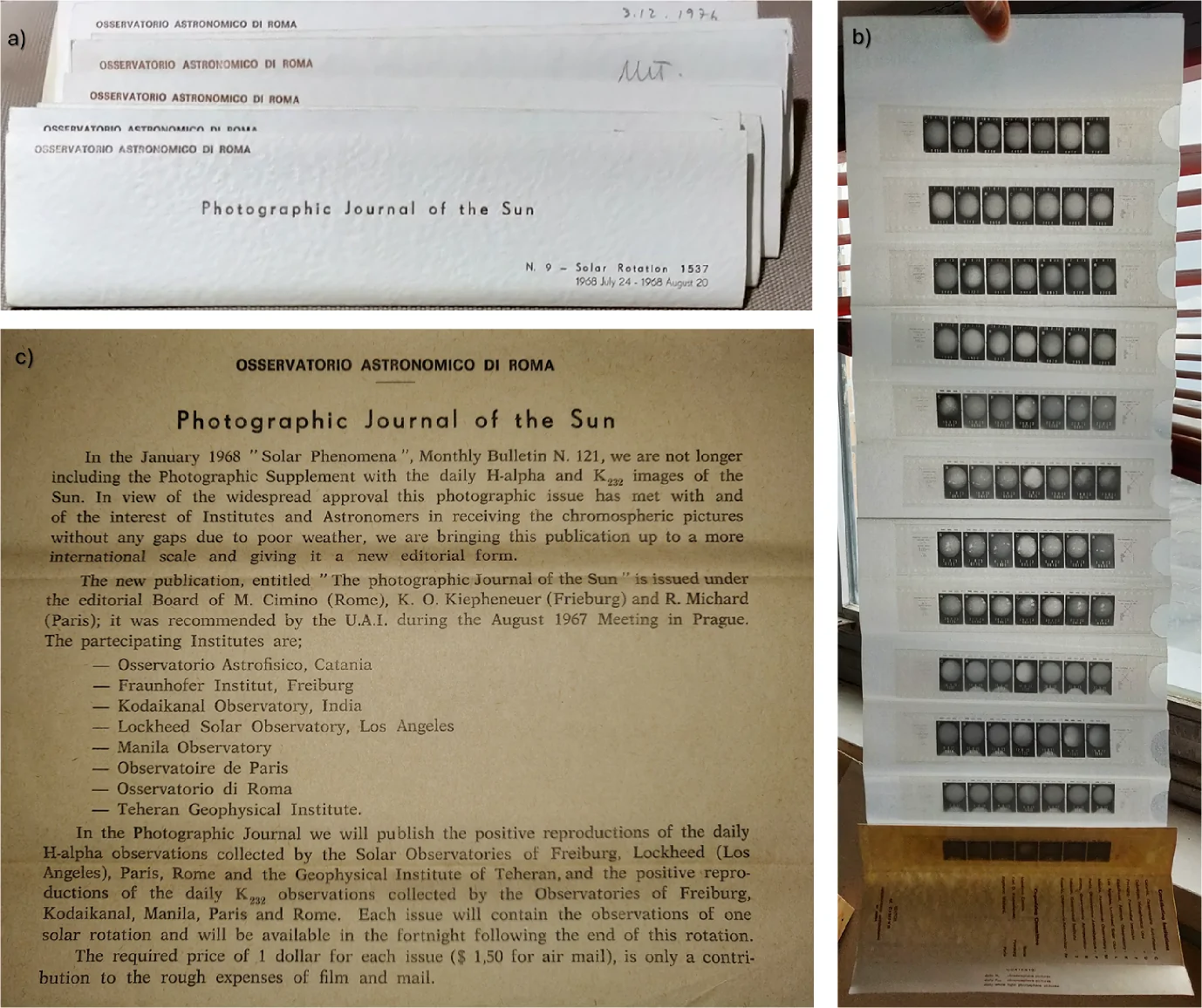
Examples of the Photographic Journal of the Sun (Sect. 2.5.8). Panel (**a**) shows the folders enclosing the films, panel (**b**) the contents of the folders, and panel (**c**) a note announcing the production of the Photographic Journal of the Sun from its first issue. K$_{232}$ refer to Ca ii K observations, while H-alpha to Hα

#### Photographic journal of the Sun

The Photographic journal of the Sun was produced as supplementary material to the monthly bulletin of the observatory of Rome over the period 1967–1978 (Cimino 1969; Chatzistergos et al. 2020c). It included 35 mm celluloid films with positive photographic copies of Ca ii K , Hα , and white-light full disc observations covering four weeks in each set. Rome filtergrams made the basis of this journal, but gaps were covered from various other observatories. These include Catania, Debrecen, Kodaikanal, Lockheed solar observatory, Manila, Meudon, Tehran, and Wendelstein. Figure 14 shows examples of the Photographic journal of the Sun along with a note introducing the Photographic Journal of the Sun from its first issue.

We note that the quality of these photographs is not optimal, as they are small-format (35 mm film) copies of the original observations. Ideally, data digitised directly from the original observations at these sites should be preferred. However, the Photographic Journal of the Sun includes observations from sites for which the condition or availability of the original material is currently uncertain. This is the case, for instance, for the Manila, Tehran, Lockheed Solar Observatory, and Wendelstein observations. We further note that Chatzistergos et al. (2020c) used these journals to digitise all available Ca ii K observations from Manila and Catania, which at present constitute their only available digital versions. However, none of the Hα or white-light observations from these sources have been digitised.

### Image processing methods

In the previous sections we discussed the available historical and modern observations. The availability of such data in digital format allows for analyses from scratch from those data. That includes both to reanalyse historical data so that the desired metrics can be derived in a consistent manner based on modern standards as well as analysing data that were not used for this purpose before. Here I give a brief overview of key processing that is important for photographic as well as pictorial archives.

#### Limb detection and image standardisation

Identifying the solar disc in the images is one of the initial steps in data processing. This step is particularly critical for photographic data, as accurate disc identification is necessary for applying limb darkening correction. However, it is also essential for hand-drawn data when extracting the positions and areas of solar features. The detection of the solar disc is often done manually by selecting three to five points along the solar limb using a computer mouse (e.g., Arlt et al. 2013; Priyal et al. 2014b; Ermolli et al. 2023). Most studies based on photographic data rely on automated methods, typically employing edge detection techniques such as the Sobel filter, Canny edge detector, or Hough transform (e.g., Veronig et al. 2000; Zharkova et al. 2003; Ermolli et al. 2009a; Chatterjee et al. 2016; Chatzistergos et al. 2016, 2020c; Pötzi et al. 2021).

The studies also differ in their treatment of the solar limb, with some assuming a perfectly circular shape and others fitting an ellipse. Although the true solar disc is expected to be circular, several non-solar factors cause the disc in the recorded images to deviate from this ideal shape and appear more elliptical or distorted. General sources of ellipticity affecting most datasets include atmospheric diffraction (Corbard et al. 2019), misalignment between the optical axis and the camera during the observation as well as during the digitisation of the photographic plates. In addition, spectroheliograms are particularly susceptible to solar disc distortions caused by uneven motion of the scanning instrument, leading to differential stretching of strips across the solar disc (See Sect. 2.1.2).

These effects have been found to result in solar disc ellipticities of typically around 0.05 for filtergrams taken with CCD cameras (e.g., Rome/PSPT; Chatzistergos et al. 2020b). For spectroheliograms stored on photographic plates, ellipticities generally range between 0.1 and 0.2, though exceptional cases have been recorded. For example, early Sacramento Peak data show ellipticities up to 0.4 (Chatzistergos et al. 2020b), while isolated early Kenwood and Kyoto observations exhibit ellipticities as high as 0.8 (Chatzistergos et al. 2020c).

Correcting for solar disc ellipticity and distortion is therefore crucial, although not always applied. A common approach involves fitting an ellipse to the solar limb and then resampling the image so that the semi-major and semi-minor axes are equal, effectively restoring the solar disc to a circular shape (Zharkova et al. 2003; Tlatov et al. 2009; Chatzistergos et al. 2020b, c). This approach assumes an equal image distortion over the solar disc. Improving on this would need identifying how such distortions occur over the solar disc and thus disentangling the various potential contributing factors. This is very challenging, and at the moment no method has addressed this. As a result, the process of accounting for the disc ellipticity inherently carries some uncertainty. Due to this, many studies of solar photographic archives did not account for the disc ellipticity (e.g. Bertello et al. 2010; Priyal et al. 2014b; Chatterjee et al. 2016; Singh et al. 2023).

Image distortions exist on drawings too. This can arise due to the way the drawing was made, but also due to the digitisation. The former affects mostly direct observations and not those made by projecting the Sun on a piece of paper. This distortion is not possible to be corrected, thus deriving locations and areas of the drawn features can only be very rough for drawings that were made with a direct observation and not projection. Distortions due to the digitisation can occur in cases where the surface of the paper was no properly flattened for the scanning. This is not uncommon for old documents that are very sensitive. Such distortions, if they are not extreme, have the potential of being corrected, although not in a straightforward way.

#### Photometric calibration

**Fig. 15:**
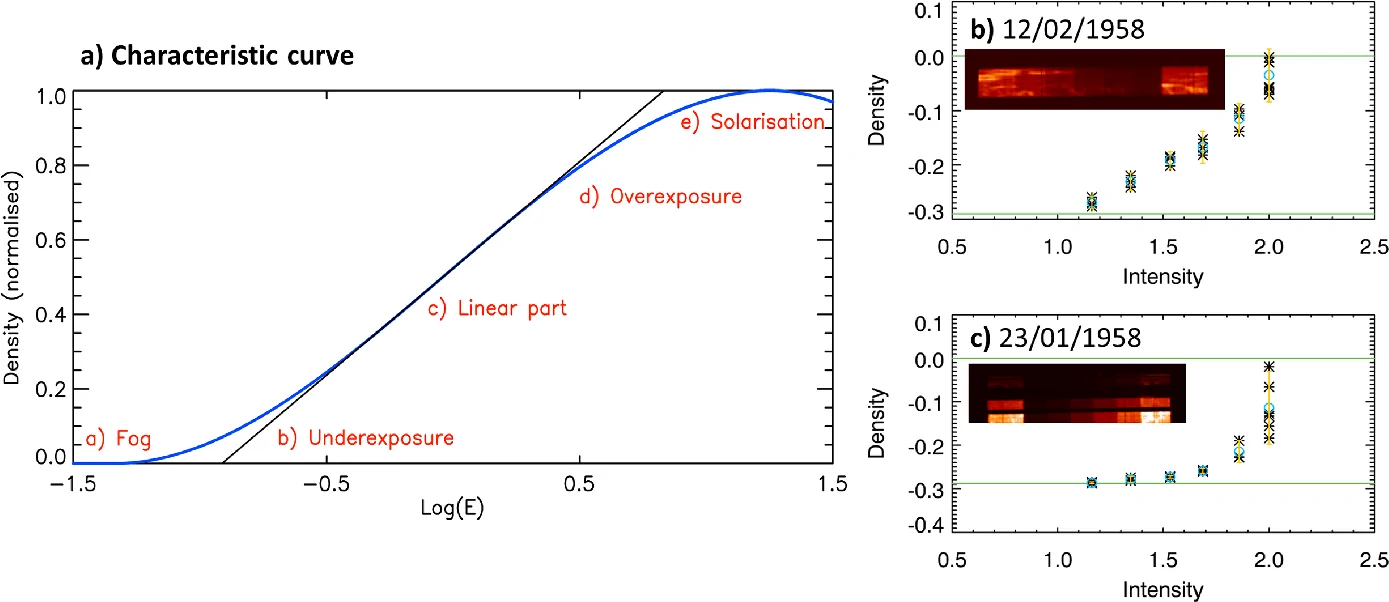
Panel (**a**): Standard shape of characteristic curve. Panels (**b**–**c**); Examples of calibration wedges from Arcetri Ca ii K observations and the corresponding characteristic curves derived from them

Unlike modern digital detectors, which typically exhibit a linear response to incident light, photographic emulsions display a non-linear sensitivity (Mees 1942; James and Higgins 1968; Dainty and Shaw 1974). This non-linearity presents a major challenge for the analysis of historical data, as even small changes in observational conditions can result in significant, non-linear variations in recorded brightness. To derive accurate results from such historical photographic observations, particularly for studies of brightness of different features, it is necessary to account for the non-linear response of the photographic plates. This process is called photometric calibration. This involves constructing a calibration curve, commonly referred to as a characteristic curve (Hurter and Driffield 1890), that describes the photographic emulsion’s non-linear response to light. The characteristic curve, *f* relates the measured values from the photographs, expressed as density *d*, to the logarithm of the exposure, *E*, where exposure is defined as the product of intensity, *I* and exposure time, *t*:

1 $$\begin{aligned} d=f(\textrm{log}(It)). \end{aligned}$$

Assuming a constant exposure across the photographic plate, *t* gets absorbed as a constant in *f* and the relation simplifies to:

2 $$\begin{aligned} d=f(\textrm{log}I). \end{aligned}$$

Characteristic curves typically exhibit a sigmoid shape (Fig. 15). The curve’s lower and upper extremes correspond to underexposed and overexposed regions, respectively, while the central, approximately linear portion represents the properly exposed areas. High-quality observations ideally fall within this linear range. Numerous factors influence the shape of the characteristic curve, including the exposure time, observational conditions (such as temperature and humidity), the chemical composition of the emulsion, development process parameters, the wavelength of observation, storage conditions, and the digitisation procedure. However, these variables are rarely fully documented for historical data to allow determining the form of the characteristic curve based on them, while it is almost impossible to have accurate information for deterioration due to storage. As a result, alternative methods are needed to estimate the characteristic curve for effective photometric calibration.

Here it is important to make a distinction between filtergram and spectroheliogram observations. Since filtergrams are single-exposure photographs of the full solar disc, the exposure time is indeed constant over the entire image allowing for the reasonable assumption that a single characteristic curve applies uniformly across the entire image. This is not necessarily the case for spectroheliograms, however, where the images are constructed by sequentially scanning the solar disc in narrow strips, effectively creating a mosaic from multiple exposures taken over an extended period (typically of a few minutes). As a result, variations in observational conditions, such as changes in seeing, illumination, or instrument stability, can occur during the scan. In extreme cases, these changes can lead to significant differences in the characteristic curve between individual strips within the same spectroheliogram. However, even for spectroheliograms, in general it is reasonable to assume a uniform exposure time across the entire solar disc, provided there were no issues with instrument motion or drastic changes in observing conditions.

The earliest method for photometric calibration involved the use of calibration wedges (Fig. 15). These wedges were created by exposing a portion of the unexposed photographic plate to a series of known light intensities. By measuring the resulting optical density for each exposure, one could approximate the characteristic curve of the plate. However, this approach has several important limitations. Most notably, the vast majority of historical observations lack dedicated calibration wedges. For instance, calibration wedges are only available for Arcetri data after 22 February 1938 (Ermolli et al. 2009a), for Mount Wilson data after 9 October 1961, and for Kodaikanal data between 1958 and 2006 (Priyal et al. 2014b). Additional issues with the wedge method include potential mismatches between the density range covered by the calibration wedge and that of the solar image, and insufficient sampling of the characteristic curve. Moreover, repeated exposures, intended to be identical, sometimes yielded noticeably different density values (e.g. Fig. 15c), further complicating calibration and introducing significant uncertainty (see also Foukal et al. 2009). Despite these limitations, Fredga (1971); Kariyappa and Sivaraman (1994); Worden et al. (1998a); Giorgi et al. (2005); Ermolli et al. (2009a) made use of the calibration wedges, which however limited their studies to the periods that wedges are available. Furthermore, studies such as Steinegger et al. (1996b) and Priyal et al. (2014b) made use of the available calibration wedges from Sacramento Peak and Kodaikanal to derive average characteristic curves, which they then applied uniformly across the respective datasets. While this method provided a first-order correction, it introduced additional uncertainties, since the calibration curve is expected to change between observing days owing to varying observational conditions.

Different methods to perform the photometric calibration on individual observations were developed over the last years. In particular, Ermolli et al. (2009b) calibrated Arcetri plates based on the approach by Mickaelian et al. (2007), which was originally devised for calibrating star survey plates. The calibration is done with the following formula:

3 $$\begin{aligned} I_i=\frac{V-B}{T_i-B} \end{aligned}$$

where $I_i$ and $T_i$ are the intensity and the transparency of the *i*th pixel, *V* the average value in an unexposed section of the photographic plate, and *B* is the mean value of the darkest exposed regions. Tlatov et al. (2009) also applied a linear calibration to photographic Ca ii K data. They derived a scaling by relating standard centre-to-limb variation (CLV) profiles by Pierce and Slaughter (1977) to the measured plate density at two μ (the cosine of the heliocentric angle) positions (μ=0 and μ=0.9).

Chatzistergos et al. (2018b) developed a method that uses the quiet Sun **CLV** from modern, high-quality observations as a reference (Rome/PSPT for Ca ii K and Catania for Hα ). By comparing this reference CLV with that measured in historical data, Chatzistergos et al. (2018b) were able to infer the characteristic curve. A similar calibration process was also used by Kakuwa and Ueno (2021). Extensive validation with synthetic data demonstrated that the method by Chatzistergos et al. (2018b) can recover intensity values with a mean error below 1%, outperforming all previously proposed methods (Chatzistergos et al. 2018b, 2019b). This method of photometric calibration was applied on both Ca ii K (Chatzistergos et al. 2020c) and Hα data (Chatzistergos et al. 2023a).

#### Limb darkening and artefact compensation

Limb darkening is the optical effect whereby the intensity of solar radiation observed from the disc centre gradually diminishes toward the limb. This phenomenon is prominent in photospheric and chromospheric observations and arises due to the vertical temperature gradient in the solar atmosphere. Specifically, light seen at the disc centre originates from deeper, hotter layers, than light seen near the limb, which is emitted by higher, cooler layers, leading to the apparent dimming. In the corona, the situation is reversed, with limb brightening observed instead. However, this is not discussed here further, since there are no such coronal observations covered in this review.

Many applications require, or are simplified by, compensating for solar limb darkening so that the solar disc has a uniform intensity scale. This correction enables direct comparisons of brightness levels between different regions on the disc. Moreover, solar observations are often affected by various artefacts such as vignetting, uneven illumination, and calibration issues, which must also be corrected to obtain scientifically reliable data.

Here I describe some of the most commonly used processing techniques developed to compensate for limb darkening and to mitigate image artefacts. I list methods irrespectively of the spectral line that they were optimised to work on. Thus in the following it lists methods that were developed to work on Ca ii K , Hα , white-light, as well as 1600 Å.

The available correction techniques can be broadly classified into five categories:

- One-dimensional (1D) fitting to the average radial intensity profile;
- Two-dimensional (2D) fitting across the entire image;
- 1D fits along image rows and columns;
- 2D running-window median filtering;
- Hybrid methods combining multiple of the above approaches.

**Fig. 16:**
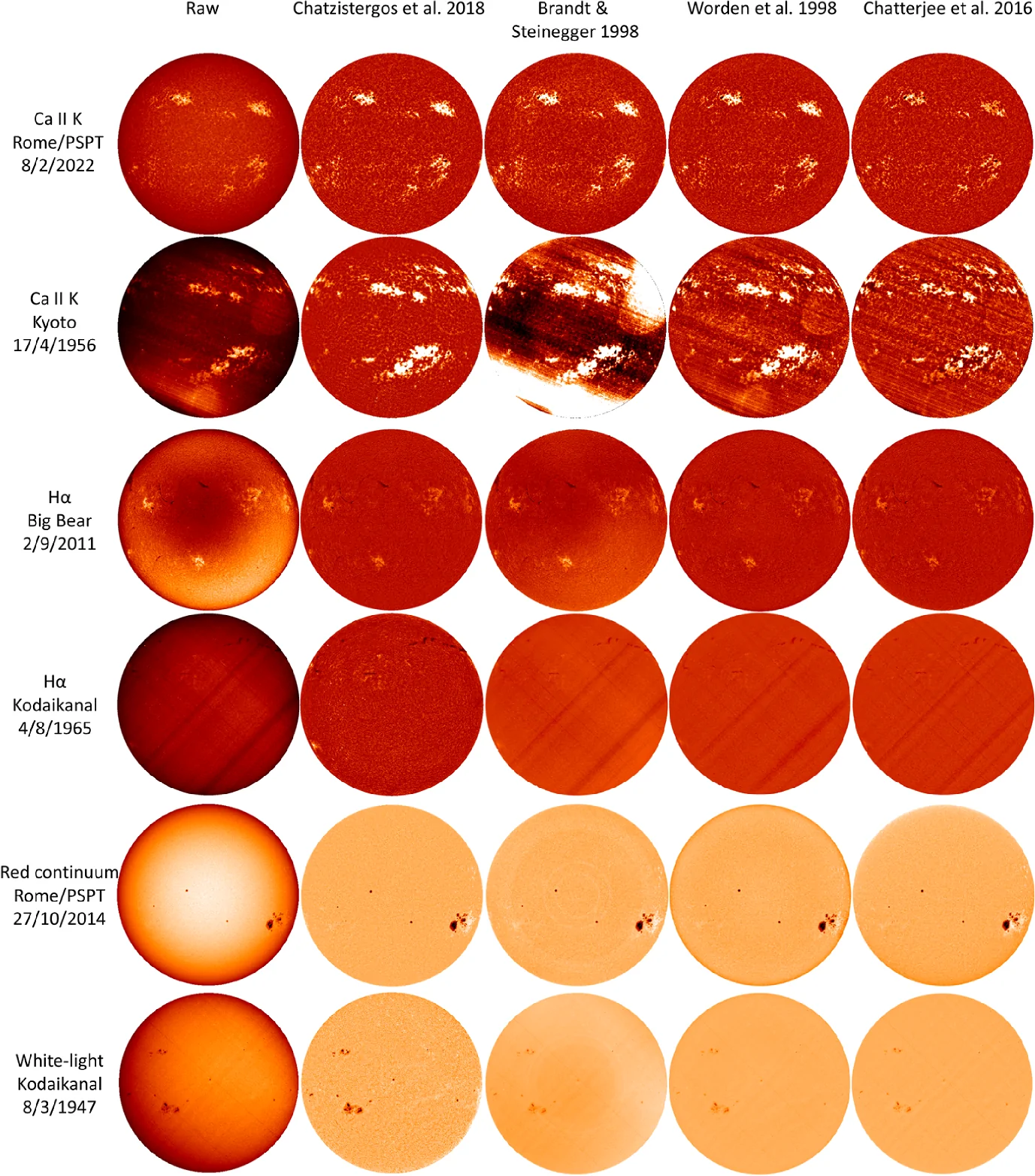
Example images of processing the raw observations shown in the 1st column with various methods from the literature

Examples of processing historical and modern Ca ii K , Hα , white-light, and continuum observations with selected methods from all categories (except the 2nd one) are shown in Fig. 16.

The first category assumes radial symmetry of the solar disc and involves fitting a polynomial, typically of 4th or 5th order, to the mean radial intensity profile as a function of μ. This method has been widely used in earlier studies (e.g., Brandt and Steinegger 1998; Walton et al. 1998; Johannesson et al. 1998; Denker et al. 1999; Zharkova et al. 2003; Yeo et al. 2013; Diercke and Denker 2019; Pötzi et al. 2021). Figure 16 shows examples of processed images with the method by Brandt and Steinegger (1998) from this category. The assumption of radial symmetry is very reasonable for space-based observations (as used by Yeo et al. 2013), provided the images do not suffer from other observational or calibration issues that can introduce large-scale artefacts (e.g. ghost image). However, ground-based observations typically deviate from a radially symmetric limb darkening profile due to observational imperfections. This is particularly pronounced in historical photographic and SHG data (See Fig. 16 rows 2–4). As a result, this method is recommended only for space-based observations with minimal image artefacts, where its simplicity offers a practical advantage over more sophisticated approaches. Its application to ground-based historical archives, however, is strongly discouraged.

An alternative is to fit a 2D polynomial surface (usually of 4th order) to the entire image (Caccin et al. 1997, 1998b; Worden et al. 1998b). This method does not assume radial symmetry and can model large-scale patterns. However, it lacks robustness against small-scale artefacts and localised distortions that are common in older data. It particularly fares poorly in accounting intensity variations across linear segments which are rather common in SHG data, and thus its use is not advised for historical data either.

A more adaptive approach involves fitting 1D polynomials separately to the rows and columns of the image (Worden et al. 1998a). Worden et al. (1998a) used 5th-order polynomials, while Priyal et al. (2014b) employed 3rd-order fits. Figure 16 shows examples of processed images with the method by Worden et al. (1998a) from this category. This technique better accommodates asymmetric features, but its effectiveness is limited by the varying μ-range across different scan lines, particularly near the limb. To partly address this, Worden et al. (1998a) repeated the procedure after rotating the image by 45$^\circ$, although this introduces additional noise. This method works well to account for artefacts caused by step changes in raster exposure in spectroheliograms provided they align well with the direction the fits are applied (See 2nd and 4th row in Fig. 16). Thus, this was the first method that performed reasonably on historical observations although its performance is reduced near the limb.

A more widely used and flexible technique for limb darkening correction is the application of a 2D running-window median filter (Lefebvre et al. 2005; Bertello et al. 2010; Chatterjee et al. 2016; Bose and Nagaraju 2018; Bertello et al. 2020; Tähtinen et al. 2022). Figure 16 shows examples of processed images with the method by Chatterjee et al. (2016) from this category. An advantage of this approach compared to the previous ones is that it makes no assumption about the functional form of the limb darkening. For each pixel, it assigns the median value within a surrounding square window, thereby also helping suppress large-scale artefacts. The window size is a critical parameter: smaller windows preserve more local detail but may be influenced by bright features, while larger windows better average out such features but may over-smooth the background. For example, Bose and Nagaraju (2018) used a window of size ∼R/2, whereas Chatterjee et al. (2016) opted for ∼R/6. Chatzistergos et al. (2018b) showed that the optimal window size for Ca ii K data lies between *R*/8 and *R*/6, a result later corroborated for AIA 1600 Å data by Tähtinen et al. (2022). Despite its versatility, the median filter method presents several challenges: near the solar limb, the filter includes off-disc pixels, which skews the estimated background toward lower values. This method poses an important limitation on how to exclude bright regions, and as a result the presence of bright active regions leads to biases in the background estimate, causing over-correction of such regions (see decreased contrast in plage regions in 2nd row of Fig. 16). This can also lead to artificial features such as dark halos surrounding active regions. Sharp artefacts, such as those caused by step changes in raster exposure, are not well handled (see 2nd and 4th rows of Fig. 16). Thus, the issues of this method limit its effectiveness when applied alone, for both historical and modern data.

Given the limitations of individual techniques, numerous studies have employed combinations of these methods (e.g., Centrone et al. 2005; Ermolli et al. 2009b; Singh et al. 2008; Priyal et al. 2014b, 2017, 2019; Chatzistergos et al. 2016, 2018b; Singh et al. 2022; Tähtinen et al. 2022). For instance Diercke et al. (2022); Dineva et al. (2022) used an approach from the first group to derive an initial estimate followed by Zernike polynomial fitting to capture remaining asymmetries. Among the various approaches, the method introduced by Chatzistergos et al. (2018b) is noteworthy. The method builds upon and refines several existing techniques to overcome the limitations of earlier approaches. In particular, it employs an iterative procedure that combines running-window median filtering with polynomial fitting across image rows and columns. This allows it to effectively address common artefacts along image lines in historical data and accurately exclude bright plage regions prior to applying the running-window median filtering. Figure 16 shows examples of processed images with the method by Chatzistergos et al. (2018b) from this category. Notably, the method by Chatzistergos et al. (2018b) remains the only method to date that has been systematically validated across a wide range of datasets. Initial tests using synthetic Ca ii K data demonstrated its superior performance compared to other published methods, and besides Ca ii K data (Chatzistergos et al. 2020c) it has since been successfully applied to both Hα (Chatzistergos et al. 2023a) and continuum observations (Chatzistergos et al. 2020a; Ermolli et al. 2022a).

Figure 16 illustrates the performance of representative correction methods from all groups except the second one. In particular, the comparison includes the methods by Brandt and Steinegger (1998, 1st group), Worden et al. (1998a, 3rd group), Chatterjee et al. (2016, 4th group), and Chatzistergos et al. (2018b, 5th group). Each method was applied to one white-light, one red-continuum, two Ca ii K , and two Hα observations. Although we note that among these methods only the one by Chatzistergos et al. (2018b) has been used in the literature on data other than Ca ii K . While all methods perform adequately on artefact-free CCD data (rows 1 and 5), residual defects remain in some cases (notice variations near the limb in row 5). On historical images with significant artefacts (rows 2 and 4), the method by Chatzistergos et al. (2018b) demonstrates superior performance, particularly in preserving plage contrast and accounting for the large scale artefacts.

#### Segmentation

For many applications that we will discuss below, it is needed to apply a segmentation approach to identify a specific activity feature of interest. This was historically done manually on drawings or physical photographs. Thus, the identification of features in photographic plates or drawings was largely subjective and performed by eye, while simple measuring tools or transparencies were used to estimate the areas and positions of these features. These transparencies contained circles of known area, allowing direct comparison with the feature of interest to estimate its size, while other transparencies, bearing coordinate grids appropriate for the time of year of the specific observation, were used to determine heliographic latitude and longitude.

Although digital images allow for more objective identification of features of interest, a range of practical complications initially limited the reliability of automated methods, and early studies with digital images therefore continued to rely on manual feature detection (e.g. Foukal 1996). This has changed over the recent years with the development of various automatic segmentation approaches for digital images. One class of methods involves applying morphological operators directly to images without prior preprocessing (e.g. Barata et al. 2018), such as limb-darkening correction. However, this approach has only been applied to modern digital images and it has not been applied to digitised historical photographic data. It is likely that its performance would be limited for such material, which is often affected by strong artefacts that would first need to be properly accounted for.

However, the vast majority of segmentation approaches are typically applied only after preprocessing has been completed, yielding flat, contrast images corrected for limb darkening. Various segmentation approaches have been applied, typically employing both contrast and size thresholds. The size threshold is generally used to exclude small regions that were falsely identified as the feature of interest. Four main strategies have been used to define the contrast threshold, although this list is not exhaustive: (1) a fixed threshold value (e.g. Priyal et al. 2014b), (2) a multiplicative factor to the standard deviation of the overall image contrast values (e.g. Chatterjee et al. 2016), (3) a multiplicative factor to the standard deviation of the contrast values of quiet-Sun regions only (e.g. Chatzistergos et al. 2020c), and (4) a multiple level tracking method (e.g. Bovelet and Wiehr 2001; Chatzistergos et al. 2023a). However, we note that the performance of all of these segmentation methods relies on accurate preprocessing to account for limb darkening and large scale artefacts. Furthermore, the segmentation approach of using a multiplicative factor to the standard deviation of the overall image contrast values is expected to introduce a activity-dependent bias in the results (see e.g., Chatzistergos 2017).

## Indices of solar activity and variability

There are numerous indices of solar activity and variability, which cannot all be covered here. In this section, we discuss some of the most important ones, with particular emphasis on indices derived from historical observations as well as on important long-term disc-integrated measurements. The indices are presented according to the solar feature they describe. In particular, we discuss metrics about sunspot**s** (Sect. 3.1), photospheric faculae (Sect. 3.2), chromospheric plage (Sect. 3.3), chromospheric network (Sect. 3.4), filaments (Sect. 3.5), prominences (Sect. 3.6), and flares (Sect. 3.7) followed by the disc-integrated measurements of chromospheric emission (Sect. 3.8), green coronal emission (Sect. 3.9), radio fluxes (Sect. 3.10), and solar irradiance (Sect. 3.11).

### Sunspots

**Fig. 17:**
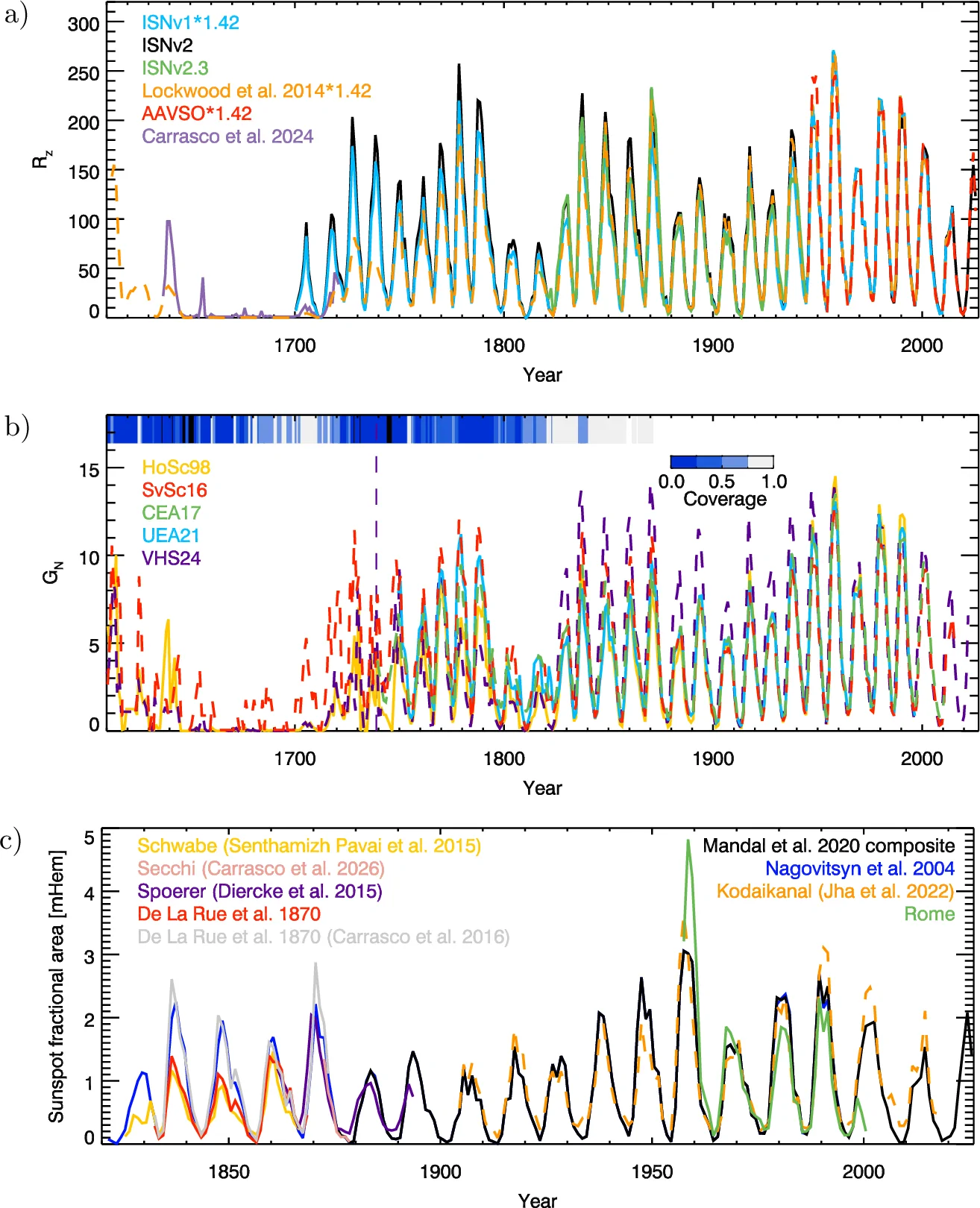
Panel (**a**): Sunspot number series. The series have been scaled as denoted in the legend to match ISNv2 after 1947. Panel (**b**) Group sunspot number series. At the top of the panel it also shows the fraction of days within each year with at least one record in the Vaquero et al. (2016) database. Coverage is colour-coded, with complete coverage shown in white, the absence of observations in black, and intermediate values divided into four ranges, as indicated by the colour bar within the panel. The vertical dashed purple line marks the year 1739 over which there is no observer with observations before and after. Panel (**c**): Series of sunspot areas. The areas are corrected for foreshortening, except those from Secchi which are projected areas in units of 8 arcsecond$^2$ here divided by 60 to roughly match the areas of Spörer. In all panels the values shown are annual means

Sunspots are dark features in the solar surface and they are the most observed aspect of solar magnetic activity (Solanki 2003). Various characteristics of sunspots have been recorded since the early 1600 s from white-light and continuum observations (Sect. 2.2), which were obtained either visually or photographically. Most surviving white-light observations include or at least allow the identification and counting of sunspot groups. In addition, some archives provide the number of individual sunspots, measurements of group or spot areas (whether individual, daily totals, or even separated into umbral and penumbral components), and positional information for each group.

Records of sunspot characteristics appear in two main forms: material produced directly by the observers (typically as annotations or tabulated data) (e.g. Carrasco et al. 2021c, 2026; Hayakawa et al. 2022) and values extracted later from (re-)analyses of drawings or photographs (e.g. Neuhäuser et al. 2015; Svalgaard 2016; Bhattacharya et al. 2021; Vokhmyanin and Zolotova 2023). Unfortunately, no standardized protocol for recording sunspot characteristics existed among observers. Thus, material produced directly by the observers are highly diverse and even individual observers sometimes changed their practices over time (see e.g. Ermolli et al. 2023). Such records are sometimes accompanied by a drawing or photograph of the Sun, which allows for future reanalysis, thus resulting in multiple series of sunspot characteristics from the same source observations. Thus, when using such raw sunspot data, it is important to know not only the source of the observations but also the study that extracted the sunspot characteristics. Key advantages of (re-)analysing drawings and photographic records is that these materials can be reinterpreted according to modern classification standards (e.g. Svalgaard 2016; Bhattacharya et al. 2021; Vokhmyanin and Zolotova 2023), something that is not feasible with textual descriptions or historical tabulations. In addition, they allow researchers to extract characteristics that may not have been reported in the original analyses. It is important to note that there is still significant work needed to recover potentially overlooked historical records of sunspots.

In the following sections, I discuss the three principal series of solar activity derived from sunspot observations: sunspot number (Sect. 3.1.2), group sunspot number (GSN; Sect. 3.1.3), and sunspot areas (Sect. 3.1.4) starting first with a short description of the databases of raw sunspot counts (Sect. 3.1.1). The reader can find more in depth information about sunspot data from individual observers, and especially drawings, in the reviews by Vaquero et al. (2016); Arlt and Vaquero (2020); Clette et al. (2023).

#### Raw sunspot number databases

Wolf (1850) initiated a process of collecting records of individual sunspots and sunspot groups from different observers to create the first such database of raw counts. This database was produced at Zürich observatory until 1980 and continues to be maintained today by the Sunspot Index and Long-term Solar Observations (SILSO) program at the Royal Observatory of Belgium (ROB) and is the basis for the sunspot number series. However, despite the availability of recent data in digital format, portions of the historical record have yet to be digitized. Work to complete this digitisation is currently in progress.

Hoyt and Schatten (1998, HoSc98, hereafter), building on the database by Wolf (1850) and incorporating additional sources, created a new database of sunspot groups only. Focusing on sunspot groups allowed the early data over the 1600 s, which did not include accurate information on individual sunspots, to be incorporated too. A great advantage of this database was that it was presented in a digital format, which allows for easier evaluation and use for sunspot series recalibrations. This database was subsequently updated by Vaquero et al. (2016) who incorporated records from more observers as well as corrected problems in the existing entries. The Vaquero et al. (2016) database employs a versioning scheme with periodic updates; the present version is 1.21. In particular, significant effort has been invested over the last decades to improve the database of raw sunspot group counts by locating and digitizing such records in order to verify and correct the digital versions (e.g. Vaquero et al. 2016; Aparicio et al. 2018; Carrasco and Vaquero 2016; Carrasco et al. 2021c, 2024). Overall, the Vaquero et al. (2016) database includes data from more than 700 professional and amateur observers recording information about sunspots since the early 1600 s. Figure 17b includes a colour-coded histogram at the top of the panel, which denotes the temporal coverage (fraction of days within a year with at least one observation) in the version 1.21 of the Vaquero et al. (2016) database. This reveals that there is a complete coverage since the 1870 s, but the coverage consistently drops for earlier times reaching particularly low values in the mid 1700 s, where there are years without any observation. It is important to stress here that there is currently no observer with records spanning both before and after 1739 (marked with vertical dashed line in Fig. 17b). As we will see in the following sections, this poses a significant obstacle in sunspot number (re-)calibrations over that period. The scarcity of sunspot records in the 17th and 18th centuries underscore the importance of identifying and digitizing previously unexamined archives.

#### Sunspot number series

The sunspot number series, first introduced by Wolf (1850) at Zürich observatory, is the most commonly used metric of solar activity. Since 1980 it is curated by SILSO at ROB. The sunspot number series, $R_z$, combines the information from both individual sunspots and groups based on the formula

4 $$\begin{aligned} R_z=k(10G+S), \end{aligned}$$

where *G* is the number of groups, *S* is the number of individual sunspots, and *k* is a scaling factor used to normalise the data of individual observers to a reference level. When Wolf (1850) first introduced this series, he set the reference level based on his own observations. This dataset is now commonly referred to as the International Sunspot Number version (ISNv) 1. ISNv1 provides daily values from 1818 onward. For earlier periods, due to the scarcity of observations, only monthly values are available back to 1749, and annual averages extend as far back as 1700, though these earliest data carry considerable uncertainty.

Subsequent analyses revealed several inconsistencies in ISNv1, prompting the release of ISNv2 (Clette and Lefèvre 2016; Clette et al. 2023). Notable issues included the so-called “Waldmeier jump” around 1947 (estimated inflation of ISNv1 by ∼1.177 after 1947; Clette and Lefèvre 2016; Clette et al. 2021), inconsistencies in the scaling between Schwabe’s and Wolf’s records during 1849–1863 (Clette et al. 2014; Bhattacharya et al. 2023), and the “Locarno drift” beginning in the 1980 s (Clette et al. 2016). Since the raw observational counts of all contributors were not yet fully digitised by 2016, ISNv2 accounted for these issues (except for the “Locarno drift”) by adjusting ISNv1 rather than implementing a full recalibration from original raw data. A significant change introduced in ISNv2 was the adoption of Wolfer as the reference observer instead of Wolf. This adjustment led to ISNv2 values being 1.67 times higher than those of ISNv1 (although note that the correction for the “Waldmeier jump” slightly lowers this factor for the period after 1947). Since then, further updates have been made, involving partial recalibrations from the available raw data over limited periods. In particular, ISNv2.1 to ISNv2.3 (Bhattacharya et al. 2023, 2024) include recalibrations of the sunspot number series over the period 1816–-1944 at daily resolution. We note that the linear scaling for cross-calibrating the sunspot number records of different observers has been criticised by Lockwood et al. (2016c). Notably, ISNv2.3 is the first sunspot number series that was produced with the non-liner non-parametric cross-calibration approach by Chatzistergos et al. (2017, see Sect. 3.1.3) which was previously used for GSN series. Figure 17a compares ISNv1 (scaled by 1.95 to match ISNv2 after 1947), ISNv2, and ISNv2.3. We note that ISNv1 has been discontinued, ISNv2.1−2.3 were standalone releases without further updates, while ISNv2 receives regular updates.^19^ A complete recalibration of the entire record, requiring the full digitisation of the remaining historical data, and the release of ISNv3 is planned for the second half of 2027.

Lockwood et al. (2014) applied different corrections to ISNv1 than those used for ISNv2. In particular, Lockwood et al. (2014, 2016d, 2016a) independently estimated the correction for the discontinuity in ISNv1 over 1945 (note that for ISNv2 this was applied over 1947) to be ∼1.12. Lockwood et al. (2014) also scaled the HoSc98 GSN series (see Sect. 3.1.3) to extend their sunspot number series back to 1610. Note, however, that the HoSc98 GSN series has significant uncertainties in the pre-1739 period (see Sect. 3.1.3) and as a consequence the same stands for the Lockwood et al. (2014) sunspot number series. The Lockwood et al. (2014) sunspot number series is also shown in Fig. 17a.

Vaquero et al. (2015) developed an empirical relationship between the active-day fraction, calculated from all available raw observer counts over a year, and the sunspot number series, providing a method to estimate annual sunspot numbers during periods of very sparse observations. Carrasco et al. (2022a, 2024, 2025) updated and applied this approach to estimate annual sunspot number over 1635–1726 (Fig. 17a). Moreover, Carrasco et al. (2024) reported a slower recovery to typical levels of solar activity in the early 1700 s than currently indicated in both ISNv1 and ISNv2. These findings are consistent with the Lockwood et al. (2014) sunspot number as well as with reconstructions based on $^{14}$C data by Usoskin et al. (2021).

An alternative sunspot number series exists by the American Association of Variable Star Observers (AAVSO; Schaefer 1997).^20^ This series was established in 1944, prompted by the challenges of receiving timely ISNv1 updates from Zürich during the Second World War. the AAVSO sunspot number series covers the period since 1945 only and it is also produced with a linear scaling approach. This series is incorporating data from more than 100 amateur and professional observers and receives regular updates. This series is also shown in Fig. 17a.

#### Group sunspot number (GSN) series

The GSN series considers only the number of sunspot groups, and was first introduced by HoSc98. The HoSc98 GSN series used the RGO record as the reference and linearly scaled all other observers’ records to match it. However, several issues have since been identified with the HoSc98 GSN, particularly regarding its cross-calibration methodology (e.g. Lockwood et al. 2016c; Usoskin et al. 2016a), as well as inconsistencies in the scaling of observers that paradoxically made the counts of earlier observers with inferior telescopes to be treated as more accurate than those from modern ones (Clette et al. 2023). These shortcomings led to the development of several alternative GSN series, each employing a different cross-calibration technique (e.g., Svalgaard and Schatten 2016; Cliver and Ling 2016; Usoskin et al. 2016b, 2021; Chatzistergos et al. 2017; Willamo et al. 2017; Velasco Herrera et al. 2024).

Among these, Svalgaard and Schatten (2016, SvSc16, hereafter) and Cliver and Ling (2016) retained a linear scaling approach but introduced important improvements over HoSc98. Specifically, Cliver and Ling (2016) accounted for issues in the early RGO data (e.g., Clette et al. 2014; Lockwood et al. 2016b). Meanwhile, SvSc16 employed a backbone methodology to link observers, thereby reducing the number of calibration steps compared to the multi-step daisy-chain approach used by HoSc98. Velasco Herrera et al. (2024, VHS24, hereafter) likewise applied a linear scaling approach, taking the Kislovodsk record as the reference. However, this approach suffers from serious methodological limitations because it is applied to records without direct overlap, implicitly assuming that the standard deviation of the counts remains constant over time (see also Chatzistergos et al. 2025b). More significant advances were achieved by Usoskin et al. (2016b; 2021), Willamo et al. (2017; 2018, UEA21, hereafter, for its last version by Usoskin et al. 2021) and Chatzistergos et al. (2017, CEA17, hereafter), who developed non-linear, non-parametric cross-calibration methods that also provide formal error estimates. A key distinction between these approaches lies in how they link different observers. In particular, the method of CEA17 requires direct temporal overlap between the records of the observers being cross-calibrated. In contrast, the method by UEA21 does not require direct overlap, instead uses active-day fraction statistics to build a calibration matrix between observers.

We note that the GSN series differ also in their resolution, with HoSc98 and CEA17 having daily values, UEA21 monthly, while SvSc16 has only annual values. Figure 17b shows the main GSN series. The figure shows that most GSN series are in reasonable agreement from around 1880 onward but diverge significantly in earlier periods. In particular, HoSc98 and SvSc16 suggest the lowest and highest level of activity, respectively (see Lockwood et al. 2016b; Clette et al. 2023). Various factors can contribute to these discrepancies, and understanding their origins is important for accurately reconstructing past solar variability. Different studies have sought to understand how various factors have affected the cross-calibration of past sunspot records. For instance, in his effort to recount the sunspot records made by Staudach and to cross-calibrate them to modern levels, Svalgaard (2016) enlisted the help of the Antique Telescope Society to use telescopes similar to those employed by Staudach.^21^ Karachik et al. (2019) degraded SDO/HMI continuum observations to study the effect of telescope aperture and stray-light on sunspot, group number, and sunspot area and found that sunspot number series is more severely affected by these. Chatzistergos et al. (2025b) conducted the most extensive evaluation to date of the various cross-calibration methods, using synthetic data based on RGO records. Their analysis revealed that linear approaches tend to overestimate cycle maxima during periods of strong activity and underestimate them during weaker cycles, thus introducing a bias in secular trends. In contrast, non-parametric methods produced more consistent and accurate results, with generally lower errors for typical characteristics of historical observer records. Among the non-parametric approaches, the method by CEA17, which relies on direct overlap between observers, performed best. Another way to assess the quality of the existing GSN series is by comparing them with reconstructions based on cosmogenic isotopes. These comparisons argue against the low activity of HoSc98 and the high activity of SvSc16 (e.g. Muscheler et al. 2016; Asvestari et al. 2017; Usoskin et al. 2021), instead supporting as more realistic the reconstructions of CEA17 and UEA21.

Notably, none of the existing GSN series extends to the present or is currently updated with new data. That is due to the Vaquero et al. (2016) database of raw group counts not extending beyond 2010. Thus, the CEA17 and UEA21 GSN series cover the periods 1739–2010 and 1749–2010, respectively, while the HoSc98 series covers the period 1610–1995. SvSc16 incorporated data beyond those in the Vaquero et al. (2016) database, with coverage spanning 1610–2015. Because observations in the 1700 s are sparse and no observer’s record spans both before and after 1739 (see Fig. 17), current calibration methods fail over this period. HoSc98 and SvSc16 used alternative, poorly constrained assumptions to calibrate the pre-1739 data, making the resulting series highly uncertain. The approach of Carrasco et al. (2022a, 2024, 2025) provides a means to assess the GSN series over this period more realistically, albeit at an annual resolution.

Overall, the CEA17 and UEA21 series are currently recommended as the most realistic GSN reconstructions. For the period prior to 1739, however, all GSN series should be treated with caution. The reconstruction by Carrasco et al. (2022a, 2024, 2025) likely provides the best current estimate covering the Maunder Minimum and its surrounding period, while HoSc98 can be considered a lower-bound estimate for the Maunder Minimum. An updated GSN series is expected to be produced along with ISNv3.

**Fig. 18:**
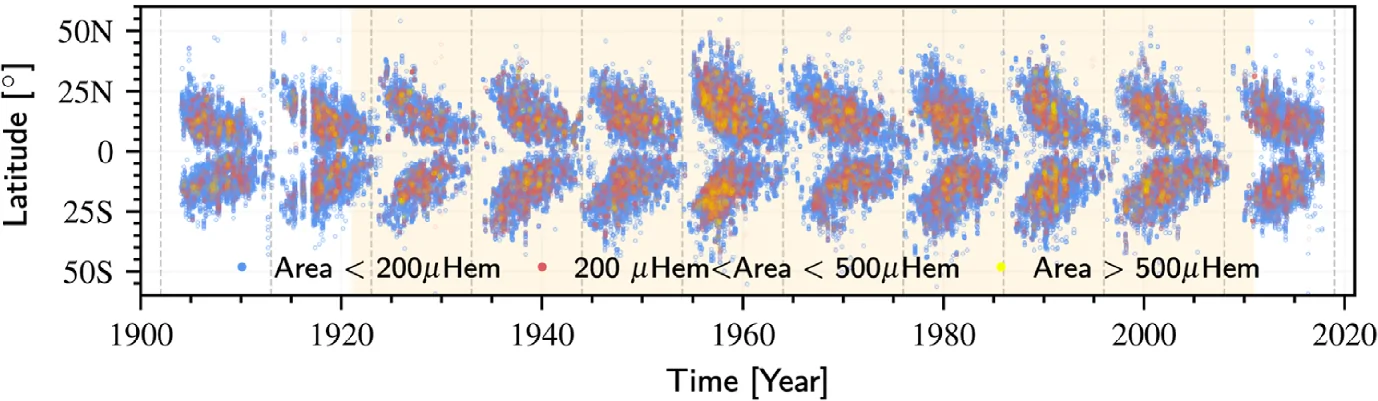
Kodaikanal sunspot butterfly diagram. Taken from Jha et al. (2022)

#### Sunspot areas

The available data also carry extensive information about sunspot areas. That includes information about the area of individual groups as well as disc-integrated values. Such records extend back to the early 1600 s, but they are rather sporadic and rare prior to the late 19th century. From the 1870 s onwards, several observatories began systematically deriving sunspot areas, including RGO, Coimbra (Carrasco et al. 2018), Madrid (Aparicio et al. 2014), Rome (Guglielmino et al. 2018), Catania (Godoli et al. 1971), and Ebro (de Paula et al. 2022). These records often show significant discrepancies, attributable to differences in measurement techniques and observer acuity (see Karachik et al. 2019). As an example, the Rome series is shown in Fig. 17c.

A particularly important and widely used dataset of sunspot areas comes from the RGO, covering the period from 1874 to 1976. This archive includes areas of individual sunspot groups derived by observers at RGO from photographic white-light observations (Sect. 2.2.1). To achieve nearly complete daily coverage, RGO supplemented its own data with observations from Cape of Good Hope, Dehra Dun, Kodaikanal, the Royal Alfred Observatory (Mauritius), Harvard College Observatory, Melbourne Observatory, Mount Wilson Observatory, and the US Naval Observatory. The consistent methodology to derive sunspot group areas applied across data from different sites improved the internal consistency of the dataset. However, variations between different contributing observatories and the evolving photographic processes of the time may have further influenced the data quality. In 1976, the RGO program ended and it was succeeded by Debrecen (Györi et al. 2002), using mostly observations from Gyula, Debrecen, and SDO/HMI, as well as several other observatories to fill temporal gaps. Debrecen also provided the areas of individual sunspots along with the areas of groups. The Debrecen sunspot database was also discontinued in 2018. United States Air Force (USAF) Solar Observing Optical Network (SOON) is another program of such sunspot observations producing a dataset of sunspot areas (Giersch et al. 2018). The USAF/SOON network comprises solar observatories distributed worldwide to provide continuous, 24-hour synoptic monitoring of sunspots since 1976. USAF/SOON sunspot areas have been reported to be underestimated by a factor of 1.1 to 1.9 compared to other archives such as Debrecen, while this factor depends on the distance of the sunspot group from the disc centre and time (Baranyi 2018; Giersch et al. 2018). More recently, Meadows (2025) derived sunspot areas from SDO/HMI and SoHO/MDI data covering the period since 1996.

As we discussed in Sect. 2.2 there are many white-light and continuum photographic archives over the last two centuries. The recent interest in digitising such data has led to studies that derive sunspot areas from these data. For instance, Mandal and Banerjee (2016) and Jha et al. (2022) reanalysed the recently digitised white-light photographs from the Kodaikanal Observatory to construct a consistent time series of sunspot areas. This series is shown in Fig. 17c. Reanalyses of individual archives play a vital role in addressing problems present in existing data. Moreover, as the RGO database combines observations from various sources (including Kodaikanal), they can also reveal possible discrepancies among those records.

Besides sunspot areas obtained from photographic observations, historical sunspot drawings provide records reaching as far back as the early 1600 s, albeit with few observations before the 1800 s. For instance, Secchi reported sunspot areas averaged over a solar rotation for 1871–1876 in his book “Le Soleil” (Secchi 1875; Carrasco et al. 2021b). More extended information about sunspot areas was provided in Secchi’s notebooks which were recently digitised by Ermolli et al. (2023) and the information about sunspot areas (disc-integrated and for individual groups) was recovered by Carrasco et al. (2026). In recent years, several studies have recovered original observer notebooks with sunspot drawings, allowing the extraction of sunspot areas from them. Noteworthy examples include drawings by Schwabe (1825–1867; Senthamizh Pavai et al. 2015), Spörer (1861–1884; Diercke et al. 2015), and additional shorter-term datasets over earlier periods analysed by Neuhäuser et al. (2015); Hayakawa et al. (2018a); Vokhmyanin and Zolotova (2018b, 2018a); Vokhmyanin et al. (2021); Vokhmyanin and Zolotova (2023); Carrasco et al. (2020); Zolotova and Vokhmyanin (2021). It is important to note that, Schwabe’s drawings include only the umbral areas, omitting the penumbra. The sunspot areas from Schwabe (Senthamizh Pavai et al. 2015) are shown in Fig. 17c too, after converting them to approximate total spot areas by multiplying the umbral area by a factor of five, assuming a fixed penumbra-to-umbra ratio of 4 (e.g. Brandt et al. 1990; Vaquero et al. 2005; Wenzler et al. 2006; Hathaway 2013; Jha et al. 2019; Chowdhury et al. 2024). Spörer’s drawings were synoptic charts and the areas are given for individual groups (Diercke et al. 2015). One can estimate the disc-integrated daily sunspot areas by integrating the group areas within a roughly 60$^\circ$ longitudinal window. However, this is very approximate, since it does not take into account the evolution of sunspot groups and includes them in the same way over subsequent days. This estimate is also shown in Fig. 17c. The relatively low values in earlier periods are partly attributable to the limited observational acuity of early observers such as Schwabe and Spörer. Overall, caution is warranted when using such sunspot area reconstructions from historical drawings, as they often involve substantial uncertainties. These mainly reflect the observer’s visual acuity, long-term consistency, and drawing style, which can complicate the accurate extraction of sunspot areas. Furthermore, in many cases observers depicted sunspots merely as dots (e.g. Hayakawa et al. 2018b; Carrasco et al. 2021a), which prevents accurate area estimation although derivation of rough locations of spots is still possible. Besides uncertainties inherent to the observations themselves, Sánchez-Bajo et al. (2026) compared various sources of uncertainty in the determination of sunspot areas from digitised drawings and digital images, finding that thresholding and perspective effects were the dominant contributors.

Archives that contain information about the area and location of individual sunspot groups or spots allow the construction of a time-latitude (or butterfly) diagram as well as studying the North–South asymmetry of sunspot areas (e.g. Norton and Gallagher 2009; Hathaway 2015; Mandal et al. 2017b; Jha et al. 2024). Figure 18 shows the sunspot butterfly diagram derived with Kodaikanal white-light data by Jha et al. (2022). See the review by Arlt and Vaquero (2020) for a more detailed overview of existing sunspot time-latitude maps.

Finally, composite series have been created that compile sunspot area records from multiple sources. One of the earliest such attempts was by de La Rue et al. (1870), who combined sunspot area measurements by Heinrich Schwabe (over 1832 to 1853), Richard Christopher Carrington (over 1854 to 1860), and Kew observatory (over 1861 to 1868) derived by them. Vaquero et al. (2002b) by comparing the areas by de La Rue et al. (1870) to ISNv1 and HoSc98 GSN series identified potential issues with the way these data were compiled. Based on this Vaquero et al. (2004); Carrasco et al. (2016) calibrated the de La Rue et al. (1870) sunspot areas to those from RGO and produced a consistent sunspot area series back to 1832. Nagovitsyn et al. (2004) applied a different calibration to the de La Rue et al. (1870) sunspot areas and connected them to RGO and Pulkovo observatory data. More recently, Balmaceda et al. (2009) produced another composite, which was later updated by Mandal et al. (2020), based on photographic solar observations covering the period since 1874. These include the disc-integrated sunspot areas as well as the individual group areas. Mandal et al. (2020) used RGO series as the reference and combined them with Debrecen and Kislovodsk data by applying scalings to account for their different levels. The sunspot area composite by Mandal et al. (2020) is shown in Fig. 17c together with the series by de La Rue et al. (1870), their subsequent corrections by Nagovitsyn et al. (2004) and Carrasco et al. (2016), and additional sunspot area records from Schwabe (Senthamizh Pavai et al. 2015), Secchi (Carrasco et al. 2026), Spörer (Diercke et al. 2015), Kodaikanal (Jha et al. 2022), and Rome.

### Photospheric faculae

**Fig. 19:**
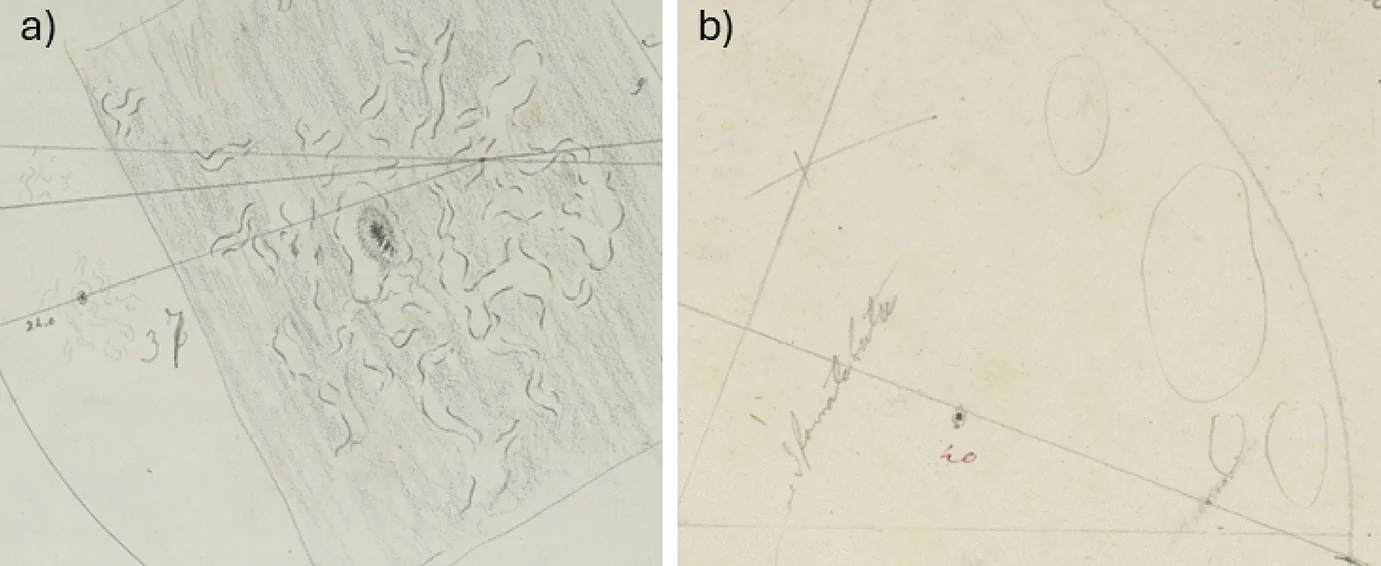
Close-ups of white-light faculae in drawings made by Secchi on 22 June 1866 (**a**) and 19 May 1862 (**b**) (Ermolli et al. 2023)

**Fig. 20:**
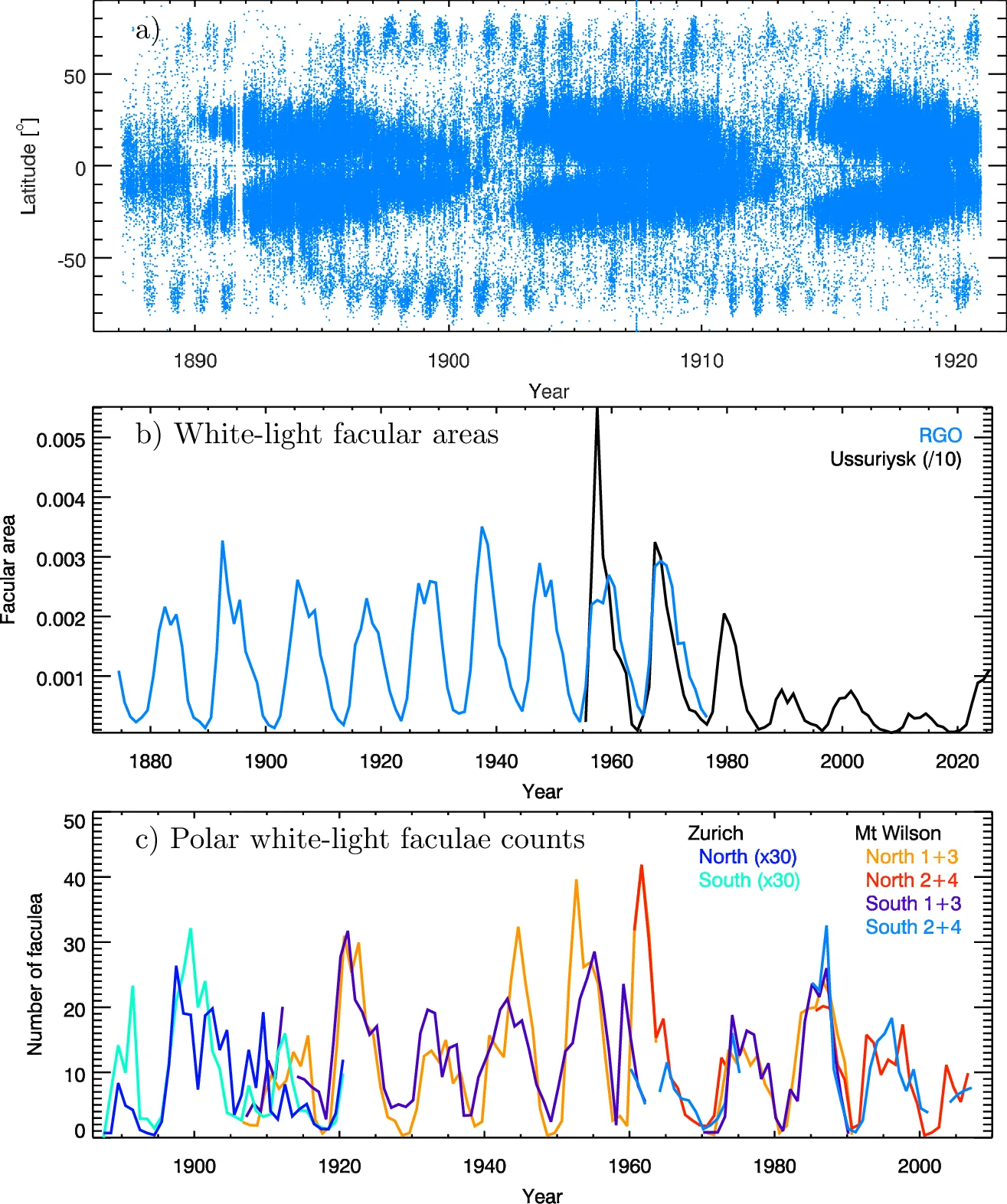
Records of white-light faculae. Panel (**a**): Butterfly diagram of Zürich white-light facular counts. Panel (**b**): Facular fractional areas. The Ussuriysk areas have been divided by 10 to be closer to those from RGO. Panel (**c**): Polar facular counts. Shown are annual values

Faculae are bright features seen in the photosphere and information about them is derived from white-light and narrow-band photospheric observations (Secs. 2.2.1–2.2.2). Although, as we discussed in previous sections, there are white-light drawings from the early 1600 s (e.g. by Scheiner; see Carrasco et al. 2022b), records of faculae from white-light data are significantly more scarce than those of sunspots. This is largely due to the inherent difficulty of detecting faculae in white-light observations, since they are absent near the disc centre and become visible only toward the limb, where they nonetheless exhibit low contrast. Additional complications stem from the complex and irregular morphology of faculae, which makes them more difficult to draw than sunspots. As a consequence, historical drawings of faculae typically depict them in a very rough way (see Fig. 19b, which unfortunately limits potential reanalyses of such drawings to extract meaningful information about white-light data.

Notwithstanding these issues, there are several historical records of white-light faculae that derive from drawings. For instance, Secchi drew white-light faculae in his full-disc observations over 1861–1878 (see Fig. 19), allowing for deriving information of faculae (Ermolli et al. 2023). Secchi did not tabulate areas of white light faculae, however he maintained records of the latitudinal extend of faculae (Carrasco et al. 2021b). Zürich defined individual facular elements and maintained a dataset of their positions and their numbers over 1887–1920 (Illarionov and Arlt 2022). A time-latitude (butterfly) diagram based on the Zürich white-light facular data is shown in Fig. 20a. Similarly, Lyon observatory presented facular counts in five latitudinal bins as well as the disc integrated areas over 1902–1925 (Guillaume 1921). Faculae were also included in Kanzelhöhe white-light drawings since 1944 (Pötzi et al. 2016). However, they were not always drawn consistently and they seem to have been abandoned in 1989 when Kanzelhöhe started performing photographic observations in white-light.

Extracting white-light faculae from analogue photographic data also poses significant challenges due to various artefacts affecting the images. To my knowledge, there has been no automatic extraction of white-light faculae from analogue photographic data, besides manual estimation of their area by the observers. For instance RGO^22^ has the longest such database of facular areas extending from 1874 to 1976. These areas were measured by two observers using a planimeter (Foukal 1993), while the inclusion of polar faculae was not done in the early data. We note, however, that potential inconsistencies in the RGO facular series may have been introduced by discrepancies among the various archives used to fill gaps on days when no RGO observations were available (see Sect. 3.1.4). Ussuriysk observatory has also maintained a record of white-light facular areas since 1955. Figure 20b shows the facular areas from RGO and Ussuriysk observatory. Facular areas show variations in phase with the solar cycle, while the RGO areas are comparable to sunspot areas (Fig. 17). There are significant discrepancies between the RGO and Ussuriysk series, with the latter having about one order of magnitude greater areas than RGO. The facular areas from the RGO record are notable for exhibiting an evolution markedly different from that of sunspot areas. In particular, solar cycle 19 does not appear as the strongest cycle but rather as the weakest since the 1930 s (see e.g. Foukal 1993). The relocation of the observatory to Herstmonceux in 1949, during the declining phase of solar cycle 18, may have contributed to a discontinuity in the facular record, as photospheric faculae are difficult to detect and thus rather sensitive to changes in observing conditions. However, it remains uncertain whether the low facular levels in the RGO dataset during cycle 19 represent a real feature, as chromospheric plage areas do not show a corresponding decrease (see Sect. 3.3). In contrast to the RGO record, the facular areas from Ussuriysk reach a maximum during cycle 19. However, the sharp subsequent decrease in the Ussuriysk series suggests possible consistency issues.

In recent years, digital narrow-band observations have allowed for automatic and thus more objective identification of facular regions. This has enabled detailed studies of photospheric facular contrast as a function of time and position on the solar disc (e.g. from San Fernando data; Ahern and Chapman 2000, from Rome/PSPT data; Centrone and Ermolli 2003; Ermolli et al. 2007, from SDO/HMI data; Yeo et al. 2013; Yeo and Krivova 2019; Criscuoli et al. 2017, or SO/PHI data; Albert et al. 2023).

There are also time series of polar white-light faculae, defined as the number of facular elements located close to the solar poles. Polar faculae are typically identified as features in white-light or narrowband photospheric images at latitudes beyond 70$^\circ$, although no single standard exists, and values as low as 50$^\circ$ have been used in the literature. Weber (1865, 1868) was the first to recognize that polar faculae reach their maximum near sunspot minimum, thereby establishing the basis for using polar faculae as predictors of the next cycle’s sunspot maximum (see review by Petrovay 2020). Since then there have been only a few observatories that maintained records of polar faculae. The most notable series of polar white-light faculae was derived from Mount Wilson data (Sheeley 1991, 2008; Muñoz-Jaramillo et al. 2012). They counted polar faculae separately for the South and North hemisphere only within one-month intervals over 15 February to 15 March and 15 August to 15 of September, respectively. These days were chosen to have the best visibility to the Sun’s poles possible from a ground-based facility. The number of polar faculae was estimated manually, thus being rather subjective. The whole process was performed in four different campaigns: Sheeley (1964, covering the data 1906–1963), Sheeley (1976, covering the data 1960–1973), Sheeley (1991, covering the data 1970–1990), Sheeley (2008, covering the data 1985–2006), although the same process was applied to identify faculae. The records from Lyon observatory (1902–1925) carry information for polar faculae too, as those beyond 40$^\circ$ (Guillaume 1921). The Mitaka Observatory collected polar faculae data over the period 1951–1991, separately in three latitudinal bands: 50–60$^\circ$, 60–70$^\circ$, and 70–90$^\circ$ (Saito and Tanaka 1957; Tanaka 1964; Irie et al. 1993).^23^ Although the Zürich and RGO Observatories did not produce dedicated time series of polar faculae, their records allow such series to be derived. In particular, Zürich reported the positions of individual facular elements, enabling the identification of polar faculae (Illarionov and Arlt 2022), while RGO records for the period 1874–1955 include information on the area and location of facular regions, allowing polar faculae to be inferred (Jones 1955). Figure 20c shows the counts of polar white-light faculae from Mount Wilson, along with that from the Zürich catalogue where I selected the data in the same way as done for the Mount Wilson ones. Makarov et al. (1989); Makarov and Makarova (1996) also manually derived polar faculae counts for the period 1960–1994 using Kislovodsk white-light observations. They obtained monthly counts of polar faculae, without restricting the analysis to the time interval when each pole is best seen, and defined polar faculae as features located beyond 50$^\circ$ latitude. Janssens (2021) presented a series of polar faculae counts covering 1995–2019 from visual observations in white-light. These observations were corrected for seasonal viewing effects and variable atmospheric seeing to produce a long-term dataset of monthly polar faculae counts that characterizes their temporal evolution over nearly 25 years. More recently, automated methods have been used to extract polar faculae from SDO/HMI data over the period 2010–2024 (Hovis-Afflerbach and Pesnell 2022; Reche et al. 2025). In particular, Reche et al. (2025) employed a machine-learning approach to identify polar faculae (defined as those beyond 50$^\circ$ latitude) and derive timeseries of their counts. Their results yielded approximately 160–200 faculae per pole during the 2019 activity minimum, which is an order of magnitude higher than the values from Mount Wilson and Zürich archives (see Fig. 20).

Overall, a wealth of historical white-light faculae records exists, with additional archives likely still being overlooked. There is currently no comprehensive photospheric facular area record, while the existing records exhibit significant disagreements. Furthermore, existing white-light drawings may contain recoverable facular information (e.g. Secchi drawings; Ermolli et al. 2023), highlighting the importance of historical data recovery and their reanalyses to extract such information. A major limitation of these records at the moment is the arbitrariness involved in faculae identification, compounded in the case of polar faculae by the diversity of definitions adopted for the latitude range. However, modern techniques provide a pathway toward standardising this process.

### Chromospheric plage

Plage regions are the chromospheric counterpart of photospheric faculae and appear as bright, extended patches. In contrast to faculae, plage can be readily observed all over the solar surface and not just near the limb. Plage regions have been primarily studied with Ca ii K observations, although they are also visible in other wavelengths sampling the chromosphere, such as Ca ii H , Ca ii infrared, Hα , and Mg ii h and k.

Various observatories over the beginning of the 20th century derived Ca ii K plage areas and presented them in their reports. Such plage areas were estimated from physical photographs or drawings in a manual way (See Sect. 2.6.4), which unfortunately carries significant subjectivity. In particular, such records survive from at least the Coimbra (covering 1929–1944; Carrasco and Vaquero 2022), Ebro (covering 1910–1937; de Paula et al. 2022), Kodaikanal (covering 1905–1977; Kuriyan et al. 1982), and McMath-Hulbert^24^ (hosted at NGDC; also including small subsets of Mount Wilson and Big Bear Ca ii K data; Foukal 1996) observatories. The McMath-Hulbert plage areas were tabulated on Ca ii K drawings^25^ and were likely derived from these drawings rather than directly from the Ca ii K photographs of the same site. It is noteworthy that Ebro observatory also employed a classification scheme (Curto et al. 2016) in which plage regions were grouped into four primary classes according to their morphological appearance: compact (labelled as ‘c’), dispersed (labelled as ‘d’), mixed (labelled as ‘cd’), or unassignable to any of the other three classes. The Quarterly Bulletin on Solar Activity^26^ (QBSA; covering 1917–1944) also included information about Ca ii K plage from a network of observatories (Cambridge, Kodaikanal, Ebro, Meudon, Mount Wilson, Mitaka, Arcetri, Coimbra, Kyoto). However, instead of quantitative plage areas, QBSA reported qualitative estimates of brightness and extent across the solar disc by manually classifying them on a scale from 0 (no plage) to 5 (maximum brightness and size).

The recent availability of digitized Ca ii K observations provided a valuable opportunity to (re-)analyse historical data and extract plage areas. Some of the early analyses, however, could not fully exploit the potential of these data largely because processing the images was computationally demanding at the time, and they still relied on manual detection of plage regions (e.g. Foukal 1996). As we saw in Sect. 2.6, more advanced processing methods were subsequently developed to analyse the images and derive plage areas in a more objective manner. That included historical archives analysed again, or for the first time, as well as modern archives acquired with digital detectors. Various studies focused on deriving plage areas from Ca ii K observations, however not all of the available Ca ii K archives (Sect. 2.3.1) have been used yet. The majority of studies deriving plage areas used Kodaikanal (e.g. Priyal et al. 2014b, 2017; Ermolli et al. 2009b; Tlatov et al. 2009; Singh et al. 2018, 2022; Chatzistergos et al. 2016, 2019b, c; Jha et al. 2024) and/or Mount Wilson (e.g. Foukal 1996, 1998; Ermolli et al. 2009b; Tlatov et al. 2009; Bertello et al. 2010; Chatzistergos et al. 2016, 2019b, 2020c; Singh et al. 2022) Ca ii K data. Fewer studies used also Arcetri (e.g. Ermolli et al. 2009b, a; Chatzistergos et al. 2018a, 2019b, 2020c, Sacramento Peak (e.g. Worden et al. 1998a; Tlatov et al. 2009; Chatzistergos et al. 2020b, c), and Coimbra (e.g. Dorotovic et al. 1996; Barata et al. 2018; Chatzistergos et al. 2016, 2020c) data. Only a few other archives have been analysed; most notably, Chatzistergos et al. (2020c) examined 33 lesser-studied archives to derive plage areas. The photometric calibration was also not applied consistently in all those series. Worden et al. (1998a); Ermolli et al. (2009a) and Priyal et al. (2014b) used the available calibration wedges, while Tlatov et al. (2009) and Chatzistergos et al. (2016, 2018a, 2019c, 2019b, 2020c, 2020b); Jha et al. (2024) photometrically calibrated the images with the methods presented by Tlatov et al. (2009) and Chatzistergos et al. (2018b), respectively. All other plage areas from historical images were derived from photometrically uncalibrated data.

**Fig. 21:**
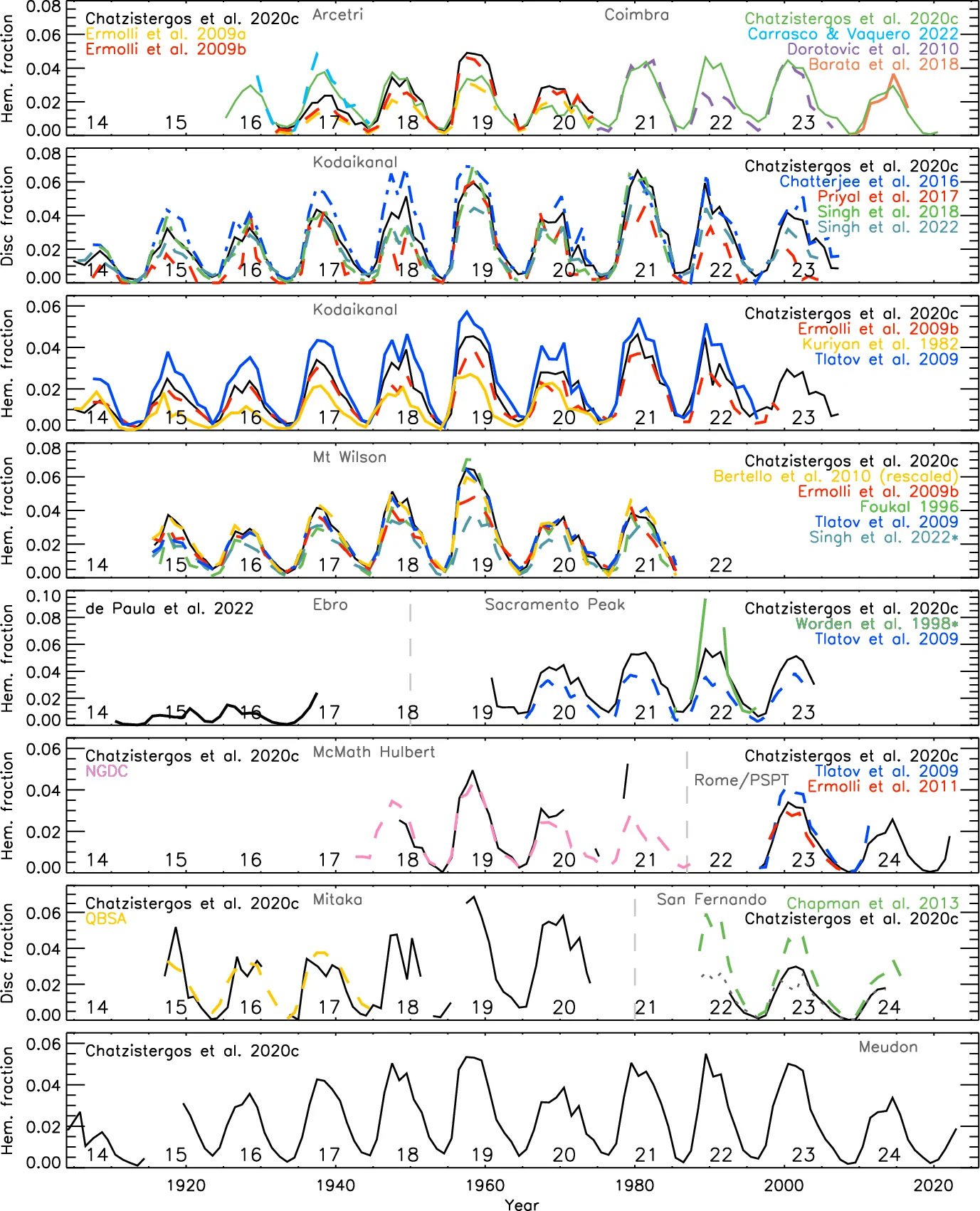
Timeseries of plage areas derived from various Ca ii K  archives (written in grey) and different studies. For the San Fernando series from Chatzistergos et al. (2020c), results from CFDT1 (dotted grey) and CFDT2 (black) datasets are displayed separately. The plots show annual median values. All values are expressed as hemispheric fractions, except for the series in rows 2 and 7, as well as those from Singh et al. (2022, 4th row) and Worden et al. (1998a, 5th row) which are given as disc fraction. The numbers at the bottom of each panel indicate the conventional solar cycle numbering. Vertical lines are used to separate data from different archives in panels that include time series compiled from multiple sources. Adapted from Chatzistergos et al. (2022b)

Overall, plage fractional areas exhibit variations (Fig. 21) that are in phase with the solar cycle, with solar cycle maxima approximately between 0.02 and 0.08. That is about one order of magnitude greater than the sunspot (Fig. 17) or RGO facular areas (Fig. 20b). Figure 21 shows the timeseries of plage areas derived from some of the most important historical Ca ii K archives along with selected modern ones. The figure shows plage areas from different archives in each panel (except for Kodaikanal, which is shown in two panels) and further illustrates the variations in derived plage areas resulting from differences in plage identification. However, caution is warranted, as discrepancies also arise from factors such as data processing, data selection, and the specific digitisation version of each archive. Furthermore, these issues are further exacerbated when comparing plage areas from different archives, because, as discussed in Sect. 2.3.1, the existing Ca ii K archives employed different passbands and therefore sample plage regions at different heights in the solar atmosphere, leading to variations in their observed morphology.

Besides plage areas derived from individual archives, there have also been composite series. The first composite of data from many sources was produced by Chatzistergos et al. (2019b) who combined the data from Kodaikanal, Meudon, Mitaka, Arcetri, Mount Wilson, Rome/PSPT, McMath-Hulbert, Schauinsland, and Wendelstein. To mitigate discrepancies in plage areas caused by the differing bandpasses of the archives, they cross-calibrated the plage areas to a common reference, resulting in a consistent and coherent time series. The composite was updated to incorporate data from 38 Ca ii K archives by Chatzistergos et al. (2020c), producing the most comprehensive time series of plage areas extending from 1892 to present. However, there are still significant data gaps, especially over the first two decades of Ca ii K observations. Furthermore, uncertainties arising from potential instrumental changes and discontinuities within individual archives persist and must be properly addressed to improve our understanding of plage area evolution. For instance, such discrepancies have been reported for plage areas derived using different processing methods from the Kodaikanal data, which show a deterioration in quality over time. Similarly, the Mount Wilson archive includes data of varying quality during solar cycles 15, 19, and 21 (e.g.Ermolli et al. 2009b; Chatzistergos et al. 2019b).

The available Ca ii K plage area time series have been used to investigate periodicities in solar activity. In particular, Ermolli et al. (2018) used a Morlet wavelet on the plage series by Foukal (1996); Tlatov et al. (2009); Chatterjee et al. (2016) using Mount Wilson and Kodaikanal data and reported no other significant periodicity other than the 11-year cycle. Chowdhury et al. (2016, 2022) and Chowdhury et al. (2025) used plage areas from Kodaikanal data derived by Tlatov et al. (2009), Chatterjee et al. (2016), and Singh et al. (2022), respectively, and reported Rieger-type (Rieger et al. 1984) periodicities (130–190 days) as well as quasi-biennial ones (1.2−3.5 years). Quasi-biennial periodicities were also reported by Xu et al. (2024) in the Chatzistergos et al. (2020c) plage area composite series.

**Fig. 22:**
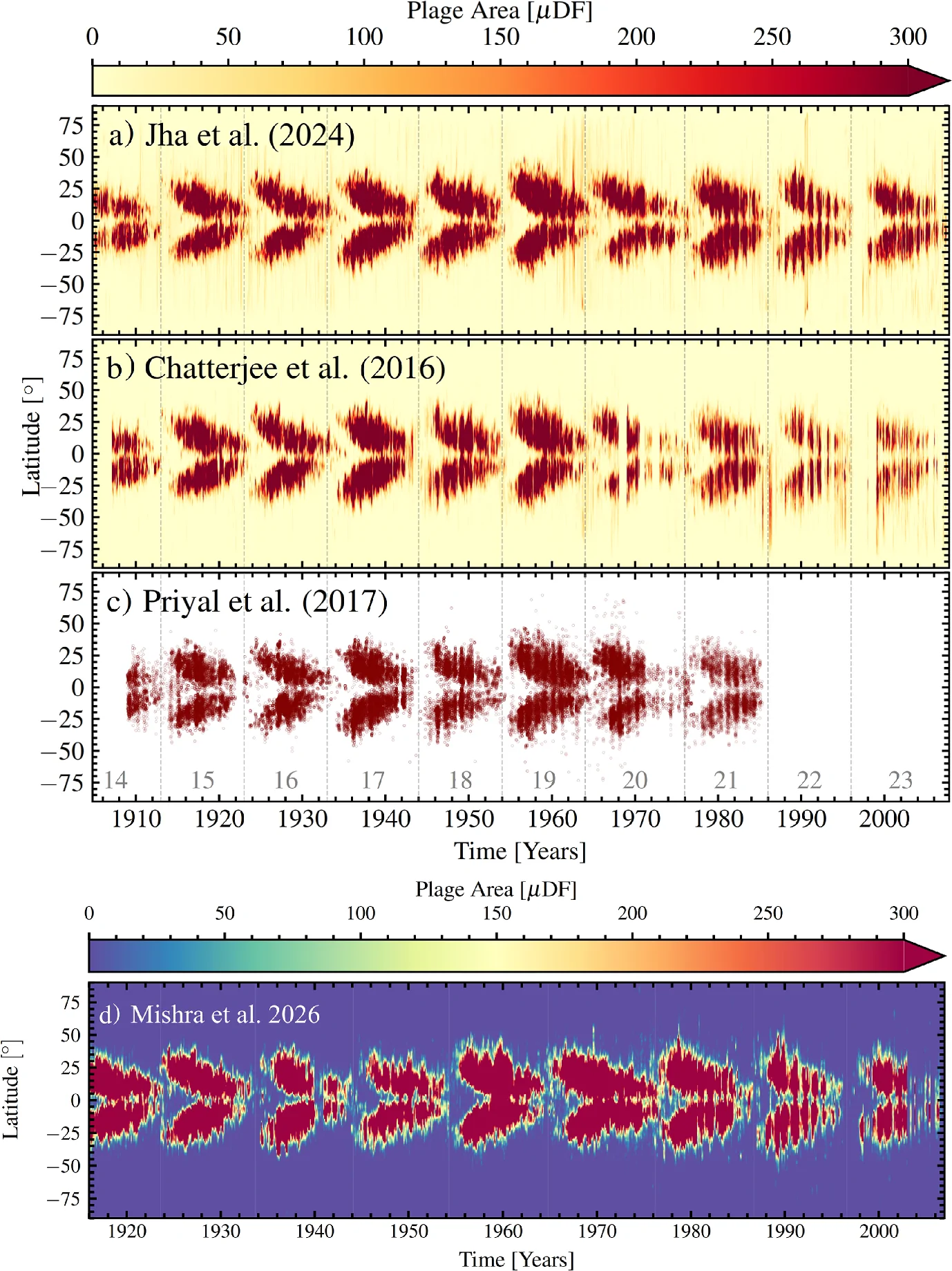
Butterfly diagrams of plage areas derived from Kodaikanal Ca ii K observations. Panels a), b), and d) show plage fractional areas in μDF (in millionths of disc fraction) averaged in latitudinal bins of 1$^\circ$ colour-coded as denoted with the colour bars above the panels. In panel c) plage are marked at the latitude of their centroid. Panels a)–c) were adapted from Jha et al. (2024), while panel d) from Mishra et al. (2026)

**Fig. 23:**
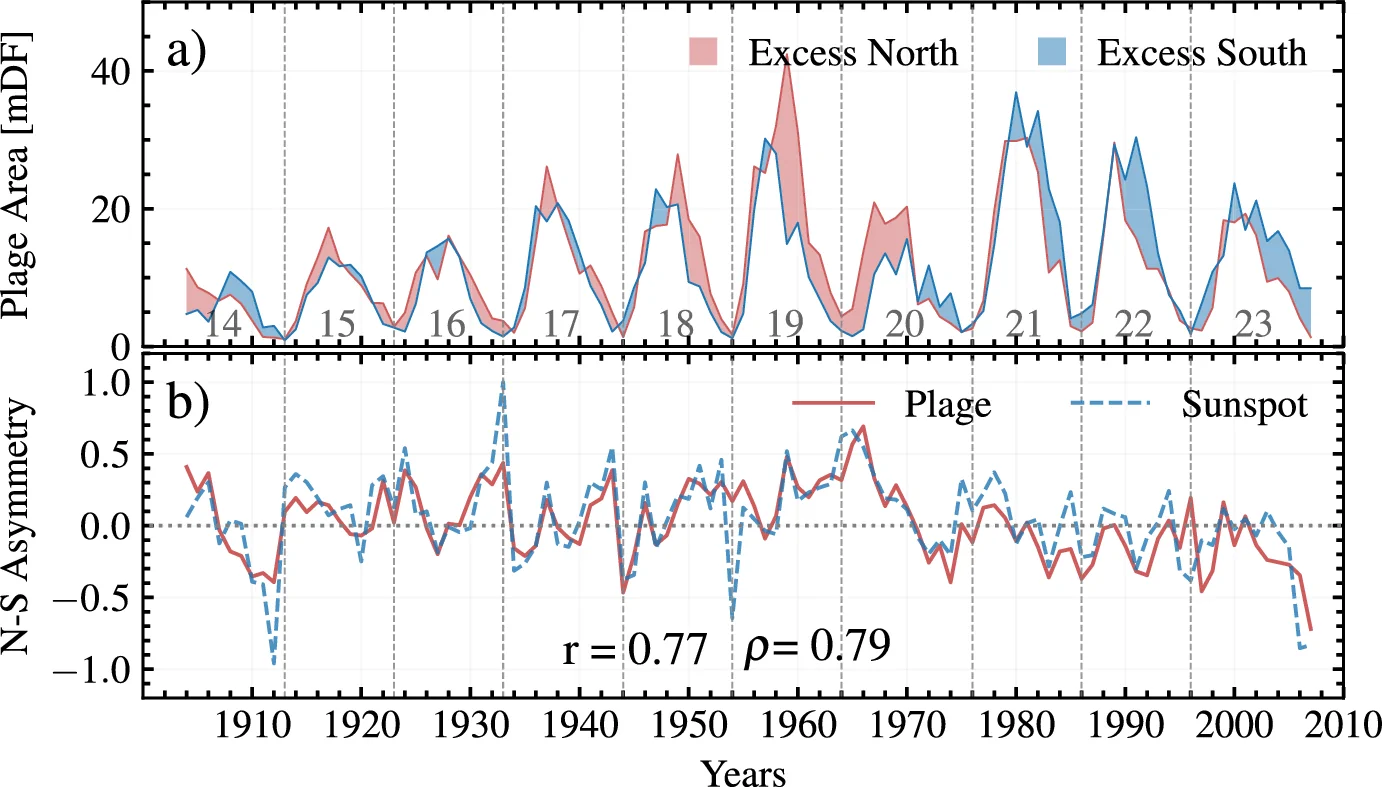
Panel (**a**): Hemispheric annual Ca ii K plage areas derived from Kodaikanal observations by Jha et al. (2024). Red and blue shadings mark the periods when there is excess plage areas in the northern and southern hemisphere, respectively. Panel (**b**): North–South asymmetry in Ca ii K plage areas derived from Kodaikanal observations by Jha et al. (2024) as well as sunspot areas derived from Kodaikanal white-light observations by Jha et al. (2022). Also listed is the Pearson (r) and Spearman (ρ) correlation coefficients. Adapted from Jha et al. (2024)

Besides disc-integrated time-series of plage areas, some studies also derived time-latitude maps, or butterfly diagrams. There have been only five such butterfly diagrams from Ca ii K observations (Coffey and Hanchett 1998; Chatterjee et al. 2016; Priyal et al. 2017; Jha et al. 2024; Mishra et al. 2026). To my knowledge the first such butterfly diagram was produced with McMath data (1942–1979) extended with Mount Wilson (1979–1981) and Big Bear (1981–1987) data hosted at NGDC’s website (Coffey and Hanchett 1998; Harvey 1992). All other four butterfly diagrams were derived from Kodaikanal Ca ii K data (shown in Fig. 22). Jha et al. (2024) presented the most complete butterfly diagram, spanning all available data from 1904 to 2007, and is the only one to use photometrically calibrated data from Chatzistergos et al. (2019c). All of the butterfly diagrams referenced here are based on photographic data, with the exception of Mishra et al. (2026), which employed a machine learning approach to extract plage regions from Kodaikanal drawings (see Sect. 2.5.1). Having the location of plage regions allowed also studies of their North–South asymmetry. To my knowledge, however, this has only been studied by Mandal et al. (2017a), Jha et al. (2024), de Paula et al. (2022), and Dorotovic et al. (2007, 1996) with Kodaikanal (over 1907–1999), Kodaikanal (over 1904–2007), Ebro (over 1910–1937), and Coimbra (over 1996–2006) data, respectively. These studies present a consistent picture of nearly balanced plage areas between the two hemispheres up to solar cycle 18 (Fig. 23), followed by a clear excess of plage area in the northern hemisphere during cycles 19 and 20, and subsequently an excess in the southern hemisphere. However, a comprehensive study combining data from multiple sources is still lacking, largely due to inconsistencies across the various archives (e.g., differences in passband). Consequently, the North–South asymmetry in plage during cycles 21–23 remains uncertain, largely because most studies rely on Kodaikanal data from this period, which are affected by reliability issues.

It is noteworthy that there were studies of Hα plage regions too. In particular, QBSA26 produced for Hα plage regions over 1917–1944 the same qualitative index that was also presented for Ca ii K plage regions. QBSA used Hα observations from Abastumani, Kodaikanal, Ewhurst, Meudon, Mount Wilson, Arcetri, Crimea, Ulugh Beg, and Zürich to get a complete coverage. Diercke et al. (2022) also studied the temporal variation of contrast of plage in Chromospheric Telescope (ChroTel;Bethge et al. 2011) Hα observations over 2012 and 2020. However, I am not aware of any systematic study from historical observations in Hα or other chromospheric observations besides Ca ii K .

**Fig. 24:**
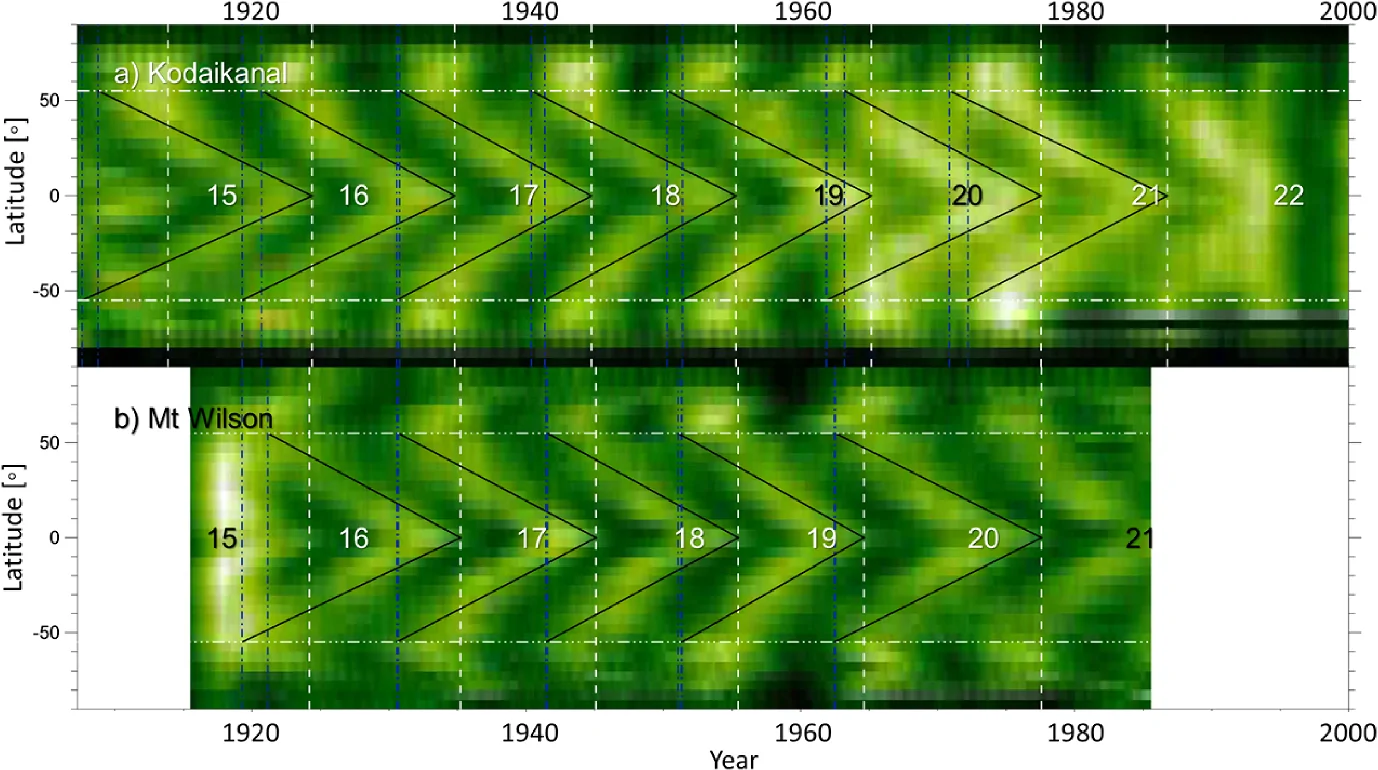
Time-latitude maps of network areas from Kodaikanal (panel **a**) and Mount Wilson (panel **b**) observatories. The areas are expressed as fractional values within 5$^\circ$ latitudinal bins and are colour-coded from black through dark green and bright green to white, indicating increasing area. The black vertical lines, blue dashed lines, and white lines indicate the extended equatorward branches, their onset times, and the latitudes at $\pm 55^\circ$, respectively. The numbers at the centre of each panel denote the conventional solar cycle numbering. Adapted from Chatterjee et al. (2019). ©AAS, reproduced with permission

### Network regions

Chromospheric network is typically studied in Ca ii K observations. Network regions are small-scale magnetic flux concentrations that manifest as bright, web-like structures outlining darker cellular patterns. These structures mark the boundaries of supergranular cells and are often described as the chromospheric counterparts of the supergranulation pattern observed in the photosphere. Understanding the evolution and variability of network regions is crucial to better understand their contribution to long-term variation of solar irradiance (e.g.,Solanki et al. 2002).

To facilitate their study, network regions are sometimes categorized into three types: active, enhanced, and quiet network. This classification aims to distinguish between decaying remnants of active regions (active network) and the persistent “quiet Sun” network which is unrelated to active regions. The latter are typically subdivided into enhanced and quiet network based on their brightness, with the enhanced network comprising the brighter features and the quiet network the fainter ones. However, in practice, this distinction is often ambiguous and is typically made based on intensity contrast alone rather than strict physical criteria. Here, we use the term network inclusively to refer to all three categories unless otherwise specified.

#### Network areas

Segmentation approaches (see Sect. 2.6.4) are applied to identify the network regions. However, the choice of segmentation parameters varies substantially between different studies, more so than for Ca ii K plage regions. A result of this is that reported estimates of the fractional area covered by network regions vary significantly between studies. Values range from as low as 0.02, as derived from Mount Wilson data (Foukal 1998), to as high as 0.4 based on Rome/PSPT observations (Ermolli et al. 2003a). Despite this discrepancy, a consistent result across multiple studies is that the network area generally varies in phase with the solar cycle (Caccin et al. 1997, 1998a; Foukal 1998; Worden et al. 1998a, b; Ermolli et al. 2003a; Priyal et al. 2014b; Chatzistergos 2017; Priyal et al. 2017; Ermolli et al. 2022a; Singh et al. 2023).

The discrepancies between studies are primarily due to inconsistent definitions of what constitutes a network region, variations in data quality, and differences in image processing techniques (see Sect. 2.6). Unlike Ca ii K plage, which exhibit high contrast and are relatively easy to isolate, network features are more subtle and often have contrasts comparable to artefacts in historical data. This makes their detection highly sensitive to the image processing performance and the choice of segmentation method. For example, using high-quality Rome/PSPT observations, Chatzistergos (2017) showed that network area estimates are strongly influenced by thresholding choices. When a fixed intensity threshold was used for segmentation, network areas varied in phase with the solar cycle. In contrast, when a dynamic threshold based on the standard deviation of disc contrast was applied, the results showed anti-phase behaviour. However, this approach biases the contrast statistics by including active regions in the calculation of the standard deviation and, consequently, the segmentation threshold. When active regions were excluded from the contrast calculation, the resulting network area showed little to no variation with the solar cycle. These findings support earlier conclusions by Harvey (1993, 1994), who found that solar cycle variability is much stronger in large-scale magnetic features like active regions compared to smaller ephemeral features like those forming the network.

A latitudinal dependence of network area variation with solar cycle has also been suggested (e.g.Devi et al. 2021; Chatterjee et al. 2019; Sowmya et al. 2023). Devi et al. (2021), using Kodaikanal data, showed that the amplitude of area changes decreases from the equator up to about ±50$^\circ$, with a subsequent increase toward the poles. This latitudinal behaviour could offer insights into the mechanisms governing magnetic flux transport and surface diffusion. Chatterjee et al. (2019) analysed the latitudinal evolution of network regions (Fig. 24) using Carrington maps from Kodaikanal and Mount Wilson observatories (Chatterjee et al. 2016; Sheeley et al. 2011). They quantified network areas within 5$^\circ$ latitude bands across the solar disc and applied temporal smoothing over 200 Carrington rotations (approximately 15 years). Their results revealed distinct equatorward-migrating branches of network activity that originate near ±55$^\circ$ latitude and migrate to the equator over ∼15 years. These “butterfly wing” patterns resemble those seen in sunspot distributions but with distinct temporal and spatial signatures, reflecting the evolution of smaller-scale magnetic features.

The presence of a secular trend in network areas, i.e. a long-term increase or decrease across multiple solar cycles, remains a topic of debate. However, existing studies hint at a small to negligible secular trend in network areas between activity minima. For example, Foukal and Milano (2001), based on Mount Wilson (used over 1915–1984) and Sacramento Peak (used over 1985–1999) data during cycle minima between 1914 and 1996, argued against any long-term trend. Nevertheless, their analysis was based on merely three images per minimum, and the derived network area fractions displayed substantial scatter (between ∼0.10 and 0.19). Singh et al. (2023) derived network areas over 1905–2015 from Kodaikanal, Mount Wilson, and MLSO data. They found network areas during activity minima to vary between approximately 4–7%. It is important to note that for such studies, uncertainty arises from the combination of different datasets as systematic differences between them may affect the homogeneity of the resulting series (see Sect. 2.3.1). Additional uncertainty stems from possible temporal inconsistencies within each archive (for instance discrepancy in Kodaikanal data since about 1980 in Singh et al. 2023). Finally, their use of photometrically uncalibrated data introduces a significant uncertainty.

To my knowledge, only two studies have investigated polar network regions using Ca ii K  data (Priyal et al. 2014a; Mishra et al. 2025), both based on Kodaikanal observations. The most recent one, Mishra et al. (2025), derived polar network area series spanning 1904–2022 from photometrically calibrated Kodaikanal (Chatzistergos et al. 2018b) and Rome/PSPT Ca ii K  observations. Two series were produced using the latitudinal bands of $\pm (55^\circ - 90^\circ )$ and $\pm (70^\circ - 90^\circ )$. Both exhibited a clear anti-phase relationship with the solar cycle, similar to that of photospheric polar faculae. They also showed a strong linear correlation between their values at solar minimum and the amplitude of the subsequent cycle in sunspot number.

Overall, network areas remain less well studied than those in Ca ii K , largely due to challenges in their analysis. A comprehensive study of network regions across different Ca ii K archives is still missing.

#### Network cell characteristics

A key structural feature of the chromospheric network is its spatial alignment with supergranular boundaries in the photosphere. This connection was first noted by Simon and Leighton (1964), sparking numerous studies on network cell morphology and evolution using Ca ii K observations (e.g., Sotnikova 1978; Singh and Bappu 1981; Raghavan 1983; Münzer et al. 1989; Kariyappa and Sivaraman 1994; Hagenaar et al. 1997; Berrilli et al. 1998, 1999; Ermolli et al. 1998; Raju and Singh 2014; Raju 2020; Sowmya et al. 2022; Rajani et al. 2022, 2023).

Most of these studies report smaller average cell sizes in Ca ii K images than the ∼32 Mm diameter of supergranular cells found in the photosphere by Simon and Leighton (1964). For instance, average network cell sizes were estimated as 13–18 Mm with South Pole Ca ii K data (Hagenaar et al. 1997), 22–32 Mm with MLSO (McIntosh et al. 2011), and ∼22 Mm with Kodaikanal (Singh and Bappu 1981) Ca ii K data. The latter value, however, increased to ∼32 Mm when an autocorrelation method was used instead of manual selection, aligning more closely with the original findings by Simon and Leighton (1964).

Differences in methodology and data sources contribute to the spread in reported values (Rincon and Rieutord 2018). Importantly, the relationship between network cell size and solar activity remains under debate. While several studies (Singh and Bappu 1981; Raghavan 1983; Kariyappa and Sivaraman 1994; Rajani et al. 2022) found that cell sizes decrease with increasing solar activity, others present conflicting results. Notably, Chatterjee et al. (2017b) reported an overall increase in cell size with activity when analysing the full solar disc, but an anti-correlation when considering only quiet Sun regions. Furthermore, Caccin et al. (1998a) observed an activity-dependent asymmetry in the contrast distribution of network regions in Arcetri spectroheliograms covering 1950–1970.

### Filament regions

**Fig. 25:**
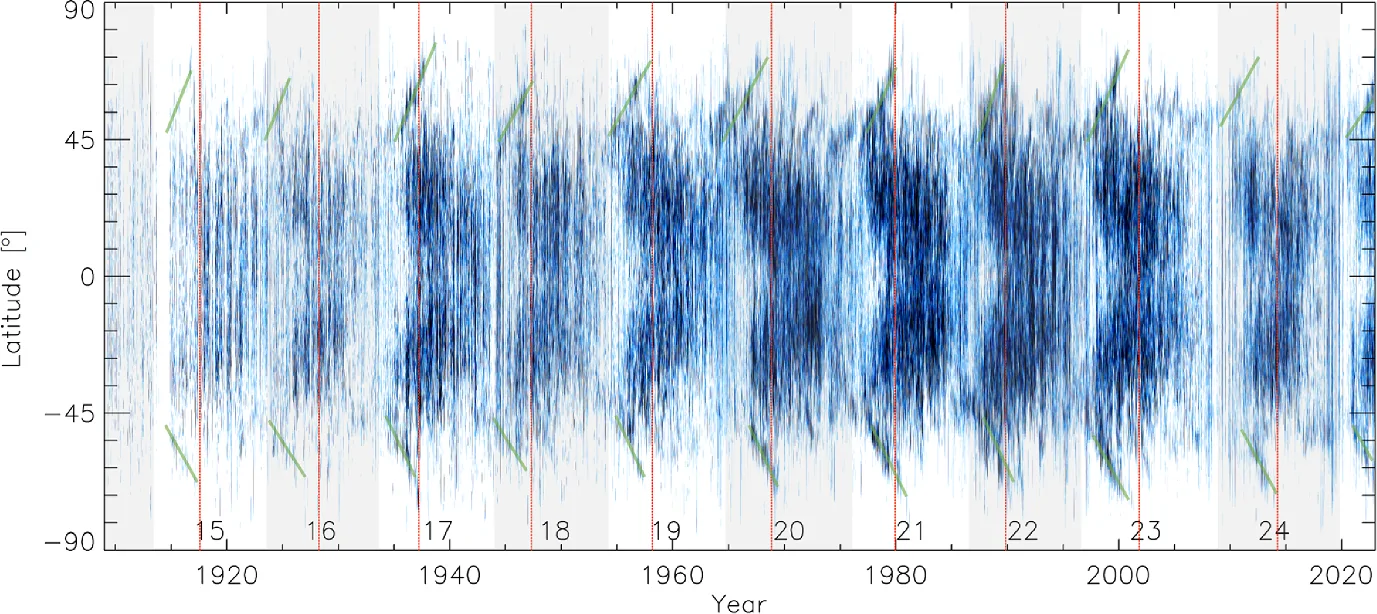
Composite Hα filament butterfly diagram by Chatzistergos et al. (2023a). Shown are daily mean values within latitudinal strips of 1$^\circ$ as fractions of the area of the entire solar disc. The areas are presented colour-coded, with white for no observed filaments, while blue denotes the filament fractional areas getting darker with growing areas up to the saturation level of 3$\times 10^{-4}$. The period of individual cycles is marked by shading grey the even-numbered cycles. The numbers at the lower part of the panel denote the conventional solar cycle numbering, while vertical red lines mark the date of the maximum of each cycle. Green lines mark the approximate poleward migration of filaments. Adapted from Chatzistergos et al. (2023a)

Filaments are dark, elongated features on the solar disc seen in observations sampling the chromosphere. Filaments are most clearly observed in Hα observations (see Fig. 10), which is why such data have been widely used for the systematic monitoring of filaments. Filaments also appear in He i 10830 Å observations and in several other chromospheric lines; however, with the exception of Ca ii K , the available records are comparatively short, as discussed in Sect. 2. In particular, Ca ii K observations show filaments mostly with relatively narrow passbands. For example in Fig. 6 there is a faint filament right above the decaying region in the northern hemisphere when seen with the narrow passband of 0.15 Å of Meudon, while the filament becomes fainter in the Rome/PSPT observation with 2.5 Å passband, and it is not visible any more in the San Fernando observation with 9 Å passband. Filaments also become fainter when the observation is not centred at the core of the Ca ii K or Hα lines (See bottom row in Fig. 6 for examples of offband Ca ii K observations).

Various observatories that performed Hα observations recorded filament characteristics and published them in their reports. For instance, RGO kept records of Hα filaments over 1929–1949 (Jones 1957) with Hale’s SHS (Hale 1929). QBSA26 included information about Hα  filaments over 1917–1944 from a network of observatories (Abastumani, Kodaikanal, Ewhurst, Meudon, Mount Wilson, Arcetri, Crimea, Ulugh Beg, and Zürich) in the same qualitative way as we discussed earlier for Ca ii K plage. That is that they reported qualitative estimates of brightness and extent of filaments across the solar disc by manually classifying them on a scale from 0 (no filament) to 5 (maximum darkness and size). The Meudon synoptic charts (Sect. 2.5.2) were also accompanied with information about the filament locations. Wright (1991) used Hα observations from Boulder (1964–1973) and Culgoora (1974–1980) and created a database of filament disappearances.^27^

Over the last three decades various methods have been developed to extract masks and characteristics of filaments from digital Hα images (e.g.Denker et al. 1999; Shih and Kowalski 2003; Fuller et al. 2005; Benkhalil et al. 2005; Yuan et al. 2011; Tlatov et al. 2016a; Chatterjee et al. 2017a; Lin et al. 2020; Suo 2020; Chatzistergos et al. 2023a; Diercke et al. 2024). These have led to the extraction of filament characteristics and the production of filament butterfly diagrams as well as Carrington maps. In particular, Meudon Hα data were analysed by Zharkova et al. (2005, covering 1996–2004) Big Bear data by Hao et al. (2015, covering 1988–2013), MLSO data by Hao et al. (2013, covering 1998–2009), and ChroTel data by Diercke and Denker (2019, covering 2012–2018). Diercke et al. (2024) combined data from ChroTel, Kanzelhohe, and GONG to derive a filament butterfly diagram covering 2010–2021. Diercke et al. (2022) also studied the temporal variation of contrast of filaments in Hα observations from ChroTel between 2012 and 2020 showing that it varies in phase with the solar cycle, but Hα deficit due to filaments peaks shortly after the cycle maximum.

Historical Hα  archives have also been used to extract filament characteristics. Tlatov et al. (2016a) extracted filament masks from the Meudon synoptic charts over 1919–1985 and combined these with masks derived from Kislovodsk Hα  observations spanning 1986–2014. Similarly, Mazumder et al. (2021) extracted filament masks from the Meudon (see Sect. 2.5.2) and McIntosh (see Sect. 2.5.6) synoptic charts. More recently, Routh et al. (2025) extracted information about polar filaments from Meudon synoptic charts. While Meudon data already provided information on filament locations, the works by Tlatov et al. (2016a); Mazumder et al. (2021); Routh et al. (2025) enabled a more quantitative analysis of their morphology. Chatterjee et al. (2017a) analysed Kodaikanal Hα  data, constructing Carrington maps and a filament butterfly diagram covering 1914–2007. Priyadarshi et al. (2023) extracted filaments over 1954–1976 from the Kodaikanal drawings (Sect. 2.5.1) with a K-means clustering algorithm. Lin et al. (2020) extended this effort by analysing Kodaikanal, Sacramento Peak, Kanzelhöhe, Big Bear, and Huairu data to produce Carrington maps and extract filament information over 1912–2018. The most comprehensive analysis to date was performed by Chatzistergos et al. (2023a), who incorporated data from 15 archives covering 1909–2022. This includes the major historical archives available in digital format at the time (Arcetri, Boulder, Coimbra, Kanzelhöhe, Kharkiv, Kodaikanal, McMath-Hulbert, Meudon, Mitaka, and Sacramento Peak) as well as modern digital archives (Big Bear, Catania, Larissa, Pic du Midi, and Upice). Notably, this is the only filament analysis performed on photometrically calibrated historical Hα  data. They constructed Carrington maps for each archive and extracted filament areas and locations, combining all data to produce a composite filament butterfly diagram (Fig. 25).

Overall, filament areas vary in phase with the solar cycle. In particular, the filament areas reported by Chatzistergos et al. (2023a) are roughly half the sunspot areas reported by Mandal et al. (2020). However, filament areas do not follow the same solar cycle ranking as sunspots. The filament butterfly diagram (Fig. 25) exhibits features similar to those seen in sunspot and plage records, while also clearly highlighting the poleward “rush” of filaments. Despite the many studies of Hα  filaments, most historical archives remain unexplored (see Sect. 2.3.2), and significant gaps in the early filament records could potentially be filled if these data are digitized and analysed.

### Prominence regions

**Fig. 26:**
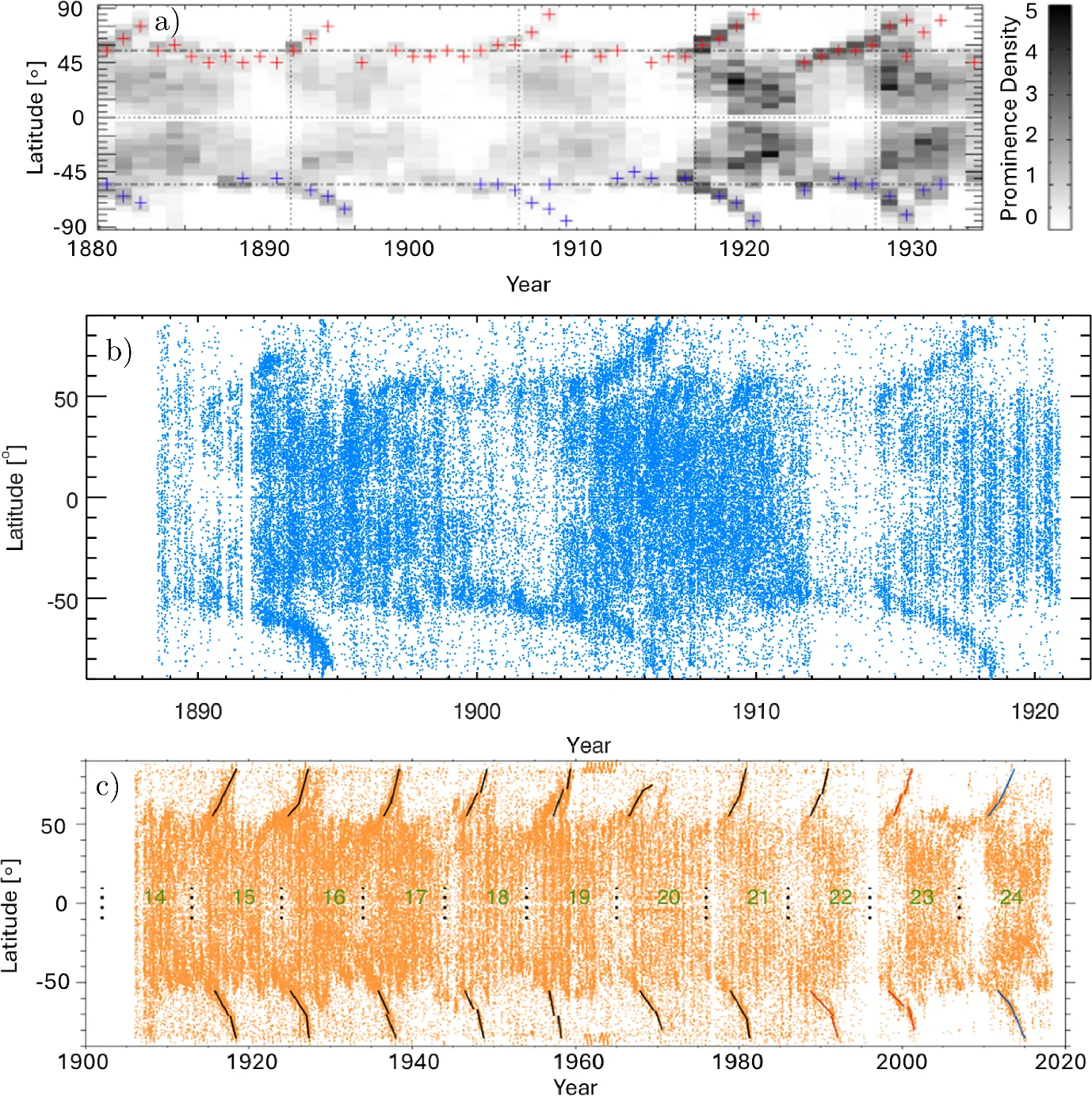
Butterfly diagrams of prominences. Produced by Bocchino (1933b, panel a), from the Zürich records (Illarionov and Arlt 2022, panel b), and by (Chatterjee et al. 2020, panel c) combining Kodaikanal Ca ii K disc-blocked prominence records with Meudon and Kanzelhöhe Hα ones. Panel a is adapted from McIntosh et al. (2021), while panel c from Chatterjee et al. (2020)

Prominences represent the same physical structures as filaments, with the latter seen in projection against the disc and the latter observed beyond the solar limb (see review byParenti 2014). Prominences were first observed during solar eclipses. One of the earliest accounts that likely refers to prominences appears in the Chronicle of Novgorod (Hetherington 1996) in connection with the eclipse of 1 May 1185, as observed from Kiev. Several other historical accounts also appear to allude to prominences, including descriptions of the eclipse of 3 June 1239 (Hetherington 1996) and the eclipse of 2 May 1733 reported by Vassenio (1733). These early records, however, provide only vague qualitative descriptions. For example, the Chronicle of Novgorod (Hetherington 1996) describes that “from its [the Sun’s] horns came out somewhat like live embers”, a phrase interpreted as a reference to prominences.

Some of the earliest photographs of solar prominences were taken by Secchi (1875) and de La Rue (1861) during the eclipse of 18 July 1860. A comparison of Secchi’s photograph with those taken by de La Rue from a different observing site during the same eclipse demonstrated that prominences are genuine solar features rather than atmospheric or instrumental effects. Following this discovery and the development of the prominence SHS (see Sect. 2.1.1), systematic observations of prominences began. Thus, prominences became the first non-photospheric solar feature to be observed in a systematic manner. The earliest such observations began in the 1870 s using a prominence SHS in the Hα  or He i D$_3$ (5877 Å) lines (see Sect. 2.1.1) and were soon complemented by SHG and filter observations in the Hα  and Ca ii K  lines (See Sect. 2.3.1 and 2.3.2). Figure 8 shows examples of prominence observations, with most of the early ones surviving as drawings like those shown in panels a and b.

These historical observations have been used to derive timeseries of the number of prominences, sometimes also maintaining information about their location. Such records survive from at least the observatories in Coimbra (Carrasco and Vaquero 2022, over 1929–1944), Worthing (Newbegin 1903, 1947, atleast over 1901–1946), Zürich (Illarionov and Arlt 2022, over 1888–1920; shown in Fig. 26b, Madrid (Aparicio et al. 2023, over 1906–1957), Catania (Riccò 1909), Rome (Secchi 1875; Carrasco et al. 2021b) as well as by Evershed in Kenley (Evershed 1892, over 1880–1904) and then from Kodaikanal (until 1914).

Bocchino (1933a, 1933b) collected Hα observations from Respighi, Secchi, and Tacchini between 1980 and 1911 that were published in the “Memorie della Società degli Spettroscopisti Italiani” (e.g.Lorenzoni 1872), which he complemented with observations from Catania, Zürich, Madrid, and Arcetri until 1931 to derive a timeseries of prominence areas in 5$^\circ$ latitudinal bins. The butterfly diagram produced by Bocchino (1933a, 1933b) is shown in Fig. 26a. Notice the change in the overall level of prominence density after 1911 marking the separation between the data from the “Memorie della Società degli Spettroscopisti Italiani” and the rest. Thus, caution is warranted for using these data, although the information on location, and thus information about the polar rush of prominences is more reliable. Mateos et al. (2025) recently presented a software for automated detection of solar prominences, which they evaluated on drawings made by Eric H. Strach over 1969–2008 (Carrasco et al. 2019). Tlatova and Nagnibeda (2017) digitised the sketches of direct vision SHS prominence observations published in the “Memorie della Società degli Spettroscopisti Italiani” over 1922–1935 and extracted information (location, area, height) of the prominences. The most comprehensive study of prominences to date was performed by Chatterjee et al. (2020) who analysed Kodaikanal disc-blocked Ca ii K observations. Because of the deterioration in the quality of Kodaikanal data, the data after 1980 were not used by Chatterjee et al. (2020). Instead, prominence information for later periods was obtained from Meudon (1980–2002) and Kanzelhöhe (2000–2018) Hα  observations. Combining all three datasets, Chatterjee et al. (2020) constructed a butterfly diagram of prominences covering 1906–2018. The butterfly diagram by Chatterjee et al. (2020) is shown in Fig. 26c. The prominence butterfly diagram resembles that of filaments (Fig. 25), but the poleward rush of prominences is even more pronounced.

### Flares

**Fig. 27:**
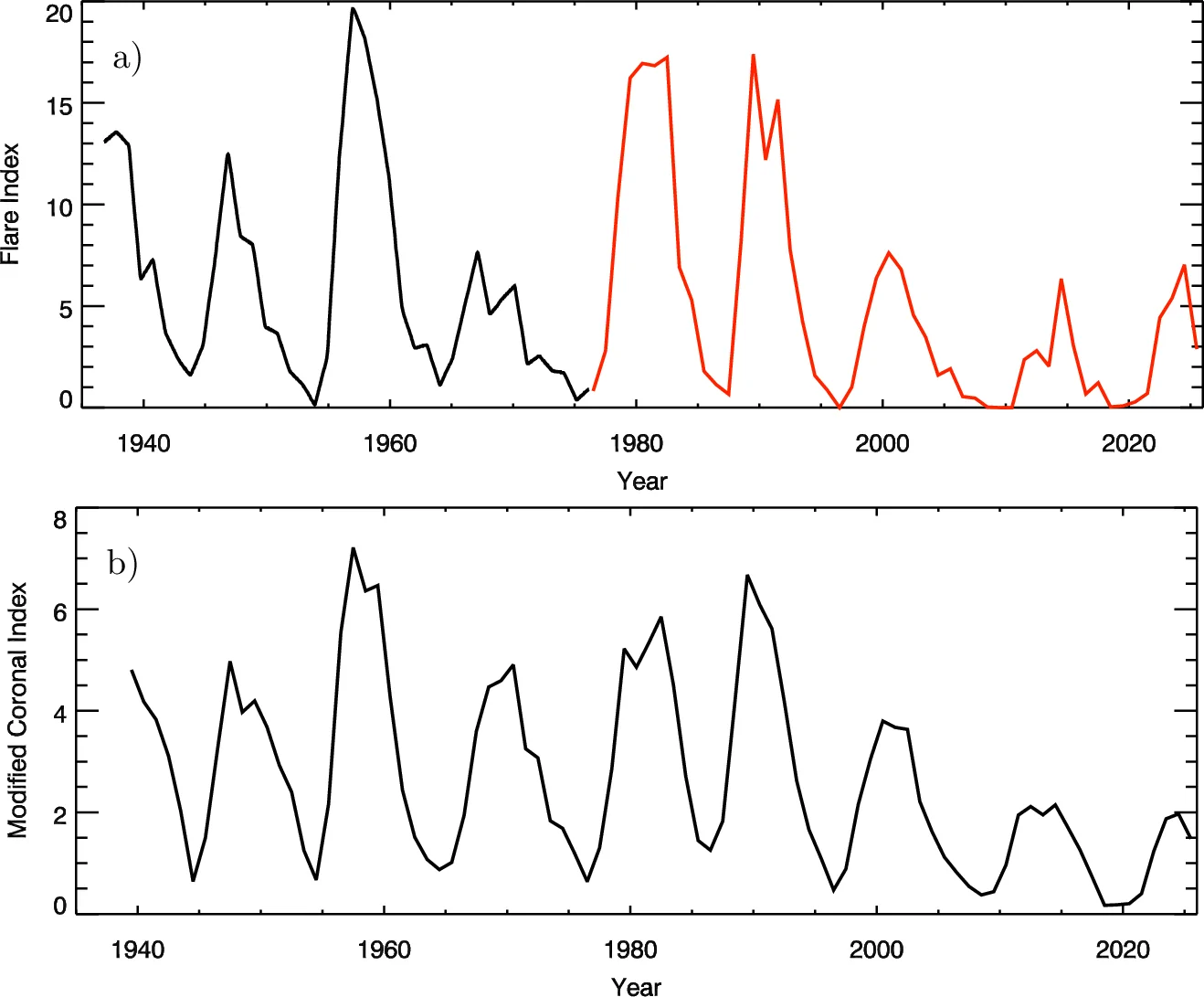
Panel **a**: Flare index *q* (Eq. 5). The index comprises of two parts produced by Knoska (1985, black) and Atac (1987, red) Panel **b**: Modified coronal index

Flares are highly energetic eruptions in the solar atmosphere driven by magnetic reconnection in active regions. They emit radiation across a broad wavelength range, from radio waves to gamma rays (e.g. Woods et al. 2004; Benz 2016). The first reported solar flare was observed on 1 September 1859 by Carrington (1859) during white-light observations conducted for sunspot drawings. Only a few additional white-light flare reports exist from the second half of the 19th century. Notable examples include the events of 10 September 1866 (Valderrama 1886; Vaquero et al. 2017), 3 February 1872 (Secchi 1872; Hayakawa et al. 2023), 17 June 1891 (Trouvelot 1891), and 15 July 1892 (Rudaux 1892). During the same period, a few flares were also reported as “brilliant reversals” in Hα observations with a SHS over sunspots (Hale 1931; Newton 1943; Neidig and Cliver 1983).

But as we mentioned in Sect. 2.1.1 the SHS over this period did not allow for on-disc observations, something that became possible with the development of the SHG. The flare of 15 July 1892 was the first to be photographed with a SHG in the Ca ii K  line (Hale 1892a, 1931). Subsequently, Hale (1931) reported Hα  SHG photographs of flares observed at several observatories, including Yerkes (10 September 1908), Mount Wilson (12 May 1909; 10 November 1916; 7, 8, 14, and 16 February 1917; 8 and 9 August 1917), Kodaikanal (22 February 1926), and Meudon (13 October 1926). However, the long exposure times required for SHG photography made it not well suitable for flare observations.

This limitation was mitigated by improvements to the SHS, which enabled rapid full-disc monitoring in monochromatic light, including the Hα  line, crucial for flare detection, thereby significantly enhancing flare patrol capabilities. Subsequently, numerous ground-based Hα  flare patrol programs were established since the 1930 s. In particular, IGY coordinated high-cadence solar observations in Hα in numerous sites around the globe with one of the aims to record information about flares (Dodson and Hedeman 1962). Early observations were primarily carried out visually with a SHS, whereas later records were obtained using filter-based observations, either through visual inspection or by recording on photographic film and, eventually, in digital format.

Beyond the IGY flare records various observatories produced flare accounts, for instance RGO (1929–1949 Jones 1954, 1957), Kanzelhöhe^28^ (since 1948), Kandili28 (since 1976), Ondřejov (1939–1970;Švestka 1951; Kotrc 2009), Anacapri (around 1955 Öhman 1968), USAF/SOON (1938–2015)28. QBSA26 collected data from most of the Hα archives active at the time and produced a catalogue of flares between 1934 and 1989. They recorded the start and end time of the flaring, along with coordinates and the importance parameter. The importance parameter is taking into account the intensity of the flare (in scale of 1 to 3 being faint to very bright, labelled as F, N, and B, respectively) and its area (in five groups: 0–2, 2–5, 5-−12.5, 12.5-−24.8, >24.8 square degrees).

Different indices have been presented aiming for a more quantitative measure of daily flare activity. Kleczek (1952) proposed the Hα flare index, defined as

5 $$\begin{aligned} q = i \times t, \end{aligned}$$

where *i* corresponds to the intensity scale of importance and *t* is the flare duration in minutes (Atac 1987; Ataç and Özgüç 1998). Kleczek (1952); Knoska and Petrasek (1984) produced this index over 1936 to 1976 at Ondřejov Observatory, while Kandili observatory^29^ continued this index after 1976 (Ozguc et al. 2021). This flare index is shown in Fig. 27.

Three additional, flare indices have been proposed in the literature. The first was suggested by Dodson and Hedeman (1962) and is defined as the weighted sum of all flares within a year according to their importance class. Later, Dodson and Hedeman (1971) introduced the Comprehensive Flare Index (CFI)28, which was produced for the period 1955–1980. The CFI is calculated as the sum of five distinct measures of flare importance, including the Hα  importance class and associated radio flux information. The last index was developed at the High Altitude Observatory and incorporates the flare brightness relative to the surrounding quiet Sun, the projected flare area (expressed in millionths of the solar hemisphere), and the flare duration in minutes (Sawyer 1967).

Although Hα observations provide the longest record of flares, measurements of X-ray emission allow to produce a more objective and quantitative index of flare activity (e.g. Aschwanden and Freeland 2012). X-ray measurements need to be made outside of Earth’s atmosphere, and the first ones were achieved with rockets performed over the period 1948–1959 (Thomas 1970). More systematic observations, however, were performed with satellites since 1961 (e.g. Kreplin et al. 1962; Thomas 1970). Such measurements have been performed with the Naval Research Laboratory’s (NRL) SOLar RADiation (SOLRAD) satellites (Neupert 2011) and since 1975 with the Geostationary Operational Environmental Satellites (GOES; Grubb 1975; Lemen et al. 2004). A flare index describing the magnitude of a solar flare has been defined based on the 1-minute averaged soft X-ray irradiance covering $\Delta \lambda =$1–8 Å at the time of peak emission (**see e.g.** Woods et al. 2024). Flare indices are designated by a letter-number notation corresponding to the base-10 logarithm of the peak irradiance, with classes X ($\ge 10^{-4}$ W m$^{-2}$), M ($10^{-5}$ W m$^{-2}$), C ($10^{-6}$ W m$^{-2}$), B ($10^{-7}$ W m$^{-2}$), and A ($10^{-8}$ W m$^{-2}$). The numerical component specifies the magnitude within each class. For example, a flare with a peak irradiance of $3 \times 10^{-4}$ W m$^{-2}$ is categorised as X3, while one with a peak irradiance of $4.29 \times 10^{-6}$ W m$^{-2}$ as a C4.2 flare. Note that the flare index is determined using truncated, rather than rounded, peak irradiance values. The most intense GOES soft X-ray flare recorded to date occurred on 4 November 2003 and was X43 (Hudson et al. 2024). However, Cliver et al. (2022) note that A- and B-class flares are under-reported in the GOES catalogue because, during periods of heightened solar activity, they are difficult to distinguish from the quiescent background. Based on the GOES catalogue, numerous studies have shown that the frequency distribution of flare peak flux, especially for moderate to large events, is well approximated by a power-law (e.g. Veronig et al. 2002; Aschwanden et al. 2016; Plutino et al. 2023).

The most powerful solar flares observed since 1975 have reached bolometric energies of a few 10$^{32}$ erg (e.g. Emslie et al. 2012). A key open question is the maximum bolometric energy that a solar flare can attain. One approach is to extrapolate the observed flare frequency distribution to higher energies; however, such statistical inferences are highly uncertain because they rely on a comparatively short and sparsely sampled record of the most extreme events (see e.g. Vasilyev et al. 2026a). Aulanier et al. (2013) used 3D Magnetohydrodynamic (MHD) simulation for eruptive flares scaled to the largest sunspot group on record (April 1947) and found an upper limit for solar flare energies of approximately $6\times 10^{33}$ erg.

Krivova et al. (2026) offered an empirical estimate of the Sun’s maximum bolometric flare energy using statistical relationships between active region areas, flare-ribbon areas, and released energy (Kazachenko et al. 2017). Extrapolation of these relations to the Great Sunspot of 8 April 1947 suggests a maximum flare energy of a few $10^{34}$ erg. Although, they caution that active region clustering might make larger values plausible. Taken together, these statistical and physical considerations indicate that, although the Sun could in principle produce flares more energetic than those observed in the modern era, plausible upper limits likely confine even the most extreme events to energies of a few $10^{34}$ erg.

### Chromospheric emission

There are two main chromospheric emission indices, the Mg ii and Ca ii K index. These emission indices provide diagnostics of solar chromospheric variability, ultraviolet (UV) variability, and magnetic activity.

#### Ca ii K emission index

Since the late 1960 s, measurements of Ca ii K  emission have been obtained using high-resolution spectrographs. These observations sample multiple wavelength points across the Ca ii K  line, covering the full line profile and enabling the derivation of several parameters that characterise its shape. Such measurements have been carried out both as full-disc-integrated observations and over different latitudinal bands.

The principal quantity derived from these data is the 1 Å Ca ii K  emission index, defined as the equivalent width of a 1 Å band centred on the line core. In addition, several diagnostics describing the line profile and its asymmetry are routinely determined. These include the relative strength of the blue K2 peak with respect to the K3 intensity, the wavelength separation between the blue and red K2 emission peaks, the separation of the blue and red K1 minima, and the ratio of the blue to red K2 peak intensities (see Fig. 6 for the location of the K1, K2, and K3 features).

To my knowledge, only a limited number of sites have performed such long-term observations. The most prominent archives originate from NSO Sacramento Peak (1967–2015; Evans 1967; Pevtsov et al. 2014), Kitt Peak SOLIS Integrated Sunlight Spectrometer (ISS, 2006–2017; Keller et al. 2003), and the Kodaikanal Observatory (since 1969; Bappu 1967; Sindhuja and Singh 2015; Srinivasa et al. 2025). Additional, shorter time series are available from Utrecht (1979–1982; Oranje 1982, 1983). Data from the Meudon Observatory since 2017 (Malherbe and Dalmasse 2019) can also be used to derive the Ca ii K  emission index.

#### Mg ii index

The Mg ii index is defined as the ratio of the emission in the h and k line cores (2803 and 2796 Å) to the nearby continuum (Heath and Schlesinger 1986; Snow et al. 2019). As a ratio of nearby fluxes, it is less affected by instrumental effects than the fluxes themselves (see e.g. Snow et al. 2014). This index provides a proxy for spectral solar irradiance (SSI) variability from the UV to the EUV (e.g. Dudok De Wit et al. 2009).

Regular space-based measurements of the solar Mg ii h and k resonance lines have been conducted since the late 1970 s. To ensure long-term consistency, the Institute of Environmental Physics (IUP, for Instituts für Umweltphysik in German) at the University of Bremen has combined various existing measurements (see e.g. Snow et al. 2014) into a composite Mg ii index series spanning the period from 1978 to the present (Viereck et al. 2001).^30^

### Coronal emission

Emission in several coronal lines has been measured during solar eclipses and, since the invention of the coronagraph, through regular ground-based observations (see Sect. 2.4.1; Lyot 1930, 1939). Among these, the Fe XIV 5303 Å “green coronal line”, the Fe X 6374 Å “red line”, and the Ca XV 5694 Å “yellow line” have the longest observational records, extending back to around 1930 for coronagraph data. In particular, the intensity of the Fe XIV 5303 Å line depends on plasma density and temperature and is modulated by local magnetic fields, making it a valuable diagnostic of coronal conditions (Rybanský et al. 2005).

Time series of integrated coronal line intensities have been obtained at several individual observatories (e.g., Norikura and Kislovodsk; Sakurai et al. 1999; Guseva 2019). These measurements are typically performed around the solar limb with an angular resolution of at least 5$^\circ$, proceeding counter-clockwise from the north to the south pole at a height of about 50” above the limb, and expressed in millionths of the disc-centre intensity at the same wavelength (Rybanský et al. 2005). Such observations provide information on the long-term variability of coronal emission at different characteristic temperatures. However, the visibility of some lines, for example the Ca XV 5694 Å yellow line, is largely restricted to periods of strong solar activity, preventing the formation of continuous long-term records.

To obtain a temporally extended measure of coronal variability, the coronal index (CI) was introduced based on the daily intensity of the Fe XIV 5303 Å line (Rybansky 1975; Minarovjech et al. 2011). The QBSA26 dataset provides tabulated limb intensities of the Fe XIV 5303 Å line at 5$^\circ$ intervals, combining observations from multiple sites worldwide over the period 1947–2009. In parallel, Rybansky (1975); Minarovjech et al. (2011) compiled a homogeneous dataset of green coronal emission for 1939–2009 by intercalibrating measurements from different observatories onto the Lomnický štít photometric scale. The CI was subsequently extended to the present by incorporating 284 Å observations from the SoHO/EIT (Delaboudinière et al. 1995), and since 2010 SDO/AIA 211 Å observations, resulting in a modified coronal index (Dorotovic et al. 2014). Figure 27b) shows this modified index.

**Fig. 28:**
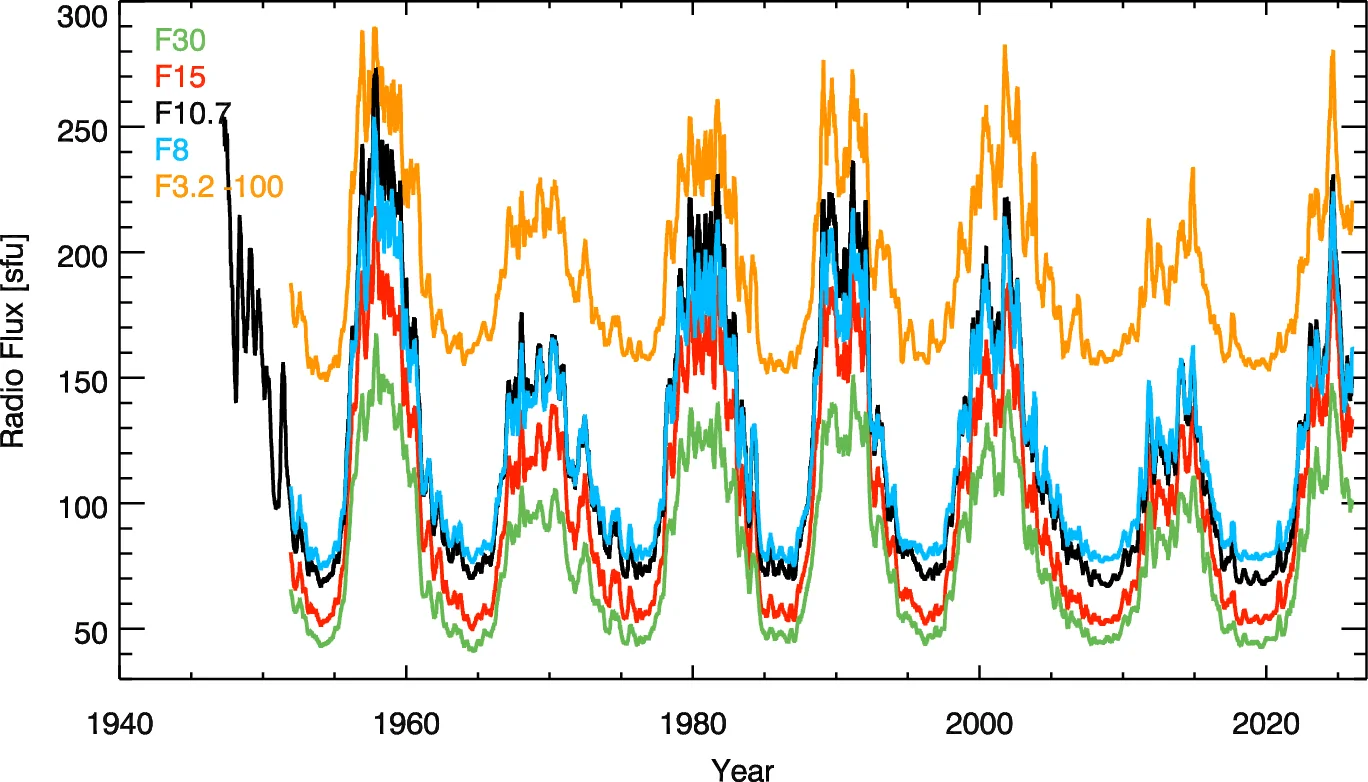
Radio flux measurements. Shown are the Dominion F10.7 series and Nobeyama data for all other ones. The values shown are adjusted to one AU and are 81-day running means. F3.2 has been reduced by 100 sfu to bring it closer to the other indices

### Solar Radio Flux measurements

Solar radio emission offers valuable insights into the structure and dynamics of the solar atmosphere above the temperature minimum region (Shibasaki et al. 2011). Systematic monitoring of solar radio emissions began in the late 1940 s, following the first detection of solar radio bursts during World War II (Southworth 1945; Hey 1946). Canadian physicist Arthur E. Covington was a pioneer in this field, initiating observations of solar radio flux centred at 10.7 cm (2.8 GHz) with a 100 MHz-wide band, using surplus radar equipment in 1946 (Covington 1947, 1948). By February 14, 1947, a long-term, daily monitoring program was established in Ottawa, which was later relocated to Algonquin in 1962 and finally to the Dominion Radio Astrophysical Observatory near Penticton, British Columbia, in 1990 (Tapping 2013). These measurements led to the creation of the F10.7 index, which quantifies the total radio emission from the solar disc over a one-hour interval, expressed in solar flux units (sfu), which are defined as 10$^{-22}$ W m$^{-2}$ Hz$^{-1}$. The F10.7 index is available continuously from 1947 to the present day (Fig. 28) and it is one of the most widely used solar activity indices. Solar radio flux can be decomposed into different components distinguished by the time scales of their variability. Rapid flux enhancements associated with solar flares, occurring on time scales from seconds to hours, constitute the rapid (R-)component. The slowly varying (S-)component is linked to active regions and their evolution, and therefore varies on time scales of days or longer (e.g. Tapping and Charrois 1994; Tapping and Zwaan 2001). The F10.7 index is widely used as a proxy for EUV irradiance in modelling the thermosphere and ionosphere, as well as in studies of space weather and climate (see, e.g. Qian and Mursula 2025, and references therein).

In Japan, a long-term program of multi-frequency solar flux observations began in 1951 at the Toyokawa observatory, initially at 8 cm (3.75 GHz, F8, since 1951), and was soon expanded to include additional frequencies: 30 cm (1 GHz, F30, since 1957), 15 cm (2 GHz, F15, since 1957), and 3.2 cm (9.4 GHz, F3.2, since 1956), 1.7 cm (17 GHz), 8.6 mm (35 GHz, since 1983), and 3.7 mm (80 GHz, since 1984) (Nakajima et al. 1985). Routine monitoring at these wavelengths continued at Toyokawa until 1994, when operations were transferred to the Nobeyama Radio Observatory, operated by the National Astronomical Observatory of Japan (Nakajima et al. 1985; Shimojo and Iwai 2023).^31^ Together, the radio fluxes at 30 cm, 15 cm, 10.7 cm, 8 cm, 3.2 cm, 1.7 cm, 8.6 mm, and 3.7 mm provide a multi-frequency view of solar radio variability. The 30 cm, 15 cm, 8 cm, and 3.2 cm are shown in Fig. 28.

Besides these, routine radio observations of the Sun at multiple wavelengths have been carried out at several observatories over the past decades. Multi-wavelength measurements were performed at Yunnan, covering wavelengths of 7.5, 10.7, 15, and 21 cm (Zhang et al. 1993; Zhiguo et al. 1999). At Huancayo Observatory, regular measurements of the F3.2 cm flux began in 1966 (Ishitsuka et al. 2007). Additional monitoring at metric wavelengths was initiated at Úpice Observatory, which began routine observations at 29.5 MHz in March 1964, added measurements at 27.5 MHz in 1965, and later at 32.8 MHz in 1972 (Klimeš et al. 1999). Since 1982, the Learmonth Solar Observatory has provided continuous multi-frequency solar flux measurements at fixed frequencies of 245, 410, 610, 1415, 2695, 4995, 8800, and 15400 MHz (corresponding to wavelengths from about 1.2 m to 1.95 cm), recorded with a cadence of 1 s. Further information on additional observatories conducting solar radio observations can be found at the Community of European Solar Radio Astronomers (CESRA) website.^32^

**Fig. 29:**
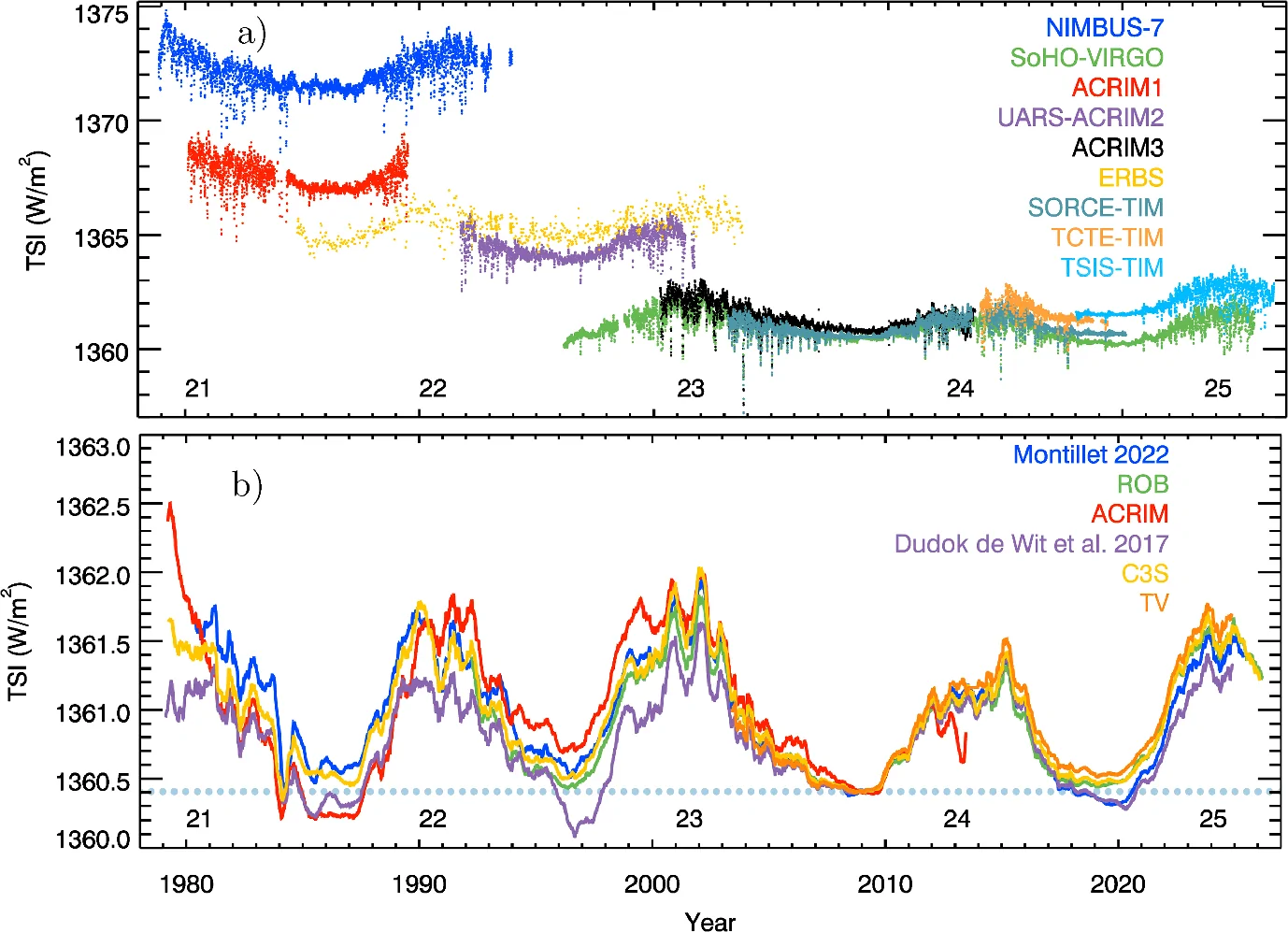
Direct TSI measurements. *Panel a):* TSI measurements from individual instruments. Shown are daily values. *Panel b):* Composites of direct TSI measurements. Shown are 180-day running means. All series are offset to match the value of the Montillet et al. (2022) TSI composite over the 2008 activity minimum (marked with the dotted cyan line). The numbers at the lower part of the figure denote the conventional solar cycle numbering

### Solar irradiance measurements

Solar irradiance is the radiative output of the Sun as measured at the top of Earth’s atmosphere and normalised to the mean Sun-Earth distance. It strongly depends on wavelength, with the wavelength resolved solar irradiance being termed SSI, and the wavelength-integrated one the total solar irradiance (TSI). Atmospheric absorption hampered early attempts to measure TSI and SSI from ground, while TSI was typically mentioned as solar constant due to the notion that it remains unchanged. Instruments onboard satellites made it possible to measure TSI and SSI since 1978 and 1968, respectively. However, the temporal and spectral coverage of SSI before the 2000 s is rather limited (Ermolli et al. 2013; Kopp 2025). A detailed overview of the existing TSI and SSI measurements is provided in the review by Kopp (2025).

Since the late 1970 s, more than ten instruments have measured TSI, with some of the longest-running records illustrated in Fig. 29a. Most individual instruments operated for approximately one solar cycle or less; however, at any given time, observations were typically maintained by two to three instruments, ensuring near-continuous coverage. Because measurements from different missions exhibit systematic discrepancies, cross-calibration is required to construct a consistent long-term record. Different calibration approaches adopted by various teams have led to multiple TSI composite series, the principal ones of which are presented in Fig. 29b. The composites are namely: Montillet et al. (2022), Dudok de Wit et al. (2017),^33^ Copernicus Climate Change Service (C3S),^34^ ROB^35^ (named after Royal Observatory of Belgium, previously referred to as RMIB, Royal Meteorological Institute of Belgium; Dewitte et al. 2004; Dewitte and Nevens 2016), TV (Time Variance; Coddington et al. 2025; Harber and Coddington 2025), and ACRIM^36^ (Active Cavity Radiometer Irradiance Monitor, which is the instrument taken as the reference by Willson 1997; Willson and Mordvinov 2003). Most TSI composites undergo regular updates, whereas the ACRIM composite has not been officially updated since 2013.

A key issue in constructing these TSI composites is the so-called ACRIM-gap (July 1989 to October 1991), which arose from the delayed launch of ACRIM2 experiment onboard the Upper Atmospheric Research Satellite (UARS) following the Challenger disaster (Katzoff 1986). During this interval, the only instruments measuring TSI were Earth Radiation Budget onboard the Nimbus-7 satellite (Nimbus-7/ERB; Hickey et al. 1980) and the NASA (National Aeronautics and Space Administration) Earth Radiation Budget Experiment onboard the Earth Radiation Budget Satellite (ERBE/ERBS; Barkstrom 1984), which indicate differing trends. As a result, the ACRIM composite, favouring the original Nimbus-7/ERB data, shows an increase in TSI between the 1986 and 1996 solar minima. In contrast, other composites apply corrections to the Nimbus-7/ERB measurements and find little to no change in TSI between these minima (Fröhlich 2000; Chatzistergos et al. 2023b). More recent analyses suggest that the apparent discrepancy associated with the ACRIM gap likely originates from residual unaccounted artefacts in the Nimbus-7/ERB data. In particular, Amdur and Huybers (2023, 2025) performed a statistical assessment of the ACRIM-gap using a Bayesian framework. Their method employs explicitly defined priors that do not assume the superiority of any particular satellite dataset or solar proxy for bridging the gap. Using nearly all available satellite observations, they infer a slight downward trend in TSI since 1978, arguing against the increase implied by the ACRIM composite and aligning instead with the majority of existing TSI composites. Furthermore, TSI reconstructions with physics-based semi-empirical models (see Sec. 4.4) also disfavour an increase in irradiance across the ACRIM-gap (e.g., Krivova et al. 2009; Chatzistergos et al. 2025a).

There are no equivalent composites for SSI, however, because SSI measurements are much more limited than TSI ones in both their temporal and spectral coverage (Ermolli et al. 2013; Kopp 2025). An important long-term SSI record is the composite Lyman-α irradiance series compiled by Machol et al. (2019),^37^ which extends back to 1977 based on direct observations. To produce a continuous record, data gaps in this composite were filled using proxy reconstructions based on the Mg ii index and the F10.7 and F30 radio flux indices.

## Reconstructions

In this section, I review studies that reconstruct solar observations and activity metrics. Such reconstructions are particularly important because they can extend solar activity records to periods lacking direct measurements. That includes extensions of disc-integrated indices (Sect. 4.1) as well as image to image conversions (Sect. 4.2). Two particular cases are presented separately: the reconstruction of magnetograms (Sect. 4.3) and the reconstruction of irradiance variations (Sect. 4.4).

**Fig. 30:**
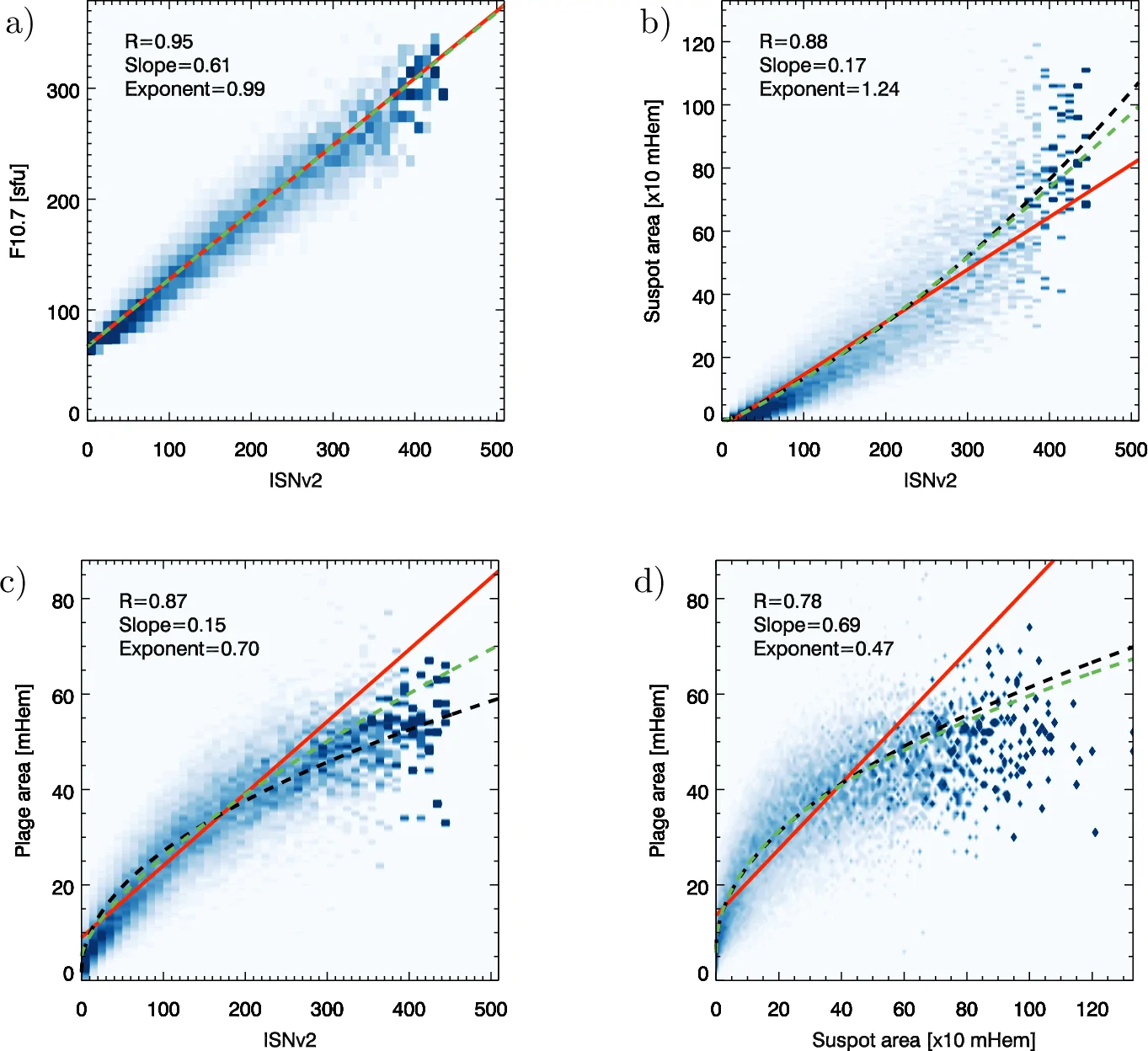
Comparison between solar activity indices. Shown are colour-coded density functions (white indicates zero density and dark blue the saturation level, set to 0.3 for panel a and 0.1 for all other panels). The index pairs are: ISNv2 versus F10.7 (panel a), ISNv2 versus sunspot areas from Mandal et al. (2020, panel b), ISNv2 versus plage areas from Chatzistergos et al. (Chatzistergos et al. (2020c), panel c), and sunspot areas from Mandal et al. (2020) versus plage areas from Chatzistergos et al. (2020c, panel d). Both plage and sunspot areas correspond to projected areas not corrected for foreshortening. Each panel also shows a linear fit (red line) and a power-law fit (green dashed curve); in panels c and d a quadratic fit is additionally included (black), while in panel b a second-degree polynomial fit (black). The Pearson linear correlation coefficient, *R*, the slope of the linear fit, and the exponent of the power-law fit are also provided in each panel

### Conversions of disc-integrated indices

For many applications (e.g. irradiance reconstructions; Sect. 4.4), it is important to extend the temporal coverage of existing solar activity series. The most straightforward way to do this is by exploiting empirical relationships with other longer records. Several studies have attempted to derive such relationships between pairs of activity indices, most commonly involving sunspot numbers, sunspot areas, and Ca ii K plage areas. For instance, a linear relationship between Ca ii K plage areas from Brussels and the TIGRE (Telescopio Internacional de Guanajuato Robotico Espectroscopico; Schmitt et al. 2014) S-index was reported by Vanden Broeck et al. (2024). Similarly, Reda et al. (2024) found a linear relationship between Ca ii K and Mg ii index. Lean et al. (2001); Lean (2018) used Ca ii K plage areas from the McMath Solar Telescope and Big Bear Solar Observatory, together with the HoSc98 GSN series, to extend their chromospheric activity index, based on the Mg ii index, back to 1950 and 1610, respectively. Murabito et al. (2023) used SST (Scharmer 2006) Ca ii K data, degrading the spectral and spatial resolution to match those of historical instruments, and derived relationships to transform Ca ii K  emission profiles obtained under different observational conditions (passband and spatial resolution).

In the following, we discuss studies that have derived empirical relationships between sunspot number, sunspot areas, Ca ii K plage areas, Ca ii K network areas, and the F10.7 index. In principle, such empirical relations have also been used to reconstruct irradiance variability, however, this will be discussed separately in Sect 4.4.

#### Sunspot numbers versus sunspot areas

Sunspot areas provide a more physical measure of solar activity than sunspot numbers; however, photographic sunspot area records are currently available only from 1874 onward. Consequently, several studies have derived empirical relationships between sunspot numbers and sunspot areas to extend the latter back in time. Wilson (1984); Wilson and Hathaway (2005, 2006); Vaquero et al. (2004); Hamidi et al. (2012); Hathaway (2015) reported a linear relationship between ISNv1 and sunspot areas from RGO and Rome at annual cadence. In particular, Hathaway (2015) derived a relationship $A=16.7 R_z$, where *A* is the sunspot area. Hathaway (2015) also reported that the scaling parameter becomes 11.2 when the AAVSO sunspot number series is used (see Sect. 3.1.2). Vaquero et al. (2004) reported a linear relationship between RGO sunspot areas and the HoSc98 GSN series, while Aparicio et al. (2014) found a similar linear relationship between Madrid sunspot numbers and the Balmaceda et al. (2009) sunspot area record. Xanthakis (1960) noted the non-linearity between ISNv1 and sunspot areas and derived relationships to convert annual ISNv1 into sunspot areas. These relationships were expressed as a linear function of ISNv1 modulated by $\cos ^2k$, where *k* is the number of years from the cycle maximum. Li et al. (2016) and Tapping and Morgan (2017) found that a second-degree polynomial provides a better fit, an approach also adopted by Temaj et al. (2026b). In contrast, Berrilli et al. (2020) reported a power-law relationship between ISNv2 and sunspot areas from San Fernando and MLSO/PSPT. Similarly, Carrasco et al. (2016) derived a power-law relationship between the Balmaceda et al. (2009) sunspot area record and ISNv2. Figure 30b compares daily values from ISNv2 to the sunspot area series of Mandal et al. (2020) with a linear, a power law, and a second-degree polynomial fit to the data. Figure 30b shows that the relationship is notably non-linear, with the second-degree polynomial and power-law fits performing equally well.

#### Sunspot number and area versus Ca ii K plage areas

The unavailability of plage area records prior to the 1890 s, together with the difficulties encountered in recent decades in deriving reliable plage area measurements, has motivated several studies to establish empirical relationships between plage areas and sunspot areas or sunspot numbers, thereby enabling the extension of plage area records further back in time (see e.g. Chatzistergos et al. 2022a andreferencestherein). The reported relationships exhibit significant differences, partly due to the choice of sunspot number series and the selection of the Ca ii K archive used to derive plage areas (since different passbands probe different heights in the solar atmosphere and thus yield different plage area estimates; see Sect. 3.3). Additional discrepancies arise from methodological choices related to image processing and segmentation.

In particular, Dorotovic et al. (1996); Foukal (1996); Fligge and Solanki (1998); Kuriyan et al. (1982) studied the relationship between Ca ii K plage areas and ISNv1, while Bertello et al. (2016); Priyal et al. (2017); Singh et al. (2021, 2022); Chatzistergos et al. (2022a) between ISNv2. All previous studies have found that, for monthly and annual averages, the relationship is effectively linear, although with differing parameter values. The results diverge more substantially, however, when daily values are considered. For daily Ca ii K plage areas versus sunspot number series, Singh et al. (2021, 2022) reported a linear relationship, whereas Foukal (1996); Fligge and Solanki (1998) found that a quadratic relation provides a better description. In contrast, Chatzistergos et al. (2022a) concluded that a power-law relationship yields the best fit to the data. Chatzistergos et al. (2022a) also compared plage areas with the CEA17 GSN series, finding qualitatively similar relationships to those obtained with ISNv2. It should be noted that most previous studies relied on different, typically single, Ca ii K archives, whereas Chatzistergos et al. (2022a) analysed 38 archives in addition to the composite plage area series of Chatzistergos et al. (2020c). They further showed that the parameters of the inferred relationships depend on the passband used in the Ca ii K observations. Figure 30c compares daily values from the composite plage area series of Chatzistergos et al. (2020c) and ISNv2 along with a linear, a power law, and a quadratic fit to the data.

A relationship between plage and sunspot areas was derived by Foukal and Vernazza (1979); Foukal (1993, 1996, 1998); Schatten et al. (1985); Oster (1986); Lawrence (1987); Tlatov et al. (2009); Singh et al. (2021, 2022); Chowdhury et al. (2022); Mandal et al. (2017a); Chapman et al. (1997, 2001, 2011); Steinegger et al. (1996a); Chatzistergos et al. (2020b, 2022a). However, the results are more heterogeneous than those obtained when comparing plage areas with sunspot numbers. Singh et al. (2021, 2022), using RGO sunspot areas together with Kodaikanal, Mount Wilson, and Mauna Loa plage areas, reported a linear relationship for both monthly and daily values. In contrast, Foukal (1996; 1998, using Mount Wilson and NGDC plage areas and RGO sunspot areas) found a quadratic relationship for monthly averages, but a linear one for annual values. Tlatov et al. (2009) also reported a quadratic relationship between plage areas (derived from Kodaikanal and Mount Wilson archives) and RGO sunspot areas; however, they obtained significantly different parameters for the two archives, suggesting a dependence of the relationship on the Ca ii K bandpass. Similarly, Chowdhury et al. (2022, using Kodaikanal Ca ii K plage areas and RGO sunspot areas) and Chapman et al. (1997, using San Fernando plage and sunspot areas) reported quadratic relationships for both monthly and daily values. A different approach was taken by Preminger and Walton (2007), who modelled the response of plage areas to sunspot areas as a linear transformation, expressed as the convolution of sunspot areas with a finite impulse response (FIR) filter:

6 $$\begin{aligned} h(t)=\textrm{max}[\exp {(-t/\tau )}\cos {(2\pi t/ 27)},0], \end{aligned}$$

where τ a free parameter, while *t* denotes time in integer-day steps.

Finally, Chatzistergos et al. (2020c, using 38 Ca ii K archives and the composite series of Chatzistergos et al. 2020c together with the sunspot areas of Mandal et al. 2020) found that quadratic or power-law relationships provide the best description, even for annual averages. They demonstrated a clear dependence of the plage-sunspot area relationship on the Ca ii K bandpass, and further reported small variations in the relationship that depend on the level of solar activity (Chatzistergos et al. 2022a). Figure 30d compares daily values from the composite plage area series of Chatzistergos et al. (2020c) and the sunspot areas of Mandal et al. (2020) along with a linear, a power law, and a quadratic fit to the data. Figure 30d shows that the power-law exponent is very close to 0.5; therefore, the power-law and quadratic fits return very similar results.

#### Sunspot number versus Ca ii K network areas

As discussed in Sect. 3.4.1, fewer studies have derived network areas from Ca ii K data compared to plage areas. Consequently, to my knowledge, only three studies have examined relationships between Ca ii K network areas and sunspot number series. Singh et al. (2021, 2023) reported linear relationships between Kodaikanal Ca ii K active and enhanced network areas (defined using fixed contrast thresholds) and ISNv2. Singh et al. (2021) reported correlation coefficients of 0.87 and 0.77 for active and enhanced network, respectively. Subsequently, Singh et al. (2023) reported correlation coefficients of 0.64, 0.84, 0.76, and 0.79 for the total network derived from Kodaikanal spectroheliograms (1905–2007), Kodaikanal filtergrams (1997–2013), Mount Wilson spectroheliograms (1915–1985), and MLSO/PSPT filtergrams (1998–2015), respectively. Berrilli et al. (2020) reconstructed monthly network areas back to 1749 using a power-law relationship with ISNv2.

#### Sunspot numbers versus F10.7

Because the F10.7 index is widely used as a proxy for EUV irradiance in thermosphere and ionosphere modelling, as well as in space weather and climate studies (see e.g., Qian and Mursula 2025 andreferencestherein), extending the record before 1947 is necessary. Many studies have used sunspot number series (ISNv1 or ISNv2) to extend F10.7 index to the past. Various empirical relationships have been presented for this, such as linear (Xanthakis and Poulakos 1985; Hathaway et al. 2002), polynomials (second degree: Kuklin 1984; third degree: Zhao and Han 2008; Thompson 2010; Tiwari and Kumar 2018; and fourth degree: Clette 2021), power law (Johnson 2011), as well as a linear with an exponential correction (Holland and Vaughan 1984) and second-degree polynomial with a saturating exponential correction (Tapping and Valdés 2011; Tapping and Morgan 2017). Figure 30a compares daily values from F10.7 and ISNv2 along with a linear and a power law fit to the data.

The previous relationships assume a one-to-one correspondence between F10.7 and sunspot numbers, despite the fact that sunspots and faculae evolve differently, with faculae persisting longer than sunspots. Preminger and Walton (2007) introduced an alternative approach to account for this difference. They modelled the response of F10.7 to sunspot areas as a linear transformation, expressed as the convolution of sunspot areas with a FIR filter (Eq. 6). Yeo et al. (2020a) modified the approach of Preminger and Walton (2007) by introducing a power-law relationship and by removing the skewness of the first lobe of the FIR filter toward negative values present in the original formulation. In particular, the FIR applied by Yeo et al. (2020a) is:

7 $$\begin{aligned} h(t)=\textrm{max}\left[ \exp {(-|t|/\tau )}\cos {(2\pi t/ 27)},0\right] , \end{aligned}$$

Yeo et al. (2020a) applied this approach by comparing F10.7 to the Balmaceda et al. (2009) sunspot areas, ISNv1 and ISNv2 as well as the HoSc98 and CEA17 GSN.

Extensions of activity indices can also be achieved with machine learning approaches converting other indices. Caution is warranted when using machine learning or other methods to predict indices beyond the next solar cycle, due to the inherently chaotic nature of solar activity (e.g. Cameron and Schüssler 2019; Petrovay 2020; Usoskin 2023). One application of machine learning for reconstructing, but not predicting or extrapolating, F10.7 was presented by Belbase et al. (2026). They employed a Long Short-Term Memory (LSTM) model to reconstruct F10.7 using a combination of ISNv2, Mandal et al. (2020) sunspot areas, and the Chatzistergos et al. (2020c) Ca ii K  plage area composite.

**Fig. 31:**
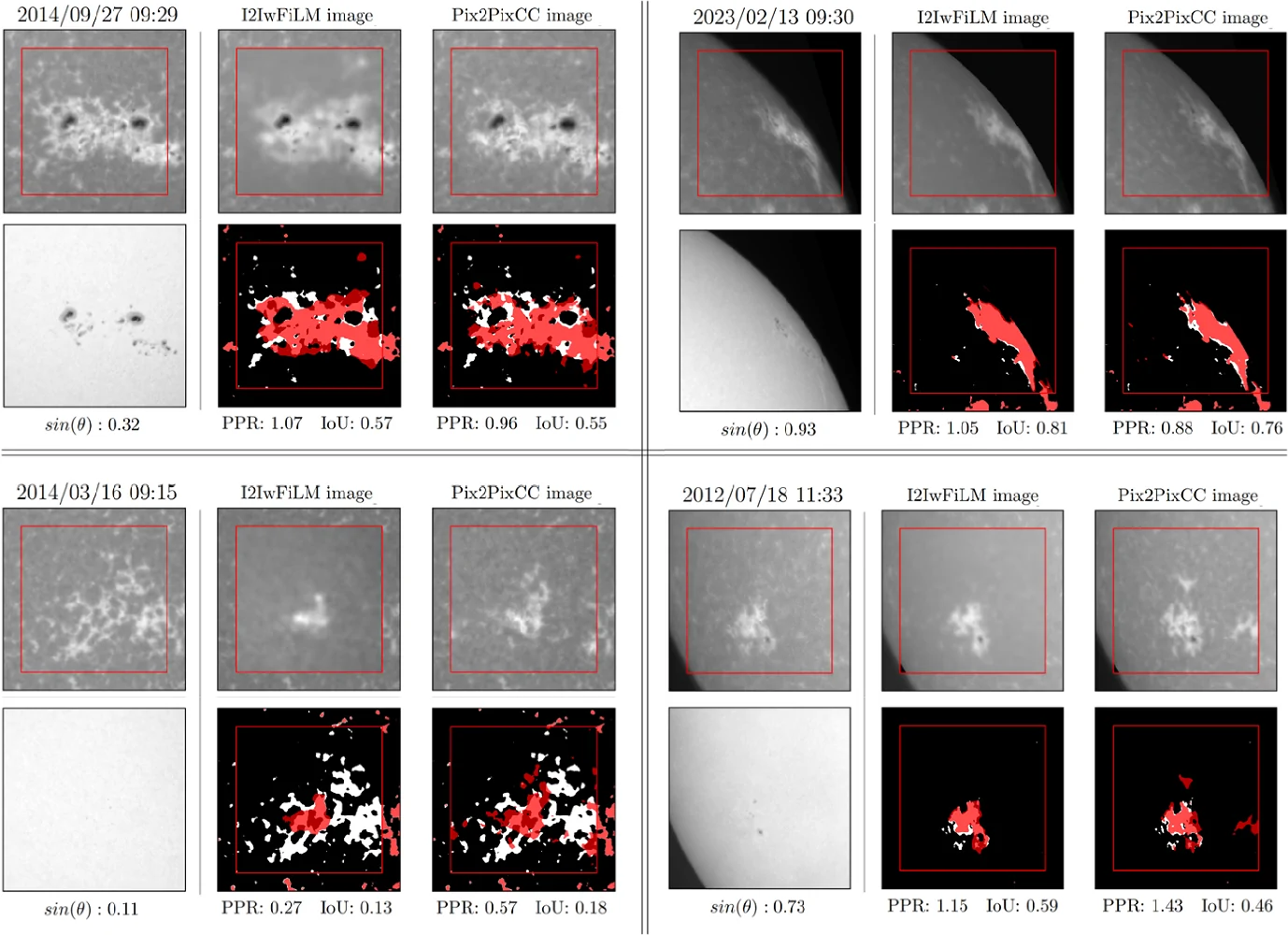
Conversion of Brussels white-light to Ca ii K images by Sayez et al. (2025). Shown are four cases of plage regions. In each case is shown the original Ca ii K and white-light images (top and bottom left), along with the reconstructed Ca ii K images with FiLM (top middle) and Pix2Pix (top right), while below the reconstructed images it gives the masks of plage regions (white for the original Ca ii K and red for the respective reconstructed one). Below the images it also gives $\sin \theta$, as well as metrics of the conversion: plage pixel ratio (PPR; the ratio of plage pixels in generated and original images) and the intersection over union (IoU; being the ratio between the number of pixels that belong to both masks and the number of pixels that belong to at least one mask). Adapted from Sayez et al. (2025)

### Image-to-image conversions

The availability of modern, high-quality data combined with advanced processing techniques has recently made it possible to convert solar spatially-resolved observations into different observational forms. These developments include transforming historical drawings into monochromatic or white-light observations as well as altering the wavelength and passband characteristics of monochromatic data. This has the potential to extend full-disc solar observation datasets to wavelengths that do not exist in the historical record, while also facilitating the homogenization of existing datasets and the correction of problematic or inconsistent data.

Deep learning approaches have been used for image-to-image (I2I) translations (see review by Ramos et al. 2023), mostly through the “pix2pix” model and its variants (Isola et al. 2017; Wang et al. 2018) or FiLM (Feature-wise Linear Modulation; Perez et al. 2018). For instance, various studies performed I2I conversions between different wavelength EUV images (Salvatelli et al. 2022; Jarolim et al. 2025; Schirninger et al. 2025). Salvatelli et al. (2022) demonstrated that although EUV-to-EUV wavelength conversion models can attain high overall performance, they exhibit significant limitations when applied to highly energetic phenomena like solar flares. Son et al. (2021) used “pix2pixHD” to get He i images from 193 and 304 Å  SDO/AIA images, while Liu et al. (2021) used the “pix2pix” model to convert GONG Hα observations to EUV images as acquired by SDO/AIA over 94, 131, 171, 193, 211, 304, 335, and 1600 Å. Liu et al. (2021) reported best performance of their approach for 304 and 1600 Å images and mostly for the large-scale features. Sayez et al. (2025) compared the performance of “pix2pix” and FiLM to convert Brussels white-light images to Ca ii K ones. Examples of their calibration are shown in Fig. 31. They reported that although “pix2pix” produces more visually attractive results, FiLM yielded fewer hallucinations than “pix2pix”. Both models, however, performed worse near disc centre when converting white-light images to Ca ii K due to the limited information of faculae near disc centre in white-light.

A different approach was applied by Yadav et al. (2026), who aimed at deriving conversion relationships between Ca ii K observations taken with different bandpasses. This is an important step to allow homogenising the various historical Ca ii K archives we discussed in Sect. 2.3.1 which have been performed with quite diverse observational setups. In particular, Yadav et al. (2026) used high spectral and spatial resolution observations in the Ca ii K line from the Sunrise-3 mission (Korpi-Lagg et al. 2025; Solanki et al. 2026) to emulate different passbands and derive empirical contrast-contrast relationships between them. They found a power law to describe this relationship best and provided coefficients to convert Ca ii K images for various combinations of passbands in the range of 0.15 and 9 Å and spatial resolutions of 1 to 6”. The parameters they used cover all of the existing historical Ca ii K archives. By applying the derived relationships to ground-based observations from Rome/PSPT and Meudon, they demonstrated that the method can be successfully applied to real observations for homogenisation purposes. To date, neither the machine learning approaches nor the method of Yadav et al. (2026) have been applied to I2I conversions using long historical archives. However, they offer considerable potential for the homogenization and extension of these records.

### Magnetogram reconstructions

One of the most important applications of historical solar observations is that they can be used to recover information about the solar surface magnetic field over periods that magnetograms did not exist. Considering that solar magnetograms only began in the 1960 s (though with sufficient accuracy only since the 1970 s, see Sect. 2.2.3), white-light, Ca ii K , and Hα  data can extend them back to 1610, 1892, and 1909, respectively. Furthermore, EUV observations of the solar far side enable the reconstruction of magnetograms for regions that are not directly observable from Earth. To date, large-scale magnetogram reconstructions have relied almost exclusively on Ca ii K  observations, while data in other spectral lines or in white light have been more limited and explored only through machine-learning-based I2I translation methods (see Sect. 4.2).

There have been a few attempts to apply machine learning approaches to reconstruct LOS magnetograms from sunspot drawings. In particular, Lee et al. (2021) showed the potential of deep-learning models based on conditional generative adversarial networks to reproduce LOS magnetic field maps from sunspot drawings. Lee et al. (2021) used sunspot drawings from Mount Wilson observatory between 2011 and 2015 to compare with SDO/HMI LOS magnetograms. Lee et al. (2021) also reported that they achieve a 0.82 linear correlation between the total magnetic flux of the reconstructed and original SDO/HMI LOS magnetograms. This approach was then applied on Galileo’s drawings from 2 June to 8 July 1612, while similar approaches were applied by Uneme et al. (2022) based on a drawing by Derfflinger from 27 September 1802 during the Dalton Minimum and Lee et al. (2019) who used Carrington’s drawing of the Carrington event over 1 September 1859. Overall, such reconstructions are expected to be rather uncertain, due to lack of information about the polarity of the regions, the extend and morphology of faculae, especially those not associated with sunspots, while there are also issues due to precise cross-calibration of sunspot areas to modern standards due to the acuity of the observers or drawing styles. For instance, Lee et al. (2021) reported that the reconstructed LOS magnetograms included bipolar magnetic regions that mostly followed Hale’s polarity law and that this approach is not able to identify anti-Hale regions. Lee et al. (2021) also cautioned that since their training was limited to magnetograms over one solar cycle, it is very likely that it will not accurately reproduce the polarity inversion over subsequent cycles. Furthermore, Uneme et al. (2022) acknowledged that due to the drawing style of Derfflinger the reported areas are potentially overestimated by a factor of about 8. This value was approximated by comparing the distribution of sunspot sizes to those from the Debrecen sunspot area database, however it is highly uncertain considering that the drawings might be more qualitative rather than quantitative and thus this factor might be varying for different images. As a result, Uneme et al. (2022) provided 4 different estimates of LOS magnetograms based on different estimates on the underestimation of the sunspot area in Derfflinger’s drawings. More recently, Lee et al. (2026) used the pix2pixHD model to reconstruct a LOS magnetogram for 1 September 1859, during the Carrington event, based on Carrington’s sunspot drawing. Rather than using the original drawing directly, they first converted it into a binary mask in which each sunspot was represented by a circle centred on its mean position and with a radius determined by its area. The method was evaluated using approximately 7000 Debrecen sunspot drawings paired with SoHO/MDI and SDO/HMI magnetograms, yielding an error of about 16% in the recovered total unsigned magnetic flux.

Kim et al. (2019) and Jeong et al. (2022) applied similar machine learning approaches to reconstruct SDO/HMI-like LOS magnetograms from STEREO/Extreme UltraViolet Imager (EUVI; Kaiser 2005) 304 Å observations. However, the availability of EUV observations only during the space era limits the applicability of this approach for extending magnetograms far back in time. Nevertheless, it enables the reconstruction of magnetograms for the far side of the Sun due to the availability of such data from space-based missions like STEREO.

With EUV observations restricted to the space era and white-light and continuum observations lacking sufficient information on faculae, Ca ii K observations offer the only viable means of reconstructing magnetograms back to the late nineteenth century. The importance of full-disc Ca ii K observations for reconstructing magnetograms lies with the excellent correspondence between bright regions in Ca ii K and regions with strong magnetic field in magnetograms. There were some very early evidence about the connection between Ca ii K brightness and magnetic field strength Babcock and Babcock (1955); Howard (1959); Leighton (1959), which led to numerous studies to explore their relationship (e.g. Frazier 1971; Skumanich et al. 1975; Schrijver et al. 1989; Nindos and Zirin 1998; Harvey and White 1999; Rast 2003; Ortiz and Rast 2005; Rezaei et al. 2007; Loukitcheva et al. 2009; Kahil et al. 2017, 2019; Chatzistergos et al. 2019d ). Most of these studies found that a power-law relationship best describes the connection between Ca ii K intensity and magnetic field strength. However, Kahil et al. (2017, 2019) reported that a logarithmic function provided a better fit to their data. These discrepancies can be attributed to significant variation in the datasets used, differences in observational periods, spectral bandwidths, and targeted solar regions (quiet Sun vs. active regions). Due to this heterogeneity, the derived power-law exponents vary substantially, typically ranging from 0.3 to 0.6. The most extensive such study to date is the one by Chatzistergos et al. (2019d). They employed high-quality, full-disc Rome/PSPT Ca ii K observations and SDO/HMI LOS magnetograms spanning nearly half a solar cycle to study the Ca ii K -magnetic field relationship. They also reported no significant dependence of the Ca ii K -magnetic field relationship on either μ or the level of solar activity, within the uncertainty of their fits. Chatzistergos et al. (2019d, 2021, 2025a) further applied this relationship to reconstruct unsigned magnetograms from 13 Ca ii K archives, including historic photographic datasets from Mount Wilson and Meudon.

A different set of reconstructed magnetograms was produced by Mordvinov et al. (2020). Mordvinov et al. (2020) used Carrington maps over 1907–1965 produced from Kodaikanal Ca ii K and Hα data processed by Chatterjee et al. (2016, 2017a). They applied a polynomial relationship between Ca ii K contrast and magnetic flux density, with the polynomial’s degree varying based on solar activity (third degree for low activity, fourth for high). However, this dependence on activity level could be an artefact introduced by the image processing (see Sect. 2.6.3). The Hα maps were used to assign polarities to the reconstructed magnetograms.

Pevtsov et al. (2016b) reconstructed LOS magnetograms from historical photographic Ca ii K data by also incorporating sunspot magnetic field measurements by Mount Wilson. They used low-resolution Carrington maps based on photometrically uncalibrated Mount Wilson Ca ii K observations processed by Bertello et al. (2020). Their method relied on an empirical relationship between the area of plage regions and magnetic flux. Plage regions were identified using a 2σ brightness threshold, and a single magnetic field strength was assigned per region. Polarity information was inferred using the Mount Wilson sunspot catalogue. Limitations of these data include the maps’ low resolution, coarse quantization of field strength, and lack of information on fields outside bright plage areas.

Finally, Shin et al. (2020) employed a deep learning approach, using an I2I translation model (“pix2pixHD” by Wang et al. 2018) to convert CCD-based Rome/PSPT Ca ii K images into synthetic magnetograms resembling those from SDO/HMI. Their results demonstrated a high linear correlation (0.99) between the total unsigned magnetic flux in the generated and actual magnetograms. However, pixel-by-pixel correlation varied: it averaged 0.74, with higher accuracy in active regions (0.81) and lower in quiet Sun regions (0.24). Notably, they used data without compensating for the limb-darkening, which may have impacted the model’s performance.

A different empirical approach has also been used to estimate the polar magnetic field. In particular, the polar magnetic field, on the scale of Wilcox measurements, has been inferred from a linear relationship applied on Mount Wilson white-light and SoHO/MDI continuum polar facular counts covering 1906–2010 (see Sect. 3.2; Sheeley 1964, 1976, 1991, 2008; Muñoz-Jaramillo et al. 2012). Similar methodologies were applied by Priyal et al. (2014a); Mishra et al. (2025). Specifically, Priyal et al. (2014a) reconstructed polar magnetic fields, on the scale of Wilcox measurements, for 1909–1990 using Kodaikanal Ca ii K observations, from which they considered as polar network regions defined at latitudes beyond 70$^\circ$. Likewise, Mishra et al. (2025) derived polar network areas for 1904–2022 from Kodaikanal and Rome/PSPT Ca ii K observations, which they subsequently converted into polar magnetic field estimates using Wilcox Solar Observatory measurements as a reference. Since Wilcox defines the polar field over latitudes beyond 55$^\circ$, Mishra et al. (2025) computed two separate polar plage series, one beyond 55$^\circ$ and another beyond 70$^\circ$, and used the former for the polar field reconstruction.

### Irradiance reconstructions

Historical solar data are essential for reconstructing irradiance variations prior to the availability of direct measurements. Since the Sun supplies nearly all the energy entering Earth’s climate system (e.g., Kren et al. 2017), having long-term, accurate records of solar irradiance is essential, particularly for studies of climate change (e.g. Solanki et al. 2013). As we saw in Sect. 3.11, TSI and SSI have been measured from space only since 1978 and 1968, respectively. To extend this further back in time, many irradiance models have been developed. The basis of such models is the fact that variations in solar irradiance are primarily driven by the evolution of solar surface magnetism (see e.g. Shapiro et al. 2017; Yeo et al. 2017). Accordingly, modern models reconstruct irradiance changes by accounting for the competing effects of sunspots (which reduce irradiance) and faculae (which enhance it).

Overall, there are two main categories of irradiance models: empirical and physics-based/semi-empirical ones. Empirical models typically scale disc-integrated indices to direct irradiance measurements. One class of these models employs time series of disc-integrated sunspot and facular indices (e.g. Oster et al. 1982; Schatten et al. 1985; Foukal and Lean 1986, 1988; Lean and Skumanich 1983; Lean and Repoff 1987; Lean et al. 1998; Foukal 2002, 2012; Ambelu et al. 2011; Penza et al. 2022, 2024; Chatzistergos et al. 2024; Chapman et al. 1996, 2012, 2013, 2024; Preminger et al. 2002; Chatzistergos et al. 2020a ). Among the various facular indices that have been used are Ca ii K plage areas, Ca ii K intensity, Mg ii index, and F10.7, while various sunspot number series and areas have been used for the sunspot indices. Another class of such models relies solely on sunspot records (e.g. Eddy et al. 1982; Schatten and Orosz 1990; Hoyt and Schatten 1993; Dewitte et al. 2022; Chatzistergos 2024 ).

The second category of irradiance models is physics-based/semi-empirical ones. These models divide the solar surface to different components, typically quiet Sun, faculae, sunspots (sometimes separately as umbra and penumbra), and network. For each component, the models use spectra computed with radiative transfer codes based on corresponding model atmospheres, while solar observations are used to describe the spatial and temporal evolution of the features. White-light and continuum observations are used to extract the information about sunspots positions, while typically magnetograms or Ca ii K observations for faculae and network. Most such available reconstructions cover relatively short periods (Ermolli et al. 2003b; Penza et al. 2003; Ermolli et al. 2011; Criscuoli et al. 2018; Fontenla and Landi 2018; Chatzistergos et al. 2021, 2025a). There are also a few long-term physics-based semi-empirical irradiance reconstructions. Such reconstructions rely either solely on sunspot records or cosmogenic isotopes (Krivova et al. 2007, 2010; Wu et al. 2018; Shapiro et al. 2011; Egorova et al. 2018; Sowmya et al. 2025; Temaj et al. 2026b, a; Chatzistergos et al. 2026).

**Fig. 32:**
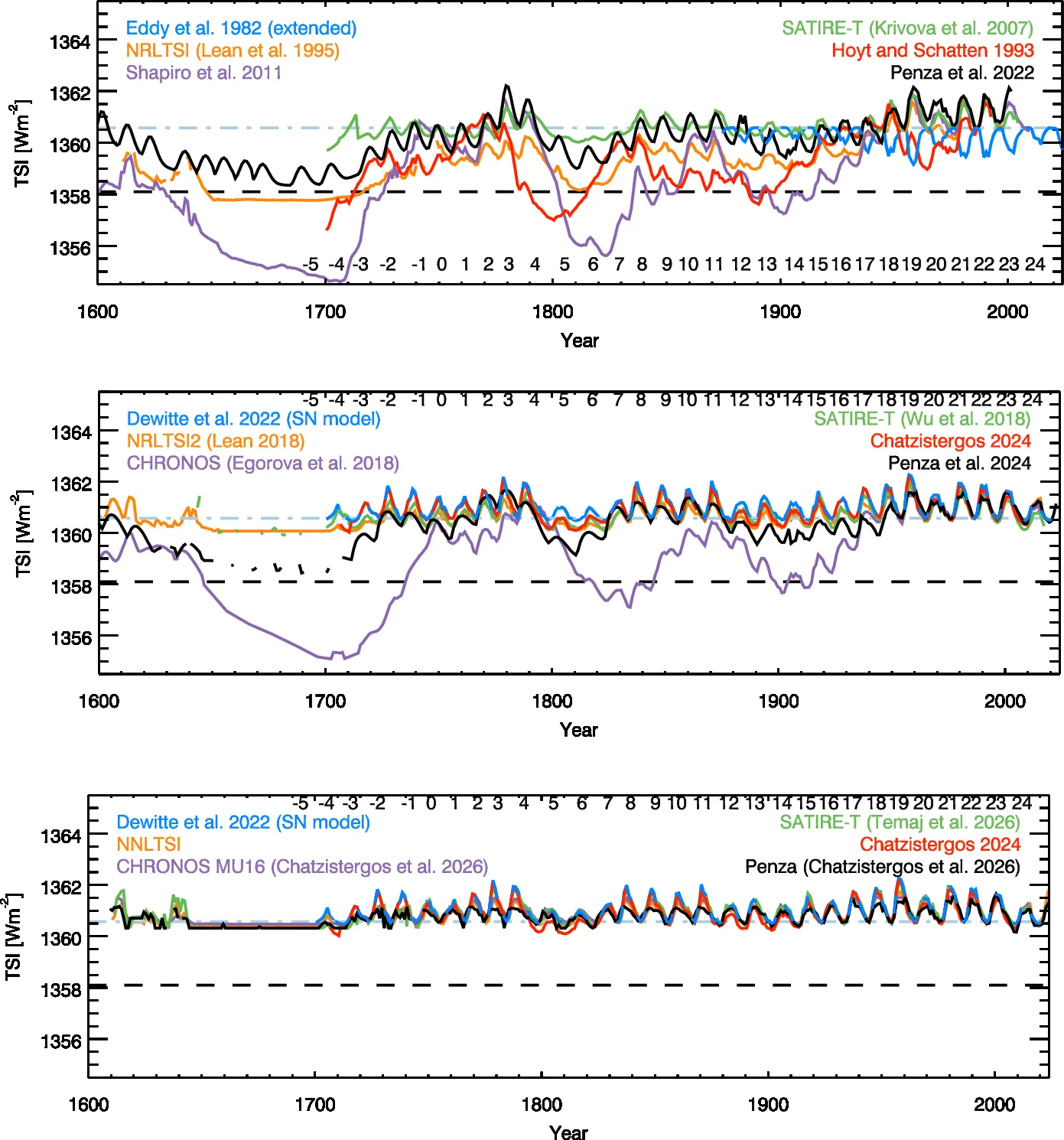
Long-term TSI reconstructions. The three panels show the same models (except in cyan) with the top panel showing the first or an early version of it, the middle panel showing the latest version of the models in 2025, while the bottom panel shows the most recent version of the model after the updates by Chatzistergos et al. (2026). All series have been offset to match the value of the Montillet et al. (2022) TSI composite over the 1986 minimum (marked with the cyan dot dashed horizontal line). Shown are annual means, while the numbers within the panels denote the conventional solar cycle numbering. The black dashed horizontal line marks the Yeo et al. (2020b) constraint on the dimmest state of the Sun

A major challenge for irradiance reconstructions arises from the unavailability of facular data prior to the era of direct TSI measurements. Due to this all long-term TSI reconstructions must infer facular variability through strong and uncertain assumptions based solely on sunspot records or indirect proxies (e.g. cosmogenic isotopes). This has resulted in large uncertainties in the secular trend of irradiance variations, spanning roughly an order of magnitude (for a review, see: Chatzistergos et al. 2023b). Figure 32 shows some of the main long-term TSI reconstructions. The figure is divided into three panels showing roughly the same models: (1) the first or an early version of each model, (2) the most recent version available by 2025, (3) the current version of each model after the updates by Chatzistergos et al. (2026). An exception is made for the models of Eddy et al. (1982) and Dewitte et al. (2022), which are inherently different, but are grouped together because both are empirical approaches that scale sunspot number series. The former, however, being one of the earliest modelling approaches assumed that sunspot variations are in anti-phase with TSI variations. I note that the series by Eddy et al. (1982) in Fig. 32 is extended to the present by linearly scaling the Mandal et al. (2020) sunspot area series. The figure illustrates how advances in our understanding of irradiance modelling, together with improvements in the underlying input data, have led to increased agreement among existing models.

Although the early versions of these models generally suggested a secular increase in TSI between the Maunder Minimum and the present of around or exceeding 2 W m$^{-2}$, by 2025 only two models still indicated such a large secular change: CHRONOS (Code for the High spectral ResolutiOn recoNstructiOn of Solar irradiance; Egorova et al. 2018) and the one by Penza et al. (2024). Subsequent work (Chatzistergos et al. 2026) showed that the large secular trends in both models were artefacts caused by an improper connection of modulation potential series derived from cosmogenic isotope and neutron monitor data, as well as by the scaling applied to convert these series into irradiance. Overall, the current versions of existing TSI reconstructions converge on a relatively modest increase in TSI since the Maunder Minimum, typically of less than 1 W m$^{-2}$, consistent with the theoretical constraint on the dimmest possible state of the Sun proposed by Yeo et al. (2020b), shown in Fig. 32 as dashed horizontal line. (See also Lockwood and Ball 2020). Nevertheless, the exact magnitude of the secular trend remains uncertain, and notable differences persist among the existing reconstructions.

Potential advances and further improvement in the estimate of the secular trend in TSI variations can be achieved with the use of historical Ca ii K observations (Chatzistergos et al. 2024). As a step toward this, Chatzistergos et al. (2021, 2025a) adapted the physics-based semi-empirical Spectral And Total Irradiance REconstructions (SATIRE)-S (‘S’ for satellite era) model, previously driven solely by direct magnetograms, to operate with unsigned magnetograms reconstructed from Ca ii K observations. In particular, Chatzistergos et al. (2025a) incorporated reconstructed magnetograms (see Sec. 4.3) from Meudon, San Fernando, and Rome/PSPT Ca ii K data into SATIRE-S, thereby improving the connection between the magnetogram datasets and yielding a more reliable estimate of the long-term irradiance trend. Thanks to the high quality and consistency of Meudon Ca ii K data (along with Mount Wilson magnetograms also used by Chatzistergos et al. 2025a) over the ACRIM-gap period (see Sect. 3.11), Chatzistergos et al. (2025a) demonstrated that the ACRIM-gap is most likely the result of artefacts in the NIMBUS-7/ERB TSI data.

However, Ca ii K  data still pose significant challenges for their full exploitation in irradiance reconstructions. The advances discussed in the previous sections have laid the groundwork, but substantial effort is still required to realise the full potential of Ca ii K  data for century-long irradiance reconstructions.

## Conclusion

The Sun is by far the most extensively observed astronomical object. In this work, I have attempted to provide an overview of the principal historical solar observations that form the foundation of our current understanding of long-term solar variability. As discussed throughout this review, many of these observations were conducted at a time when the physical processes governing solar activity were not yet well understood. Consequently, these datasets contain valuable information that was either not extracted or not fully appreciated at the time of acquisition. One such example is the key role of Ca ii K  observations in recovering past solar magnetism and reconstructing solar irradiance variations. This makes the reanalysis and homogenisation of historical solar records particularly important, as modern techniques can reveal new insights into long-term solar variability and its drivers.

The societal relevance of such efforts has increased markedly in recent decades, as modern society has become increasingly dependent on electronic infrastructure and space-based technologies that are vulnerable to solar variability and extreme space weather events. Maintaining, extending, and critically reassessing historical solar observations is therefore essential for assessing technological risks associated with extreme solar activity. At the same time, concerns about climate change and the role of solar variability in affecting Earth’s climate underscore the need for accurate, long-term solar records to improve projections of future climate change.

In this review, several tables compiling archives of historical solar observations have been presented. These compilations are not exhaustive and may contain inaccuracies, particularly regarding the exact temporal coverage of individual archives, as well as unavoidable omissions. For example, sporadic Ca ii K observations have been reported from Ulugh Beg since 1967, although no systematic observing program appears to have been established and the present location of these data remains uncertain. Such cases highlight the urgent need to locate, preserve, and digitise remaining historical solar archives before further information is lost. The importance of recovering and digitising historical solar observations has been emphasised repeatedly within the community, including in Resolution B3 of the XXX General Assembly of the International Astronomical Union (Pevtsov et al. 2019; IAU 2022). Continued international coordination and sustained support for archival efforts will be essential to fully exploit the scientific value of these unique long-term records.

## Abbreviations list

The following list contains all abbreviations used throughout this paper.

AAVSO American Association of Variable Star Observers
ACRIM Active Cavity Radiometer Irradiance Monitor
AFPT Automatic Flare Patrol Telescope
AIA Atmospheric Imaging Assembly
AURA Association of Universities for Research in Astronomy, Inc.
BCE Before Current Era
C3S Copernicus Climate Change Service
CATE Continental-America Telescopic Eclipse
CCD Charge-Coupled Device
CEA17 Chatzistergos et al. (2017)
CESRA Community of European Solar Radio Astronomers
CFDT Cartesian Full Disk Telescope
CFI Comprehensive Flare Index
CHASE Chinese Hα Solar Explorer
CHIP Chromospheric Helium-I Imaging Photometer
CHROMIS CHROMospheric Imaging Spectrometer
CHRONOS Code for the High spectral ResolutiOn recoNstructiOn of Solar irradiance
ChroTel Chromospheric Telescope
CI Coronal Index
CLV Centre-to-Limb Variation
CME Coronal Mass Ejection
CMOS Complementary Metal Oxide Semiconductor
CRISP CRisp Imaging Spectropolarimeter
CSIRO Commonwealth Scientific and Industrial Research Organisation
DB Dispartitions Brusques (filament disappearances)
DF Disc Fraction
DKIST Daniel K. Inouye Solar Telescope
EIT Extreme ultraviolet Imaging Telescope
ERB Earth Radiation Budget
ERBE Earth Radiation Budget Experiment
ERBS Earth Radiation Budget Satellite
EUV Extreme UltraViolet
EUVI Extreme UltraViolet Imager
FDP Full Disk Patrol
FiLM Feature-wise Linear Modulation
FIR Finite Impulse Response
FMT Flare Monitoring Telescopes
GOES Geostationary Operational Environmental Satellites
GONG Global Oscillation Network Group
GSN Group Sunspot Number
HMI Helioseismic and Magnetic Imager
HoSc98 Hoyt and Schatten (1998)
I2I Image To Image
IBIS Interferometric BIdimensional Spectrometer
IGY International Geophysical Year
IoU Intersection over Union
IRIS Interface Region Imaging Spectrograph
ISA Israel Space Agency
ISN International Sunspot Number
ISOON Improved Solar Observing Optical Network
ISRO Indian Space Research Organization
ISS Integrated Sunlight Spectrometer
IUP Instituts für UmweltPhysik
KP/512 Kitt Peak observatory 512-channel Babcock-type magnetograph
KP/SPM Kitt Peak observatory CCD SPectroMagnetograph
LOS Line-Of-Sight
LSTM Long Short-Term Memory
MDI Michelson Doppler Imager
MHD MagnetoHydroDynamic
MLSO Mauna Loa solar observatory
NASA National Aeronautics and Space Administration
NGDC National Geophysical Data Center
NOAA National Oceanic and Atmospheric Administration
NRL Naval Research Laboratory
NSF National Science Foundation
NSO National Solar Observatory
ONSET Optical and Near-infrared Solar Eruption Tracer
PHI Polarimetric and Helioseismic Imager
PPR Plage Pixel Ratio
PRO-AM PROffesional-AMateur
PSPT Precision Solar Photometric Telescope
PUNCH Polarimeter to Unify the Corona and Heliosphere
QBSA Quarterly Bulletin on Solar Activity
RGO Royal Greenwich Observatory
RMIB Royal Meteorological Institute of Belgium
ROB Royal Observatory of Belgium
SATIRE Spectral And Total Irradiance REconstructions
SDO Solar Dynamics Observatory
sfu solar flux units
SHG SpectroHelioGraph
SHS SpectroHelioScope
SILSO Sunspot Index and Long-term Solar Observations
SMART Solar Magnetic Activity Research Telescope
SMAT Solar Magnetism and Activity Telescope
SO Solar Orbiter
SODISM Solar Diameter Imager and Surface Mapper
SoHO Solar and Heliospheric Observatory
SOLIS Synoptic Optical Long-term Investigations of the Sun
SOLRAD SOLar RADiation
SOON Solar Observing Optical Network
SORCE SOlar Radiation and Climate Experiment
SSI Spectral Solar Irradiance
SST Swedish 1-m Solar Telescope
STEREO Solar TErrestrial RElations Observatory
SUIT Solar Ultraviolet Imaging Telescope
SvSc16 Svalgaard and Schatten (2016)
SW Spectral Width
TCTE Total solar irradiance Continuity Transfer Experiment
THEMIS Telescope Héliographique pour l’ Etude du Magnétisme et des Instabilités Solaires
TIGRE Telescopio Internacional de Guanajuato Robotico Espectroscopico
TIM Total Irradiance Monitor
TSI Total Solar Irradiance
TSIS Total and Spectral solar Irradiance Sensor
TV Time Variance
UARS Upper Atmospheric Research Satellite
UEA21 Usoskin et al. (2021)
USAF United States Air Force
UV UltraViolet
VHS24 Velasco Herrera et al. (2024)
VIRGO Variability of IRradiance and Gravity Oscillations
VSM Vector-SpectroMagnetograph
WARM White-light Active Region Monitor

## Footnotes

1 https://www.sidc.be/uset/telescopewl.php

2 https://www.esa.int/ESA_Multimedia/Images/2004/03/First_photo_of_the_Sun_1845

3 Some observations can be seen in http://solardatabase.free.fr/tableau_cak.php

4 https://www.specola.ch/en/historical-astronomical-observations-by-s-cortesi/

5 https://solarwww.mtk.nao.ac.jp/en/db_ha1.html

6 Some observations can be seen in http://solardatabase.free.fr/tableau.php

7 https://space-valley.com/

8 https://thue.museum-digital.de/object/566

9 http://www.zam.fme.vutbr.cz/~druck/eclipse/Ecl2009e/0-info.htm

10 https://mlso.hao.ucar.edu/hao-eclipse-archive.php

11 http://www.zam.fme.vutbr.cz/~druck/eclipse/

12 https://www2.hao.ucar.edu/mlso/instruments/mki-k-coronameter

13 https://www2.hao.ucar.edu/mlso/instruments/mlso-chip-helium-imager

14 https://bass2000.obspm.fr/bass2000/data/pub/synoptic/

15 https://www.ngdc.noaa.gov/stp/solar/solar-imagery.html

16 https://www2.hao.ucar.edu/mcintosh-archive

17 https://docs.google.com/spreadsheets/d/1m8h853ljTCFbtd7grcNHFaT0Ari4VoCHLvQTXKSGRKQ/pubhtml

18 https://observethesun.com/

19 https://www.sidc.be/SILSO/datafiles

20 https://www.aavso.org/solar

21 The project is described in https://web.archive.org/web/20250513172140/https://www.aavso.org/solar-observing-project

22 https://www.ngdc.noaa.gov/stp/solar/wfaculae.html

23 https://solarwww.mtk.nao.ac.jp/en/db_faculae.html

24 https://www.ngdc.noaa.gov/stp/SOLAR/ftpcalcium.html

25 https://www.ngdc.noaa.gov/stp/space-weather/solar-data/solar-imagery/photosphere/sunspot-drawings/mcmath/

26 https://solarwww.mtk.nao.ac.jp/en/wdc/qbsa.html

27 https://www.ngdc.noaa.gov/stp/solar/filaments.html

28 https://www.ncei.noaa.gov/products/space-weather/legacy-data/solar-flares-events

29 https://astronomi.bogazici.edu.tr/flare-index

30 Available at: https://www.iup.uni-bremen.de/UVSAT/data/

31 https://solar.nro.nao.ac.jp/norp/index.html

32 https://www.astro.gla.ac.uk/users/eduard/cesra/?page_id=187

33 https://spot.colorado.edu/~koppg/TSI/

34 https://confluence.ecmwf.int/pages/viewpage.action?pageId=304239361

35 https://www.sidc.be/observations/space-based-timelines/tsi

36 https://web.archive.org/web/20170611210135/ http://acrim.com/

37 Available at: https://lasp.colorado.edu/lisird/data/composite_lyman_alpha
